# Workers, capitalists, and the government: fiscal policy and income (re)distribution

**DOI:** 10.1016/j.jmoneco.2021.01.004

**Published:** 2021-04

**Authors:** Cristiano Cantore, Lukas B. Freund

**Affiliations:** a Bank of England, Threadneedle Street, London EC2R 8AH, United Kingdom; b University of Surrey, Guildford, GU27XH, United Kingdom; c University of Cambridge, Sidgwick Avenue, Cambridge CB3 9DD, United Kingdom

**Keywords:** Fiscal policy, Heterogeneity, HANK, TANK

## Abstract

We propose a novel two-agent New Keynesian model to study the interaction of fiscal policy and household heterogeneity in a tractable environment. Workers can save in bonds subject to portfolio adjustment costs; firm ownership is concentrated among capitalists who do not supply labor. The model is consistent with micro data on empirical intertemporal marginal propensities to consume, and it avoids implausible profit income effects on labor supply. Relative to the traditional two-agent model, these features imply, respectively, a lower sensitivity of consumption to the composition of public financing; and smaller fiscal multipliers alongside pronounced redistributive effects.

## Highlights

• Propose a tractable capitalist-worker New Keynesian model to study the interaction of household heterogeneity and fiscal policy.
• Modeling limited asset market participation through portfolio adjustment costs generates a realistic pattern of intertemporal marginal propensities to consume.
• The capitalist-worker structures ensures that the transmission of demand shocks under sticky prices does not rely on income effects on labor supply induced by countercyclical markups.
• Relative to the predictions of the traditional two-agent model, fiscal multipliers are smaller; and the sensitivity of the output path to public deficits is dampened.
• Overall, the model matches the implications of richer Heterogeneous-Agent New Keynesian (HANK) models in key respects, while remaining analytically tractable.

## 1 Introduction

Macroeconomic models with household heterogeneity give rise to aggregate dynamics that can significantly diverge from those implied by their representative-agent counterparts. Heterogeneous-agent New Keynesian (HANK) models, in particular, combine nominal rigidities with a Bewley-İmrohoroǧlu-Huggett-Aiyagari type incomplete markets environment and aggregate risk. As they require keeping track of the full distribution of households across idiosyncratic states, their development has been accompanied by a renewed interest in tractable models able to capture some of the fundamental properties of heterogeneous-agent models (e.g., Debortoli and Galí (2018)).

We contribute to that literature by showing how the prototypical two-agent New Keynesian (TANK) model introduced by Galí et al. (2007) and Bilbiie (2008) can be extended to match the implications of HANK models better in terms of both consistency with micro data and predictions for the macro effects of fiscal policy. We model heterogeneity in households’ intertemporal consumption-savings behavior by allowing one household type, workers, to save in bonds only subject to portfolio adjustment costs, whereas the remaining households, capitalists, are unconstrained in this respect. This setup allow matching estimates from micro data of, and the predictions of multi-asset heterogeneous-agent models for, the intertemporal marginal propensities to consume that shape the private sector’s dynamic response to shocks. Capitalists own all the economy’s firms, but they do not supply labor. This specification ensures that the transmission of demand shocks under sticky prices does not hinge on income effects on labor supply induced by countercyclical profits. We illustrate the macroeconomic properties of the model by showing how it can be used to match and dissect the impulse response of key macroeconomic variables to fiscal policy shocks. Two key results emerge. First, modeling constrained households as having at least partial access to capital markets – rather than none, as is the case in the traditional two-agent model with hand-to-mouth households – renders the output path less sensitive to that of deficits. Second, in the absence of income effects on labor supply from distributed profits, fiscal multipliers are smaller in the new model than the traditional two-agent model. We next describe each of these points in greater detail.

Considering household consumption-savings behavior first, Auclert et al. (2018) demonstrate the importance of intertemporal marginal propensities to consume (iMPCs) in characterizing the general equilibrium effects of demand shocks in models with household heterogeneity and nominal rigidities.^1^ In household data, iMPCs for an unanticipated increase in income are, on average, much higher on impact than permanent income considerations would imply and display a pattern of gradual decay thereafter. Auclert et al. (2018) and Hagedorn et al. (2019b) demonstrate that (multi-asset) HANK models are capable of matching these empirical micro moments, whereas neither the benchmark representative-agent nor the traditional two-agent model can. The former, which conforms to the permanent-income hypothesis, generically under-predicts the sensitivity of consumption to current income. By introducing a share of hand-to-mouth consumers the prototypical TANK model is able to replicate the high average impact-MPC. But this extreme specification of limited asset market participation makes iMPCs drop sharply thereafter.

The first result of this paper is that incorporating portfolio adjustment costs delivers iMPCs that are more consistent with these stylized facts from micro data and the predictions of HANK models. We replace hand-to-mouth households in the two-agent setup with intermediately constrained “workers” who likewise solely rely on labor earnings but who can *partially* smooth consumption through borrowing and saving in government bonds. A convex cost function penalizes workers in case their holdings deviate from some benchmark level effectively controls the degree of financial constraints (as in Schmitt-Grohe and Uribe (2003) and Neumeyer and Perri (2005)). We show analytically that in a simple partial equilibrium setting the implied consumption dynamics following an income windfall are very close to those found in the data. That constrained agents have some access to capital markets rather than none is also more in line with micro-level consumption evidence.

We then turn to households’ intra-temporal tradeoff between labor and leisure. Broer et al. (2020a) show that in the benchmark representative-agent New Keynesian model, the transmission of monetary policy is driven by income effects on labor supply that result from countercyclical variations in firm profits in the presence of sticky prices. They argue that this mechanism is implausible and hard to justify empirically. That same profit income effect on labor supply is more consequential still in the traditional TANK model (Bilbiie (2008)). We break this cyclical connection between profits and the labor supply of firm owners by including a “capitalist” type instead of the usual specification of unconstrained households in two-agent models. Capitalists do not participate in the labor market and, consequently, profits no longer directly enter the optimality condition pinning down aggregate labor supply (cf. Walsh (2017) and Bayer et al. (2020)). We coin the model that embeds the two-agent household block with capitalists and workers in an otherwise canonical New Keynesian environment “TANK-CW” – for capitalist/worker – as distinct from the traditional “TANK-UH” model – with unconstrained/ hand-to-mouth households.

What are the implications of the proposed modeling of household heterogeneity – developed in Section 2 – for the transmission and effects of macroeconomic policy? To answer these questions, in Section 3 we compare and contrast the effects of government spending shocks in both traditional and new TANK models. Two main results stand out. First, the amended labor supply specification delivers smaller fiscal multipliers relative to the predictions of a traditional two-agent model with flexible wages. Second, allowing constrained households to have partial rather than no access to financial markets shapes the sensitivities of output and consumption paths to the composition and timing of fiscal stimulus financing. In particular, whereas the dynamics of these variables according to TANK-UH are very closely tied to the path of deficits, this is not true for TANK-CW.

Related Literature. Beyond the key references cited above, our paper relates to several strands in the literature. In the first instance, the role we see for TANK models is not to ‘compete’ with HANK models à la Kaplan et al. (2018) or Hagedorn et al. (2019b). Instead, they have a different scope of application. Two-agent models may serve as tractable laboratories to study the aggregate consequences of macroeconomic policy in the presence of household heterogeneity and for approximating distributional effects of such policy at a high level. They are also easy to extend, solve, and estimate for the purpose of quantitative applications.

There are several other studies exploring the ability of tractable models to mimic properties of heterogeneous-agent models.^2^ Among these papers, our approach, which is based on a financial friction, shares particular similarities to studies that introduce bonds in the utility function (Hagedorn (2018); Kaplan and Violante (2014); Michaillat and Saez (2019)). In the appendix we show the conditions under which these two approaches are first-order equivalent. An alternative avenue to generating empirically credible iMPCs is taken by Bilbiie (2019a), who extends the traditional two-agent model into a tractable HANK framework that incorporates self-insurance against the risk of having to live hand-to-mouth. Finally, our analysis explicitly abstract from another important feature of HANK models: time-varying precautionary savings behavior due to idiosyncratic risk. Challe (2020) and Acharya and Dogra (2020) develop tractable models capturing this mechanism.

Finally, Debortoli and Galí (2018) identify different channels through which household heterogeneity influences aggregate fluctuations and argue that the margin captured by traditional TANK models – changes in the average consumption gap between constrained and unconstrained households – is quantitatively the most significant one when evaluating monetary policy, preference, and technology shocks. Their result further motivates our attempt to bring the consumption behavior of constrained households better in line with micro data than is the case when they are treated as entirely hand-to-mouth. Relative to Debortoli and Galí (2018), we explicitly aim to match micro data on households’ dynamic consumption behavior and focus on fiscal policy.^3^

## 2 A tale of two TANK models

We start by outlining a benchmark version of the two-agent New Keynesian (TANK) framework. Drawing on the more recent heterogeneous-agent literature, we then identify two key limitations of that model in its characterization of household heterogeneity, relating to consumption dynamics and labor supply, respectively. We then show step-by-step how to address these issues by introducing portfolio adjustment costs and capitalists into the model.^4^ Throughout, time is discrete and denoted t=0,1,2,…. Steady-state variables are without time subscript. Real quantities are in terms of the final consumption good and denoted by lower case letters, unless otherwise stated.

### 2.1 Two-agent New Keynesian models

#### 2.1.1 Outline of the benchmark model

Our point of departure is the canonical TANK model pioneered by Galí et al. (2007) and Bilbiie (2008). We consider the simplest possible setup to maximize transparency. As in the latter paper we abstract from physical capital; given our interest in fiscal policy we follow the former in incorporating a fiscal sector.

Households. There is a unit mass of households indexed by i∈[0,1]. A fraction λ behaves in hand-to-mouth fashion due to limited asset market participation (indexed by H). The remainder are unconstrained (indexed by U). Both types share preferences characterized by the period utility function $u=\left(\log\left(c_{t}^{i}\right)-\nu \frac{{\left(n_{t}^{i}\right)}^{1+\varphi }}{1+\varphi }\right),$ where $c^{i}$ and $n^{i}$ denote consumption and hours worked, respectively. The inverse Frisch elasticity of labor supply is parameterized by φ, and ν weights the disutility of working. All households’ labor inputs are bundled by a union that sets wages on their behalf according to a wage schedule $w_{t}=\nu c_{t}n_{t}^{\varphi },$ where $c_{t}\equiv \int_{0}^{1}c_{t}^{i}di$ and $n_{t}\equiv \int_{0}^{1}n_{t}^{i}di$.^5^ Unconstrained households earn not only labor income but also receive dividends, $d_{t},$ distributed by monopolistically competitive firms. These households can also trade in government-issued bonds that pay a nominal gross return $R_{t}$. Lastly, both types of agents are subject to a scheme of lump-sum taxes to finance government purchases. As in Bilbiie (2019b) we impose that total real net taxes $t_{t}$ are split such that constrained and unconstrained households each pay (or receive) a fraction of total taxes proportional to their population share, that is, $t_{t}^{i}=t$ for i=U,H.

Firms. A competitive final goods sector aggregates differentiated intermediate goods according to a CES technology, the elasticity of substitution being η. These intermediates are produced by monopolistically competitive firms. Their pricing decisions are subject to quadratic adjustment costs à la Rotemberg, parameterized by ξ, giving rise to nominal rigidity and a conventional New Keynesian Phillips curve. As in Bilbiie (2019b), the government levies an optimal subsidy $\tau^{S},$ financed through lump-sum taxes on all firms, that induces marginal cost pricing in steady state.

Government. The fiscal authority finances real spending, $g_{t},$ which follows an AR(1) process, by issuing one-period bonds and levying lump-sum taxes as described above. The financing mix to support spending is determined by a tax rule as in Galí et al. (2007). Monetary policy sets the short-term nominal interest rate, $R_{t},$ according to a Taylor rule, with a coefficient $\phi^{\pi }$ on inflation.

The first main column of Table 1 summarizes the equilibrium equations for this benchmark model. To ease exposition and analysis, we log-linearize the model around a steady state with zero inflation, no government spending or debt, and total output normalized to unity. Variables with “ ^ ” denote proportional deviations from their respective steady-state, while “ $\tilde{}$ ” indicates deviations from the steady-state value of total income.

**Table 1:** Log-linearized equilibrium conditions.

Description	Equations: UH		Equations: CW
Euler equation U/C		${\hat{c}}_{t}^{U/C}=E_{t}{\hat{c}}_{t+1}^{U/C}-{\hat{r}}_{t}$	
Budget constraint U/C	${\hat{c}}_{t}^{U}+{\tilde{b}}_{t}^{U}={\hat{n}}_{t}+{\hat{w}}_{t}+\frac{{\tilde{d}}_{t}}{1-\lambda }-{\tilde{t}}_{t}+R{\tilde{b}}_{t-1}^{U}$		${\hat{c}}_{t}^{C}+{\tilde{b}}_{t}^{C}=\frac{{\tilde{d}}_{t}}{1-\lambda }-{\tilde{t}}_{t}+R{\tilde{b}}_{t-1}^{C}$
Euler equation W	-		${\hat{c}}_{t}^{W}=E_{t}{\hat{c}}_{t+1}^{W}-{\hat{r}}_{t}+\psi^{W}{\tilde{b}}_{t}^{W}$
Budget constraint H/W	${\hat{c}}_{t}^{H}={\hat{n}}_{t}+{\hat{w}}_{t}-{\tilde{t}}_{t}$		${\hat{c}}_{t}^{W}+{\tilde{b}}_{t}^{W}=\left({\hat{n}}_{t}^{W}+{\hat{w}}_{t}\right)n^{W}-{\tilde{t}}_{t}+R{\tilde{b}}_{t-1}^{W}$
Aggregate consumption		${\hat{c}}_{t}=\lambda {\hat{c}}_{t}^{H/W}+\left(1-\lambda \right){\hat{c}}_{t}^{U/C}$	
Labor supply	${\hat{n}}_{t}=\varphi^{-1}\left({\hat{w}}_{t}-{\hat{c}}_{t}\right)$		${\hat{n}}_{t}^{W}=\varphi^{-1}\left({\hat{w}}_{t}-{\hat{c}}_{t}^{W}\right)$
Dividends		${\tilde{d}}_{t}=-{\hat{w}}_{t}$	
Phillips curve		${\hat{\Pi }}_{t}=\beta E_{t}{\hat{\Pi }}_{t+1}+\frac{\eta }{\xi }{\hat{w}}_{t}$	
Gov. budget constraint		${\tilde{b}}_{t}=R{\tilde{b}}_{t-1}+{\tilde{g}}_{t}-{\tilde{t}}_{t}$	
Gov. spending		${\tilde{g}}_{t}=\rho^{g}{\tilde{g}}_{t-1}+\epsilon_{t}^{g}$	
Fiscal rule		${\tilde{t}}_{t}=\phi^{\tau t}{\tilde{t}}_{t-1}+\phi^{\tau B}{\tilde{b}}_{t}+\phi^{\tau G}{\tilde{g}}_{t}$	
Taylor rule		${\hat{R}}_{t}=\phi^{\pi }{\hat{\Pi }}_{t}$	
Fisher equation		${\hat{r}}_{t}={\hat{R}}_{t}-E_{t}{\hat{\Pi }}_{t+1}$	
Bond holdings	${\tilde{b}}_{t}=\left(1-\lambda \right){\tilde{b}}_{t}^{U}$		${\tilde{b}}_{t}=\lambda {\tilde{b}}_{t}^{W}+\left(1-\lambda \right){\tilde{b}}_{t}^{C}$

*Notes:* This table summarizes the log-linearized equilibrium conditions of the TANK-UH and TANK-CW models, where “UH” stands for “unconstrained and hand-to-mouth households,” while CW refers to “capitalist and worker households.”

#### 2.1.2 Capitalist-worker TANK model

While tractable and useful in many applications, the macroeconomic literature has pinpointed two distinct problems confronting the traditional two-agent model. Both relate to the specification of the household block. They concern households’ intertemporal consumption-savings choices and the intra-temporal trade-off between labor and leisure. For one thing, the implied consumption dynamics are inconsistent with key stylized facts from micro data. For another, the transmission of demand shocks hinges on implausible profit income effects on labor supply.^6^

We next elaborate on these two limitations, one after the other, and propose a solution that involves two amendments to the household block. We coin the resulting two-agent model a “capitalist-worker” model (abbreviated “CW”). In the proposed model, workers have partial access to financial markets, whereas hand-to-mouth households were fully excluded; and firm ownership is concentrated among capitalists who do not supply labor. The model matches, firstly, both data and multi-asset HANK models in terms of intertemporal marginal propensities to consume. Secondly, the transmission of shocks under sticky prices does not rely on income effects on labor supply induced by countercyclical profits. To facilitate a direct comparison between the two models, the second main column of Table 1 shows the log-linearized equilibrium conditions of the new model.

### 2.2 Consumption dynamics and financial market access

Matching the empirical pattern of intertemporal marginal propensities to consume (iMPCs) is important for understanding the aggregate effects of macroeconomic policy such as fiscal stimulus measures. In particular, Auclert et al. (2018) demonstrate that in a number of important theoretical benchmark cases – with no capital, fully demand-determined labor, and passive monetary policy – iMPCs are sufficient statistics for the general equilibrium effects of demand shocks such as government purchases.^7^ To fix ideas, suppose household behavior can be summarized by an aggregate consumption function $c_{t}\left(\left\{y_{s}-t_{s}\right\}\right),$ so that consumption in any period t depends only on the path of post-tax income in every time period s, where y is pre-tax income and t are net taxes. Then the goods market clearing condition $y_{t}=c_{t}\left(\left\{y_{s}-t_{s}\right\}\right)+g_{t}$ implies a fixed point in the path of output. And the impulse response of output to a change in fiscal policy crucially depends on the iMPC matrix M of partial derivatives of aggregate consumption with respect to after-tax income $x_{s}$ at date s, a typical element being $M_{t,s}=\partial c_{t}/\partial x_{s}$. Specifically, and to first order, total differentiation yields the intertemporal Keynesian cross equation: $dy_{t}=dg_{t}+\sum_{s=0}^{\infty }M_{t,s}\left(dy_{s}-dt_{s}\right)$. Intuitively, the iMPCs fully characterize the interaction of the household block with the rest of the economy. Consequently, within the benchmark environment of Auclert et al. (2018), matching the iMPC moments produced by heterogeneous-agent models, in particular, is sufficient to replicate their predictions for the aggregate impact of public demand shocks.

**Fig. 1:**
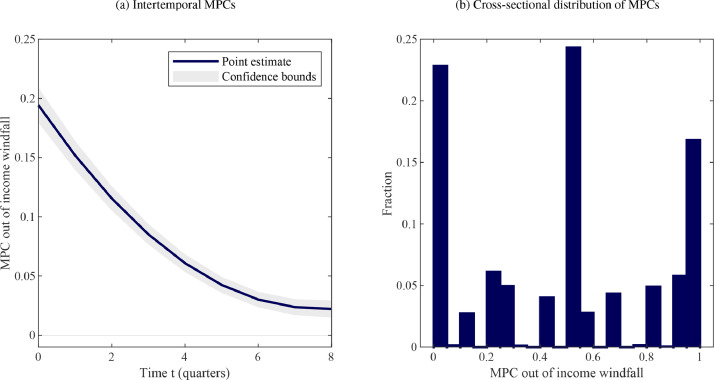
MPCs in the Data. *Notes:* The left panel shows the dynamic consumption response of a household to an unanticipated income shock estimated by Fagereng et al. (2018) and analyzed in Auclert et al. (2018). The quarterly figures are constructed from the original annual data by fitting a cubic spline through the cumulative annual iMPC values. This procedure is also applied to the bounds, ignoring any noise due to the interpolation procedure. The horizontal axis shows time measured in quarters. The vertical axis displays the marginal propensity to consume out of unanticipated income $\partial c_{t}/\partial x_{0}$. The right panel replicates Fig. 1 from Jappelli and Pistaferri (2014) using data distributed through openICPSR. It describes the distribution of self-reported MPCs out of an unexpected income shock in the 2010 Italian Survey of Household Income and Wealth (SHIW).

What do empirical estimates of these iMPCs look like, and how do theoretical models compare? Regarding the first question, Fig. 1(a) summarizes the findings of Fagereng et al. (2018) who estimate households consumption responses to lottery winnings using Norwegian administrative data. Following an unanticipated temporary increase in disposable income the average household’s consumption jumps up, with a quarterly point estimate for $\partial c_{0}/\partial x_{0}$ of approximately 0.2. Importantly, the iMPCs remain elevated thereafter, displaying a pattern of gradual decay. Cumulatively over the first four quarters after the shock, the average households consumes around half of the windfall.

**Fig. 2:**
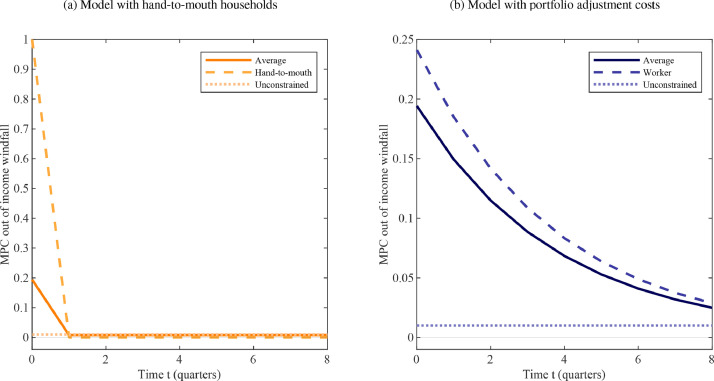
Theoretical iMPCs for an unanticipated income windfall. *Notes:* The figure shows the dynamic, partial equilibrium consumption response to an unanticipated income shock in alternative two-agent models that feature, respectively, a fraction of hand-to-mouth (left panel) or worker households. The horizontal axis shows time measured in quarters. The vertical axis displays the marginal propensity to consume $\partial c_{t}^{i}/\partial x_{0}^{i}$. The parameter choices are explained in Section 3.1.

Auclert et al. (2018) find that among popular modeling approaches, only heterogeneous-agent models with multiple assets can match such empirical estimates of iMPCs. The traditional representative-agent and two-agent models, in particular, cannot replicate these dynamics. Consider Fig. 2(a), which describes a household’s consumption response to an unanticipated, one-off income raise according to a partial equilibrium consumption-savings choice problem.^8^ As a temporary windfall barely affects lifetime resources, under the permanent income hypothesis individual iMPCs are flat at a low level (dotted line).^9^ On the other hand, if a household is hand-to-mouth, such that consumption moves one-for-one with disposable income, the impact MPC is unity but subsequent consumption is entirely unaffected (dashed line). The traditional TANK-UH features a fraction λ∈[0,1] of hand-to-mouth households, while the remainder are unconstrained. By varying λ it is straightforward to match the fairly high average impact effect found in the data (solid line). But the iMPCs sharply drop in the following periods – contrary to what the micro evidence suggests. Hence, Auclert et al. (2018, p.17) diagnose: “Due to the absence of intermediately constrained agents, [the traditional two-agent model] cannot generate elevated iMPCs in year one and later, which are a key characteristic of the data.”

The solution offered in this paper is to model precisely such an intermediately-constrained household, thereby generalizing the stark form of “limited asset market participation” characteristic of the traditional two-agent model. To this end, we substitute hand-to-mouth households for “workers” who likewise do not own any firm equity, but who *can* participate in financial markets subject to convex bond portfolio adjustment costs (“PACs,” for short). Foreshadowing our key result, Fig. 2(b) indicates that incorporating this financial friction implies iMPCs that are in line with the stylized facts from micro data and, hence, the predictions of multi-asset heterogeneous-agent models.

To develop this argument as transparently as possible, consider the partial equilibrium consumption-savings problem facing a worker household. Given processes for per-period income and the real interest rate, $\left\{x_{t}^{W},r_{t}\right\},$ where $1+r_{t-1}=R_{t-1}/\Pi_{t},$ she maximizes the present discounted value of lifetime utility $E_{0}\sum_{t=0}^{\infty }\beta^{t}u\left(c_{t}^{W}\right)$.^10^ As in Section 2.1, the period utility function is of log form.^11^ Unlike a hand-to-mouth household, the worker is able to borrow and save in bonds. The asset value carried into period t is $b_{t-1}^{W}$. Different from the unconstrained type of household, however, workers’ savings choices are subject to a cost. The household is penalized when their holdings deviate from some benchmark level; the strength of this financial friction is indexed by $\psi^{W}$. The adjustment cost takes a simple quadratic form and the target-level is equal to the steady-state value $b^{W}$. The per-period budget constraint accordingly reads

(1) $$b_{t}^{W}+\frac{\psi^{W}}{2}\frac{{\left(b_{t}^{W}-b^{W}\right)}^{2}}{x^{W}}=x_{t}^{W}+\left(1+r_{t-1}\right)b_{t-1}^{W}+f_{t}-c_{t}^{W},\;t=0,1,2,\ldots .$$

Thus, the cost of increasing bond holdings by one unit is greater than unity because it includes the marginal cost of adjusting the size of the portfolio.^12^ To rule out any wealth effects, the costs are rebated to the workers as a lump-sum, $f_{t},$ without this being taken into account by workers when making savings decisions. Scaling the adjustment cost by steady-state income $x^{W}$ ensures comparability across different model specifications.

Solving the household’s problem for the optimal choice of a process for consumption and bond holdings ${\left\{c_{t}^{i},b_{t}^{i}\right\}}_{t=0}^{\infty }$ yields the Euler equation

(2) $$u^{\prime }\left(c_{t}^{W}\right)=\beta E_{t}u^{\prime }\left(c_{t+1}^{W}\right)\frac{\left(1+r_{t}\right)}{1+\left(\psi^{W}/x^{W}\right)\left(b_{t}^{W}-b^{W}\right)}.$$

Equation (2) thus features an endogenous, multiplicative wedge ${\left(1+\left(\psi^{W}/x^{W}\right)\left(b_{t}^{W}-b^{W}\right)\right)}^{-1}$ that is not present in the ‘standard’ Euler equation of an unconstrained household. That latter case is nested for $\psi^{W}=0,$ whereas if $\psi^{W}\to \infty$ the worker household behaves in hand-to-mouth fashion.

It proves instructive to consider a log-linear approximation of the household’s optimal consumption-savings behavior. As before, the Taylor approximation is done around a steady-state with zero inflation, no debt, and total income normalized to unity.^13^ The Euler equation and budget constraint (upon canceling out adjustment costs and rebate) can then be written as

(3) $${\hat{c}}_{t}^{W}=E_{t}\left[{\hat{c}}_{t+1}^{W}-{\hat{r}}_{t}\right]+\psi^{W}{\tilde{b}}_{t}^{W},$$

(4) $${\tilde{b}}_{t}^{W}={\hat{x}}_{t}+\beta^{-1}{\tilde{b}}_{t-1}^{W}-{\hat{c}}_{t}^{W}.$$

We can sharply characterize the worker household’s behavior following an income shock up to first order by solving the system formed by equations (3) - (4), together with the usual transversality condition. Given $E_{t}\left[{\hat{r}}_{l}\right]=0$ for all t and l, it holds that

(5) $${\tilde{b}}_{t}^{W}=\mu_{1}{\tilde{b}}_{t-1}^{W}-\sum_{l=0}^{\infty }\mu_{2}^{-(1+l)}E_{t}\left[{\hat{x}}_{t+l+1}^{W}-{\hat{x}}_{t+l}^{W}\right],$$

where $\mu_{1}=\frac{1}{2}(1+\beta^{-1}+\psi^{W}-\sqrt{{\left(1+\beta^{-1}+\psi^{W}\right)}^{2}-\beta^{-1}})$ is the stable root, satisfying $|\mu_{1}|<1$ whenever $\psi^{W}>0,$ while $\mu_{2}=\left(1+\beta^{-1}+\psi^{W}\right)-\mu_{1},$ such that $|\mu_{2}|>1,$ guaranteeing convergence toward zero (see Appendix A.1 for derivations). Consumption can then be computed from the budget constraint and we can analytically characterize iMPCs as follows.

Proposition 1 **iMPCs for an unanticipated income shock**

*Following an unanticipated one-off income windfall the response of a worker household’s consumption on impact is*

(6) $$\frac{d{\hat{c}}_{0}^{W}}{d{\hat{x}}_{0}^{W}}=1-\mu_{2}^{-1}.$$

*The subsequent expected path of consumption, for* t≥1 *obeys*

(7) $$\frac{E_{0}\left[d{\hat{c}}_{t}^{W}\right]}{d{\hat{x}}_{0}^{W}}=\mu_{1}^{t-1}\left(\beta^{-1}-\mu_{1}\right)\mu_{2}^{-1}.$$

*For* $\psi^{W}\to \infty ,$ *the roots* $\mu_{1}=0$ *and* $\mu_{2}\to \infty ,$ *so that the worker’s consumption response reduces to that of a hand-to-mouth household.*

Proof See Appendix A.1. □

Fig. 2 (b) illustrates that a model with both permanent-income consumers and workers is capable of producing consumption dynamics following an unanticipated income windfall that are close to those found in the data (see Section 3.1 for details on the parameterization). For workers the marginal impact effect on consumption of an income windfall is high, but not equal to unity, and instead of dropping to zero thereafter, the iMPCs remain elevated for several periods (dashed line). Thus, even if the models with hand-to-mouth households and PACs of intermediate strength are parameterized to produce the same impact MPC – as is deliberately done here –, the subsequent iMPC shape is in line with the empirical evidence only in the latter case.

Why exactly does the introduction of portfolio adjustment costs help with matching iMPCs? To gain intuition, notice that the left-hand side of the Euler equation (2) represents the marginal cost of saving, whereas the right-hand side captures the marginal benefit of doing so. The presence of PACs implies that the marginal benefit is declining in the level of savings in an approximately proportional way as it exceeds the steady-state benchmark, the proportionality factor being $\psi^{W}$. Thus, following an increase in disposable income, the household’s consumption smoothing motive pushes towards saving more than in steady state. At the same time, however, the presence of the adjustment cost wedge in the denominator on the right-hand side of Equation (2) reduces the marginal benefit of savings, and more so the higher those excess savings already are. Hence, PACs push the household to consume more in the present. It is the interplay of these two countervailing forces that determines the household’s allocation of the extra income between consumption and saving at the margin.

Two further properties of the consumption model with portfolio adjustment costs warrant highlighting. The first is that introducing an intermediately constrained household type has implications also for the dynamic consumption response to shocks that are anticipated to materialize several periods into the future. We can again make this point precise by characterizing the model’s predictions analytically as follows.

Proposition 2 **iMPCs for an anticipated income shock**

*The response of consumption when news arrives at* t=0 *of a one-off income windfall that materializes* s≥0 *periods later is*

(8) $$\frac{d{\hat{c}}_{0}^{W}}{E_{0}\left[d{\hat{x}}_{s}^{W}\right]}=\mu_{2}^{-s}\left(1-\mu_{2}^{-1}\right).$$

*The subsequent expected path of consumption, for* t≥1 *obeys*

(9) $$\begin{array}{cc} \frac{E_{0}\left[d{\hat{c}}_{t}^{W}\right]}{E_{0}\left[d{\hat{x}}_{s}^{W}\right]} & =\left\{ \begin{array}{cc} \mu_{2}^{-s}\left(1-\mu_{2}^{-1}\right)\times (\mu_{2}^{t}-\left(\beta^{-1}-\mu_{1}\right)\mu_{1}^{t-1}\sum_{l=1}^{t}{\left(\frac{\mu_{1}}{\mu_{2}}\right)}^{1-l}),\; & \text{for}\;t\leq s\\ \mu_{1}^{t-(s+1)}\left(\beta^{-1}-\mu_{1}\right)(\mu_{2}^{-1}-\left(1-\mu_{2}^{-1}\right)\sum_{l=1}^{s}{\left(\frac{\mu_{1}}{\mu_{2}}\right)}^{l}),\; & \text{for}\;t>s, \end{array}\right. \end{array}$$

*where if* s=0 *the empty sum is treated as equal to zero, as is convention.*

Proof See Appendix A.1. □

Intuitively, for $\psi^{W}>0$ the responsiveness of current consumption to shocks to future income is more modest the farther away they lie in the future. This behavior is different from both the unconstrained household (who spend a constant fraction of their present-value lifetime income) and hand-to-mouth consumers (who consume their disposable income every period).

Fig. displays household partial equilibrium responses to an income windfall that is anticipated to materialize three periods into the future. The traditional, UH model of limited asset market participation eliminates dynamic anticipation effects almost entirely. This is because of households’ limited ability (in the case of the hand-to-mouth) or desire (for permanent-income consumers) to respond to increases in future income. The same observation applies to the intertemporal path of iMPCs conditional on past income shocks (Bilbiie (2019a)). By contrast, the predictions of a model with intermediately constrained households yields consumption dynamics that are closer to those of a HANK model as studied in Hagedorn et al. (2019b). All households in our two-agent model act on expectations for future income changes, but there exists important heterogeneity across types in terms of how this manifests in current consumption. Specifically, being subject to a friction that interferes with their ability to fully smooth consumption, workers likewise consume considerably more in period t=3 when the positive income shock materializes. Yet they also borrow against the future prior to the windfall and save some of it afterwards.

To further develop this point, combine Euler equation and budget constraint to obtain

(10) $${\hat{c}}_{t}^{W}=\frac{1}{1+\psi^{W}}\left[E_{t}{\hat{c}}_{t+1}^{W}-{\hat{r}}_{t}\right]+\frac{\psi^{W}}{1+\psi^{W}}{\hat{x}}_{t}+\frac{\psi^{W}}{1+\psi^{W}}\beta^{-1}{\tilde{b}}_{t-1}^{W}.$$

From equation (10) we see that for $\psi^{W}\in \left(0,\infty \right)$ current consumption is a function not only of expected consumption and the expected real interest rate, as it would be according to the permanent income-hypothesis. Instead, it also depends on current income and savings from last period. The latter component encodes the differences in past savings behavior of the worker household relative to the extreme case of pure hand-to-mouth consumption when $\psi^{W}<\infty$.

Thus, equation (10) also indicates that the interest elasticity of workers’ consumption is lower than for permanent-income households – but greater than that of hand-to-mouth households, for whom it is zero. Moreover, the effect of an announced interest rate cut is decreasing in the horizon of the announced cut, in line with the findings of Hagedorn et al. (2019a). This property is pertinent vis-à-vis the literature on the “forward guidance puzzle,” which has highlighted that the standard Euler equation has the implausible implication that news about future real interest rate changes at any horizon has the same effect on current consumption as an equally large change to the current interest rate (see Del Negro et al. (2015), among others). In similarity to the “discounted Euler equation” of McKay et al. (2017), for a worker household the effect of interest rates l periods ahead on current consumption is discounted by a factor $\mu_{2}^{-l}$ where $|\mu_{2}|\geq 1$.^14^ Intuitively, when the worker household reacts to news about an interest rate cut in the distant future by borrowing and consuming more in the present, she has to pay a cost for deviating from her target level of savings in each period prior to the rate cut being realized. Hence, relative to a permanent-income household she reacts less to such news. Overall, both the muted sensitivity of consumption to news about future interest rates and the greater responsiveness to variations in current income are characteristic of heterogeneous-agent models relative to their representative-agent counterparts (Kaplan and Violante (2018)).

**Fig. 3:**
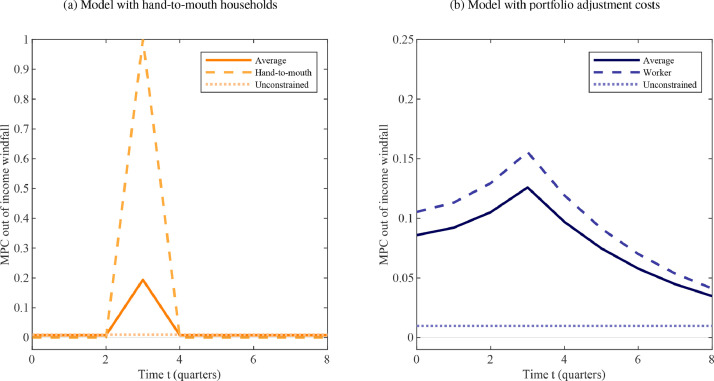
Theoretical iMPCs for an anticipated income windfall. *Notes:* The figure shows the dynamic, partial equilibrium consumption response to a three-period ahead income shock in alternative two-agent models that feature, respectively, a fraction of hand-to-mouth (left panel) or worker households. The horizontal axis shows time measured in quarters. The vertical axis displays the marginal propensity to consume $\partial c_{t}^{i}/\partial x_{3}^{i}$. The parameter choices are explained in Section 3.1.

A final observation is that although by construction no two-agent setup can match the full cross-sectional distribution of MPCs, the proposed model is closer to the data than the traditional setup.^15^ Fig. 1(b) illustrates, based on Jappelli and Pistaferri (2014), that even though there is significant dispersion in the cross-sectional data, there is a noticeable bunching around intermediate values. This appears to be a robust feature across data sources. Figs. 2(a) and 3(a), meanwhile, indicate that the UH model implies an extreme degree of heterogeneity in MPCs in the period of the shock, the difference between the value for hand-to-mouth consumers and that implied by the permanent income hypothesis being close to unity. By comparison, the MPCs for workers to an unanticipated shock, 0.24 in the first quarter and 0.68 cumulatively over the first year, lie squarely in the middle region between 0 and 1 – consistent with what data suggest for the behavior of the median household.

Before turning to the determinants of labor supply in the model, we briefly comment on the scope and limitations of our approach. First, the convex penalty function affords us tractability, and it generalizes the assumption of complete non-participation in asset markets by hand-to-mouth households; it clearly still is very stylized. The quadratic functional form is simple but very common in the literature and could be generalized without difficulty. That the cost is measured in deviations from a target level is consistent with an interpretation of households as “target savers,” that is, they have a target allocation for liquidity or long-term savings and are penalized if asset holdings deviate from that target. More broadly, the use of a financial friction constraining households’ consumption-savings choice is in line with extensive micro evidence for gradual portfolio adjustment.^16^ There is also a rich literature in finance and international macro showing how such frictions can help capture households’ investment behavior, and what underpins them.^17^

Second, in generating household consumption dynamics in response to income windfalls that are consistent with micro data, the model here bears similarity not only to multi-asset HANK models but also to other “tractable heterogeneous-agent” approaches.^18^ In Appendix A.2.1 we state conditions under which portfolio adjustment costs and the bond-in-utility specification are first-order equivalent, as well as providing a broader comparison. Finally, a limitation of the proposed tractable model is that it does not generate time-varying precautionary savings behavior due to uninsurable idiosyncratic risk – another key feature of HANK models. As such, our approach is complementary to literature contributions that provide tractable analyses of that property but do not focus on MPC heterogeneity, examples being Acharya and Dogra (2020), Challe (2020), or Ravn and Sterk (2020).

### 2.3 Labor supply and profit income effects

In the canonical TANK environment, the transmission of demand shocks is heavily influenced by shifts in the labor supply curve triggered by cyclical variations in markups due to price stickiness. This remains the case once limited asset market participation is generalized as proposed in the preceding section.^19^ This feature is implausible when it comes to the monetary transmission mechanism in the textbook representative-agent New Keynesian (RANK) model, as argued by Broer et al. (2020a); and it turns out to be quantitatively significant also in the propagation of fiscal policy measures according to the benchmark TANK model.^20^ We next make this property more precise and then adopt a simple amendment that addresses this issue.

Paralleling the approach taken by Broer et al. (2020a), combine the equations for labor supply, private and public budget constraints, and assuming no debt issuance. This yields an expression for total hours worked as a function of government spending and dividends:

(11) $${\hat{n}}_{t}=\frac{{\tilde{g}}_{t}-{\tilde{d}}_{t}}{1+\varphi }.$$

where ${\tilde{d}}_{t}=-{\hat{w}}_{t},$ and the wage is equivalent to labor’s share of income. It immediately follows that insofar as greater public demand for goods, ${\tilde{g}}_{t}>0,$ pushes down (up) profits (the labor share), the resulting income effect on labor supply *amplifies* the effects of fiscal measures.^21^ The countercyclical fall in profits is integral to the transmission of demand shocks in the New Keynesian model, resulting from an incomplete adjustment of product prices that leads to countercyclical movements in markups.

We share the view of Broer et al. (2020a) that this crucial role attributed to cyclical profit fluctuations in determining the effectiveness of macroeconomic policy is implausible as it appears in RANK. In the benchmark TANK model this mechanism is more forceful still. An initial exogenous increase in demand pushes up wages, raising hand-to-mouth households’ income, which they immediately and one-to-one use for consumption (as discussed in the preceding section). This pushes up wages further and beyond the level occurring under full participation. For production to meet this increased demand, hours worked must rise. Given preferences under which the direct substitution and income effects of wage changes cancel out, this occurs because of a negative income effect on labor supply arising from a fall in profits, which move inversely with wages. Following Bilbiie (2008), the distribution of profits is, thus, critical to the amplification of demand shocks in TANK models because of the very tight interdependence of labor and financial markets.

To eliminate this channel, we replace the usual unconstrained type of household with “capitalists” (indexed by C), who receive firm profits but do not supply labor. This approach extends to the TANK framework the idea articulated by Broer et al. (2020a) in their critique of the textbook RANK model. The amendment short-circuits the implausible profit income effect on labor supply. The only difference between the U and C types is that the latter are assumed not to participate in the labor market ($n_{t}^{C}=0$) and, consequently, labor income does not enter into their budget constraint.^22^ As such, cyclical variations in profits no longer determine the effectiveness of fiscal policy through their income effect on labor supply.^23^

## 3 Household heterogeneity and fiscal policy

What are the implications for the transmission of fiscal policy of these alternative ways of characterizing household heterogeneity within the two-agent New Keynesian (TANK) framework? To answer this question, we now compare the general equilibrium effects of a discretionary increase in government spending implied by calibrated versions of the different TANK models.

**Fig. 4:**
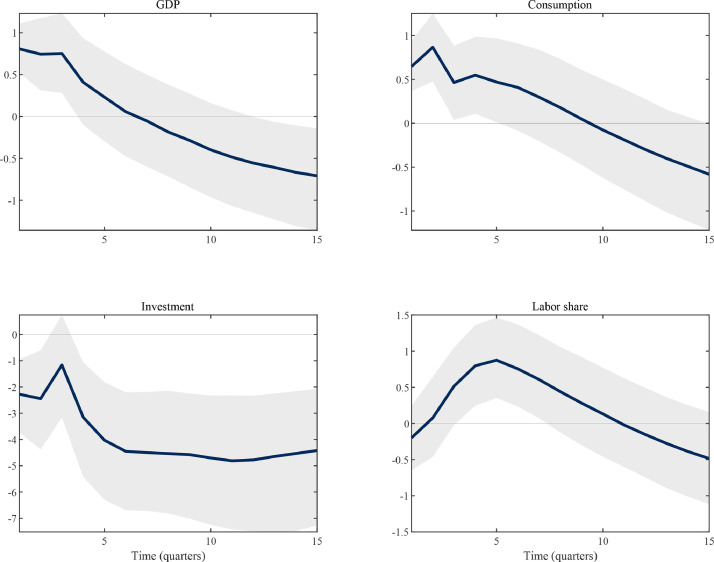
Empirical effects of an unanticipated shock to government spending (U.S.). *Notes:* The figure shows empirical impulse responses for an unanticipated government spending shock. Impulse responses are scaled such that the increase in government spending is equal to one percent of GDP. All series are shown in percent deviation from baseline. Solid lines indicate the median posterior density of impulse responses, while the shaded area represents the 16th to 84th percentiles.

To provide an empirical reference point, Fig. 4 indicates the effects of an unanticipated increase in government purchases in U.S. data, as recovered using the structural vector autoregression approach devised by Forni and Gambetti (2016).^24^ In brief, unanticipated expansionary government spending shocks are associated with positive but relatively short-lived output effects.^25^ They also shift the composition of private-sector spending from investment to consumption. Moreover, they induce a redistribution of income from recipients of profit income towards recipients of labor income; the impulse response of labor’s share of national income is positive, persistent and hump-shaped.

### 3.1 Parameterization

To facilitate maximum transparency, we continue using the very simple models outlined in Section 2.1 and summarized in Table 1. Table 2 summarizes their parameterization. The values are chosen to maintain comparability across models and stay close to the literature. We therefore limit discussion to two key parameters: the population shares of different types of households, λ, and $\psi^{W},$ which indexes portfolio adjustment costs faced by workers. Appendix B.2 provides, furthermore, extensive sensitivity results for alternative parameter combinations.

**Table 2:** Parameter values.

Parameter	Interpretation		Value (H | W)		Source
β	Discount factor		0.99		Annual real interest rate of 4%
$\rho^{G}$	AR1 Government spending shock		0.9		Benchmark
$\psi^{W}$	Portfolio adjustment cost		∞ | 0.07		Definition | iMPC evidence
λ	% of H/W		0.19 | 0.8		iMPC evidence
$b^{W}$	Workers’ steady-state bond holdings		0		Comparability of models
ξ	Rotemberg price stickiness		42.68		Average price duration 3.5q
$\phi^{\pi }$	Interest rate response to inflation		1.5		Galí et al. (2007)
$\phi^{\tau ,t}$	Tax smoothing		0		Galí et al. (2007)
$\phi^{\tau ,g}$	Tax response to government spending		0.1		Galí et al. (2007)
$\phi^{\tau ,b}$	Tax response to debt		0.33		Galí et al. (2007)
Π	Steady-state inflation rate		1		Benchmark
φ	Inverse Frisch elasticity		0.05		Determinacy of UH
η	Int. goods elasticity of substitution		6		Steady-state profits excl. subsidy
$\tau^{S}$	Production subsidy		${\left(\eta -1\right)}^{-1}$		Marginal cost pricing

*Notes:* This table lists the parameter values of the simple TANK models studied in Section 3.2. One period in the model corresponds to one quarter. Explanations for the acronyms: H – hand-to-mouth; W – worker; UH – unconstrained and hand-to-mouth households.

We calibrate these two parameters by targeting the evidence from micro data on household-level consumption responses to income windfalls described in Section 2.2. Specifically, we draw on the evidence from Fagereng et al. (2018) illustrated in Fig. 1(a). In models with portfolio adjustment costs (PACs), we target, firstly, a quarterly impact MPC equal to 0.1942 and, secondly, an annual MPC equal to 0.55. The quarterly value is interpolated but very close also to other estimates from the empirical literature.^26^ In terms of practical implementation, we exploit the analytical characterization of iMPCs described in Proposition 1 to find the values of λ and $\psi^{W}$ such that we exactly match these two moments from consumption micro data. This step involves solving a simple system of two simultaneous equations. It yields λ=0.8 and $\psi^{W}=0.07$. Reassuringly, estimating the model on *macro* time-series data leads to very similar parameter values (see Appendix C).^27^

In the benchmark UH model, the parameter $\psi^{W}$ is set to infinity by definition. Thus, we can only hit one of the targets. As the average partial equilibrium impact MPC in that model is simply λ×1+(1−λ)×(1−β), given $\beta =R^{-1}=0.99,$ matching a value of 0.1942 then requires λ≈0.19. This value is close to or slightly below typical estimates of the population share of hand-to-mouth consumers, which are between 20% (Slacalek et al. (2020)) to 30% (Kaplan et al. (2014)).^28^

### 3.2 Government spending shocks in simple TANK models

Transmission mechanisms. The alternative ways of characterizing household heterogeneity within the TANK framework described in the preceding section have consequences for the transmission of macroeconomic policy; in particular for labor supply and consumption dynamics. We explicate this point by considering the effects of government spending shocks according to the benchmark (UH) and our proposed alternative (CW) model. By also showing results for the intermediate model case that combines unconstrained households and workers (UW), we separately identify what the effects are, respectively, of partial access to capital markets for constrained households (as in UW and CW, but not in UH) and those of removing profit income effects on labor supply (as done in CW, however not in UH or UW).

**Fig. 5:**
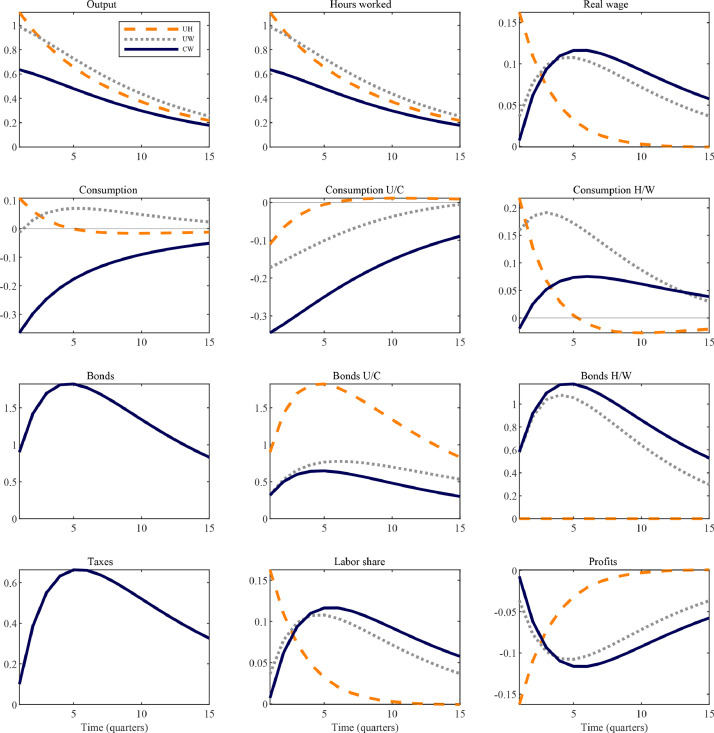
Government spending shocks in alternative TANK models. *Notes:* The figure shows the impulse responses of selected variables to a government spending shock equal to one percent of steady-state output in different, simple TANK models. All series are in percent deviations from their steady state except for the fiscal variables (government spending, bonds and taxes) and profits, which are measured in percentage of steady-state output. Consumption components are weighted by population shares. Explanations for the acronyms: UH – unconstrained and hand-to-mouth households; UW – unconstrained and worker households; CW – capitalist and worker households.

Fig. 5 shows the impulse responses of selected variables to an unanticipated, discretionary expansion in public demand equal to one percent of steady-state output. Consider first the commonalities across the models. The increase in government purchases raises the level of aggregate demand in the economy. Following the familiar New Keynesian narrative, firms operating under monopolistic competition raise their prices, however, given nominal rigidities this change is insufficient to fully restore the original equilibrium. The labor demand curve shifts outwards, hours worked and, hence, output rise; so does the real wage. The flipside of rising wages is a countercyclical fall in markups, due to the incomplete adjustment of product prices, causing a decline in profit income and a rise in labor’s share of income. In contrast to the representative-agent paradigm, in TANK models limited asset market participation by a fraction of households raises their MPCs above the low level implied by the permanent-income hypothesis. Accordingly, both hand-to-mouth (H) and worker (W) households use their now higher levels of disposable labor income to enjoy more consumption. In contrast, for unconstrained, equity-owning households (both U and C) the relatively less benign income dynamics associated with the aforementioned fall in profit income, as well as a relatively higher absorption of bonds, means that consumption falls. In line with the empirical evidence, thus, according to TANK models fiscal policy shocks have not only *aggregate* but also important *redistributive* effects that reveal themselves through both changes in the (functional) income distribution and heterogeneous consumption behavior.^29^

The first visible difference across models is that according to the CW model (solid line), hours worked and output rise substantially less than in the UH and UW models (dashed and dotted lines). According to the second row of panels in Fig. 5, furthermore, the overall decline in consumption is accounted for most notably by the substantial fall in capitalists’ purchases. These differences arise precisely because the CW model no longer features the expansion in labor supply due to the income effect induced by countercyclical profits that drives the rise in hours worked in the other two models – as discussed in Section 2.3. Since capitalists’ disposable income does not include the procyclical component of labor earnings, they are forced to strongly cut back on consumption. The combination of an increase in labor demand due to additional government expenditures combined with no labor supply response by capitalists implies that the labor share nonetheless rises robustly.

To identify the implications of allowing for partial rather than no access to capital markets by constrained households, compare the two TANK variants that differ in one respect only: that UH features hand-to-mouth households, while UW includes workers who have the ability to save in form of government bonds subject to adjustment costs of intermediate strength. Comparing dashed and dotted lines reveals that consumption by workers is high and positive on impact, just as for hand-to-mouth households, but also remains elevated for an extended time.

These results map onto the discussion of the partial equilibrium models in Section 2.2 in important ways. The slower rate of decline characterizing workers’ as compared to hand-to-mouth households’ consumption levels is consistent with the pattern of empirically realistic, gradually decaying iMPCs for the former but not the latter type of household. Moreover, because workers react to both current and expected future changes, the logic underlying their general equilibrium consumption response to fiscal shocks is also inherently dynamic in a way that does not hold true for hand-to-mouth consumers, but conforms to the logic of HANK models as described by (Hagedorn et al., 2019b). On the one hand, following an increase in disposable income today, the worker household saves a fraction to be spent in the future, such that not only current but also future consumption is increased. This elevated path of demand puts upward pressure on future wages and, hence, expected labor income and consumption. The anticipation of greater future earnings induce workers to consume even more today. At the same time, however, workers are responsive to the anticipated rise in future tax liabilities as well as a rise in the real interest rate, as the central bank seeks to stabilize inflation, depressing their consumption.^30^

Fiscal multipliers – size and path. We conclude this section by zooming in on two key implications for the magnitude and path of fiscal multipliers that the proposed TANK-CW model has relative to the traditional model. First, the CW model implies smaller fiscal multipliers than both the traditional UH model and the UW variant. Table 3 summarizes both impact and cumulative fiscal multipliers. The CW model implies that both measures of the stimulative effect on output lie below one – at 0.48 and 0.62, respectively –, whereas the values for UH and UW are similar and cluster just about unity. These differences in predictions for the size of fiscal multipliers follow immediately from the analysis in Section 2.3. The UH model, as well as the UW variant, crucially rely on income effects on labor supply induced by countercyclical markups to generate large output effects of public demand expansions. The model with capitalists shuts off this channel – which lacks empirical support, as argued by Broer et al. (2020a).^31^

**Table 3:** Fiscal multipliers according to simple and medium-scale models.

		Simple models					Medium-scale models			
		RA	UH	UW	CW		RA	UH	UW	CW
Impact multiplier		0.96	1.11	0.99	0.64		0.81	0.95	1.21	1.32
Cumulative multiplier		0.96	1.00	1.08	0.73		0.42	0.45	0.58	0.79

*Notes:* This table summarizes the output effects of a government spending shock according to different TANK models: the first main column refers to the simple models described in Table 1, the second refers to medium-scale variants (set out in detail in Appendix B.3). Explanations for the acronyms: RA – representative agent; UH – unconstrained and hand-to-mouth households; UW – unconstrained and worker households; CW – capitalist and worker households. In the simple models, where the steady-state of government spending is zero, the impact multiplier is computed as $dy_{0}/dg_{0}$ and the cumulative multiplier as $\sum_{l=0}^{\infty }\beta^{l}dy_{l}/dg_{l}$. In models with positive government spending in steady state, these objects are normalized accordingly.

Secondly, allowing for intermediately constrained households, rather than complete exclusion from capital markets, alters the sensitivity of the output path to the mix of taxes and deficits that finance the fiscal stimulus. To see why, note first that the combination of household heterogeneity and limited asset market participation generally gives rise to potentially significant feedback effects to deficit-financing from the private sector (e.g., Bilbiie et al. (2013)). The reason is that under deficit-financing, the offsetting effect through higher taxes is more limited and, hence, constrained agents’ post-tax disposable income is higher. Given their relatively high propensity to consume, goods demand increases in a sustained manner. On the other hand, unconstrained and capitalist households act in a Ricardian fashion and, therefore, when the government alters the balance between deficit- and tax-finance this has no direct impact on their consumption choices.^32^

**Fig. 6:**
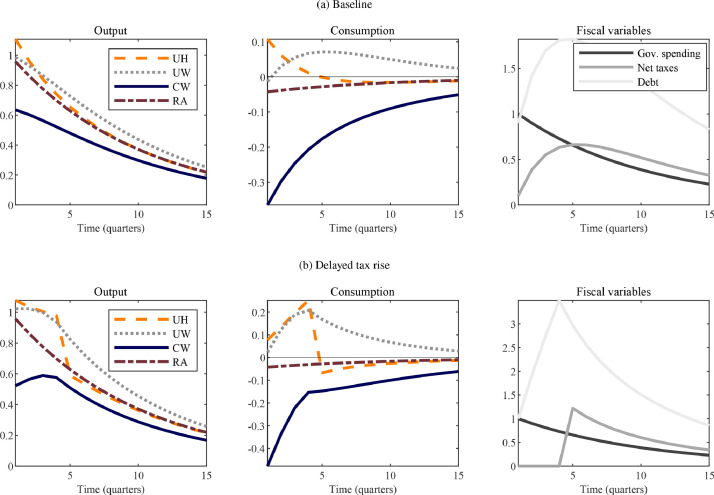
Fiscal stimulus effects in simple models. *Notes:* The figure shows the impulse responses of selected variables to a government spending shock equal to one percent of steady-state output in different, simple TANK models. The lower panel imposes that any increase in net taxes is delayed by four quarters. The analysis is implemented using perfect foresight simulations. Output and consumption are in percent deviations from their steady-state and the fiscal variables are measured in percentage of steady-state output. Explanations for the acronyms: RA – representative agent; UH – unconstrained and hand-to-mouth households; UW – unconstrained and worker households; CW – capitalist and worker households.

Empirically realistic iMPCs render these feedback effects less extreme than in the model where hand-to-mouth consumers do not participate in financial markets at all. Fig. 6 contrasts the benchmark case in which the tax/debt mix is determined according to the standard tax rule (Panel 6(a)) with an alternative fiscal path that *postpones* any increase in taxes by four quarters (Panel 6(b)). The latter scenario captures, for instance, a government aiming to stimulate the economy in a recession without wanting to immediately reduce private sector incomes. As the dashed line shows, in the UH model, the contemporaneous output multiplier is extremely sensitive to the public financing mix. It remains elevated as long as the stimulus is purely deficit-financed but declines sharply down after fourth quarters, closely tracking the rise in taxes. This relationship is due to the extreme MPCs of hand-to-mouth households whose consumption spending tracks net disposable income one-for-one. Indeed, Auclert et al. (2018, Proposition 5) show in a special case of the UH model that even though this version of the two-agent framework generates potentially large impact multipliers for a spending policy that is entirely deficit-financed, it nonetheless implies unitary cumulative multipliers, the reason being precisely that hand-to-mouth households’ consumption declines as soon as deficits are turned into surpluses. In this sense, the TANK-UH model has implications similar to those of the representative-agent model, in which multipliers are generically around unity, irrespective of the financing mix. In models with intermediately constrained worker households, too, the output path is related to the mix of debt and taxes financing the expansion in public spending. Comparing the dotted lines across the panels shows that delaying the tax hike does raise aggregate consumption, as households’ disposable income is higher. However, the variation induced in the consumption response by alternative financing schemes is much smoother than in the traditional model with hand-to-mouth households. The reason is that even though workers are not fully Ricardian and, hence, the path of debt and taxes *does* affect their consumption levels, they *partially* anticipate the implications of higher future taxes and smooth consumption to at least some extent. The experiment reveals, thus, that matching empirically plausible iMPCs at the micro level matters for what model-based counterfactuals suggest are the effects of alternative fiscal stimulus packages at the macro level.

### 3.3 Extensions and medium-scale models

The theoretical environment considered thus far was purposefully simple, allowing us to characterize the implications of the proposed amendments to the TANK benchmark model in a transparent manner. Here we briefly consider medium-scale variants that incorporate key ingredients typically found in quantitatively-oriented DSGE models. The most important ones are nominal wage stickiness and physical capital accumulation subject to investment adjustment costs. In addition, there are fixed costs in production, capital utilization is variable, the Taylor rule features interest rate smoothing, and government spending as well as debt are positive in steady state. As these features are standard, we relegate all details to Appendix B.3.

Two main results merit comments. First, cumulative output multipliers are more similar across the different medium-scale TANK variants, as the second main column of Table 3 documents, and the previous ranking no longer holds. The primary reason for this result is directly related to the analysis of transmission mechanisms in the traditional and proposed new TANK models offered in Sections 2.3 and 3.2. Large multipliers in the benchmark UH model – as well as the UW variant – arise because of income effects on labor supply due to countercyclical profits. This channel is largely suppressed with even moderate degrees of wage stickiness, as profits move less countercyclically (also see Broer et al. (2020b)). On the other hand, the model with capitalists was constructed so as to remove that channel in the first place and its predictions are, hence, less sensitive to the introduction of wage stickiness.

**Fig. 7:**
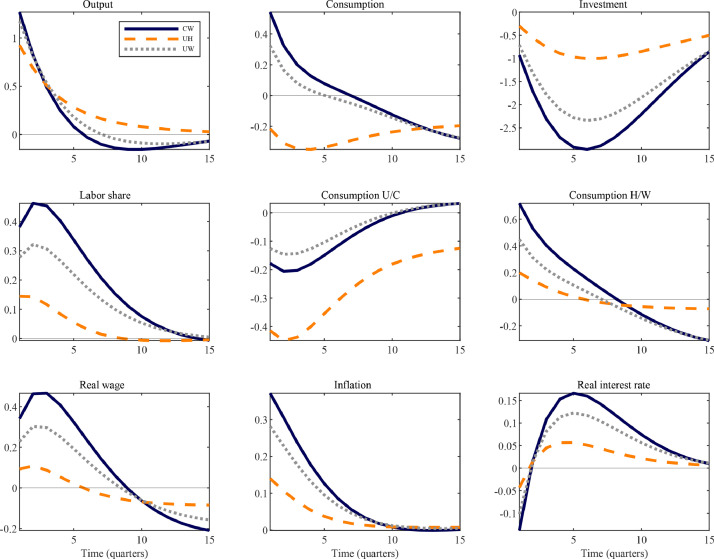
Government spending shocks in medium-scale TANK models. *Notes:* The figure shows the impulse responses of selected variables to a one percent increase in government spending according to different, medium-scale TANK models. All series are in percent deviations from their steady-state. Consumption components are weighted by population shares. Explanations for the acronyms: UH – unconstrained and hand-to-mouth households; UW – unconstrained and worker households; CW – capitalist and worker households.

Second, Fig. 7 illustrates that the extended TANK-CW model implies a positive but short-lived response of aggregate consumption to a deficit-financed increase in public spending, while investment is crowded out. Both these moments are in line with the empirical evidence summarized in Fig. 4. Endogenous capital accumulation plays an important role in altering consumption dynamics. In particular, capitalists’ consumption falls by less. For a given net income loss, they can now use investment for consumption smoothing purposes; and their budget position is less negatively affected in the first place, as capital income, unlike dividends, is procyclical, as emphasized by Bilbiie et al. (2020). Moreover, (empirically plausible) interest rate smoothing limits the extent to which higher real rates incentivize both capitalist and worker households to raise savings.

## 4 Conclusion

This paper introduced a two-agent New Keynesian (TANK) model with capitalists and workers that matches the implications of richer heterogeneous-agent (HANK) models in key dimensions, while remaining tractable. At the micro level, the model generates a realistic pattern of intertemporal marginal propensities to consume, in line with the evidence in Auclert et al. (2018) and the implications of multi-asset HANK models. Moreover, the model is immune to the Broer et al. (2020a) critique, in that the transmission of demand shocks under sticky prices does not rely on income effects on labor supply induced by countercyclical profits. These features have implications for the macro effects of deficit-financed expansions of government spending. Relative to the predictions of the traditional two-agent model with flexible wages, fiscal multipliers are smaller; and the (otherwise extreme) sensitivity of the output path to public deficits is dampened. The bigger picture of how household inequality affects the aggregate impact of fiscal policy on economic activity is, then, as follows. Household heterogeneity is crucial in shaping the private sector’s demand response to shocks through variation in consumption propensities, yet cyclical income redistribution between capitalists and workers does not mechanically shift labor supply.

The model developed here can serve as a tractable framework to study the interaction of macroeconomic policy and household heterogeneity. The role envisioned is distinct from that of full-blown heterogeneous-agent models; indeed, certain questions are not well-defined in the present setting but can be fruitfully explored in HANK models.^33^ TANK models are, however, potentially useful as tractable laboratories for understanding various macroeconomic experiments. As they are fast to solve and estimate, they also lend themselves to quantitative applications incorporating a wide range of empirically relevant frictions. Relevant questions to consider in future applications of the proposed model concern, for instance, the study of monetary policy (e.g., forward guidance). A promising avenue for future research is, furthermore, to combine our approach to household heterogeneity with tractable models of precautionary savings and cyclical, uninsurable risk.

## Appendix A Supplementary materials

Supplementary Data S1 Supplementary Raw Research Data. This is open data under the CC BY license http://creativecommons.org/licenses/by/4.0/ Supplementary Data S1

Supplementary Data S2 Supplementary Raw Research Data. This is open data under the CC BY license http://creativecommons.org/licenses/by/4.0/ Supplementary Data S2

## Footnotes

1 The matrix of iMPCs is defined as the Jacobian with typical element $\partial c_{t}/\partial x_{s},$ interpreted as the response of consumption at date t to an income shock at date s.

2 In terms of theoretical alternatives to the two-agent approach, Werning (2015), McKay and Reis (2016), Ravn and Sterk (2020) and Challe (2020) use a zero-liquidity approach, while Acharya and Dogra (2020) exploit convenient properties of CARA preferences.

3 Among the many other recent, related contributions to the TANK literature are Sims and Wu (2019), Bilbiie (2019a) and Bilbiie et al. (2020).

4 To aid with orientation, the colored version of this paper uses (shades of) orange for the traditional two-agent setup and (shades of) blue for the capitalist-worker model throughout the entire paper.

5 This specification, used by Debortoli and Galí (2018) among others, keeps the supply side simple. It also allows for a straightforward introduction of wage stickiness. All the key results from this section are unaffected if different household types choose hours separately, instead. In our proposed capitalist-worker model the distinction dissolves.

6 A third issue is that the benchmark model’s rational expectations solution is prone to indeterminacy outside a fairly narrow region of the parameter space. A corollary benefit of the proposed introduction of portfolio adjustment costs as described in the following is that the resulting model also has more plausible stability properties, in that it does not require an implausible value of the Frisch elasticity for determinacy, and remains determinate under an active Taylor rule for all plausible values of the population share of constrained households. We relegate a detailed discussion to Appendix.

7 Wolf (2020) extends this notion of (demand) equivalence (in terms of general equilibrium propagation) to a larger family of models, and finds support for approximate equivalence in micro and macro data. Hagedorn et al. (2019b) similarly underscore the importance of matching observed marginal propensities to consume in order to quantitatively evaluate the dynamic consumption responses to stimulus policies, using a heterogeneous-agent model with incomplete markets, physical capital and rigidities in both prices and wages.

8 This approach generates individual iMPCs, which appear to be the natural analogue to empirical estimates of household spending responses to sudden *individual* income changes such as the winnings of lotteries studied by Fagereng et al. (2018)). The aggregate iMPC matrix M is then a convex combination of individual iMPCs (see Auclert et al. (2018, Lemma 3)), taking account of the responsiveness of private incomes to aggregate income. Bilbiie (2019b) underscores and carefully dissects the role of the income elasticity of hand-to-mouth household with respect to aggregate income in determining the properties of the TANK model. More generally, the aggregate MPC (at time t) out of an increase in aggregate income (in period s) is $M_{t,s}=\frac{\partial c_{t}}{\partial x_{s}}=\int_{i}\frac{\partial c_{t}^{i}}{\partial x_{s}}di=\int_{i}\frac{\partial c_{t}^{i}}{\partial x_{s}^{i}}\frac{\partial x_{s}^{i}}{\partial x_{s}}di$.

9 The theoretical iMPCs displayed in Figs. 2 and 3 abstract from endogenous adjustments in household earnings due to, most significantly, income effects on labor supply. Doing so allows us to squarely focus on the direct implications of portfolio adjustment costs, as introduced below (see Wolf (2020, footnote 7) for additional reasons to adopt this approach). We address income effects on labor supply separately below.

10 Though time variation in the real return or inflation play no role in this section, we allow for them to ensure consistency with the general equilibrium approach taken in the rest of the paper.

11 The extension to allow for a non-unitary elasticity of intertemporal substitution is straightforward.

12 We use the terminology of “portfolio adjustment costs” following the influential work of Schmitt-Grohe and Uribe (2003) who use that expression to refer to the convex costs of holding assets in quantities different from some long-run level. An alternative term is “bond holding costs,” as in Neumeyer and Perri (2005).

13 As such, the analysis and results in Propositions 1 and 2 are strictly valid only if $b^{W}$ is stationary, which is the case when ψ>0 (cf. Schmitt-Grohe and Uribe (2003)).

14 Appendix A.3 formally validates the claims in this paragraph analytically and graphically.

15 Debortoli and Galí (2018) stress the importance of variations in the consumption gap between constrained and unconstrained households for aggregate dynamics.

16 For a review of the literature, see Chien et al. (2012, Section II.). Data from the 2016 Survey of Consumer Finances are, furtheremore, at least suggestive: Fewer than 6 percent of U.S. households adjust their portfolio of stocks and other securities through a broker on a monthly basis; over an annual horizon this figure rises to 68 percent.

17 See, for instance, Bacchetta and van Wincoop (2010) and Gârleanu and Pedersen (2016).

18 See Section 1 for literature references.

19 As such, this class of models also does not satisfy the conditions under which iMPCs are sufficient statistics for the general equilibrium propagation of demand shocks (Auclert et al. (2018)), nor the related assumption of “demand equivalence” by which changes in private consumption demand are accommodated in general equilibrium exactly like changes in aggregate public spending (Wolf (2020)). Our objective here is not to construct a TANK variant satisfying these conditions, but we note that in Section 3.3 we consider the case of largely demand-determined aggregate hours worked, which reduces the distance to that theoretical benchmark further.

20 Alves et al. (2020) stress that the distribution of profits plays just as critical a role in HANK settings.

21 Furthermore, monetary policy that does not induce a fiscal response (i.e., ${\tilde{g}}_{t}=0$) would have *no* effects on hours worked and, hence, output, were it not for the fact that cyclical variations in profits, as a form of non-labor income, trigger changes in households’ demand for leisure. This result is the TANK-counterpart to (Broer et al., 2020a) analysis of the textbook-RANK monetary transmission mechanism.

22 Of course, this is unrealistic insofar as even the majority of the wealthiest ten percent of individuals’s income in the U.S., for instance, derives from labor, according to the Survey of Consumer Finances. From a modeling perspective, though, the goal is to eliminate the income effects on labor supply of variations in markups. Our “capitalist” specification accomplishes this goal in a stylized form. An alternative approach that gives rise to virtually identical dynamics out of steady state but is analytically slightly less convenient assumes that the capitalist-type does supply labor, but inelastically.

23 Appendix C shows that introducing capitalists into the TANK framework is feasible without giving up on equilibrium uniqueness under an active Taylor rule only in the presence of non-zero portfolio adjustment costs. In this way, the two innovations to the TANK setup that we propose are complementary.

24 Appendix D provides details on data and implementation of the VAR as well as a more extensive set of results.

25 The impact multiplier is 0.8, but the discounted cumulative multiplier after two years is only 0.48, reflecting the short-lived nature of the expansion.

26 For instance, Kaplan and Violante (2014) describe 25 percent as a reasonable approximation of the fraction of fiscal stimulus payments households spend on nondurable consumption in the quarter that they are received. Kueng (2018), who studies data from the Alaska Permanent Fund, likewise finds that the average marginal propensity to consume is 25% for nondurables and services within one quarter of the payments. Hagedorn et al. (2019b) cite the middle range of annual impact iMPCs out of transitory income as 0.4, while the median year-1 iMPC in the Italian Survey of Household Income and Wealth it is equal to 0.45, according to Auclert et al. (2018). If the simple relationship $M_{0,0}^{a}=1-{\left(1-M_{0,0}^{q}\right)}^{4}$ is used to convert between annual and quarterly figures, the latter data points imply quarterly impact values slightly below 0.2. Appendix B.2 shows that alternative calibration approaches yield highly comparable results.

27 This finding is consistent with the results in Wolf (2020) that point toward a near-zero general equilibrium intercept.

28 Notice, finally, that we set 1/φ equal to 20 across all models. Using a more conventional value like φ=1 leads to two problems in the benchmark TANK setup. Firstly, the model with λ=0.1919 no longer delivers the crowding-in of private consumption that it was partly designed to generate (cf. Galí et al. (2007)). Secondly, in Appendix B.2 we conduct an additional comparison of TANK models that constrains the calibration to have half of the population with unconstrained access to financial markets across all models. But if the share of hand-to-mouth households is one half, then the benchmark model requires a high Frisch elasticity of labor supply to avoid indeterminacy (see Appendix C). Using φ=0.05 allows us to obtain consumption crowding-in with the UH model in the baseline case, and it facilitates model comparison even when λ=0.5.

29 Complementing the evidence on the positive labor share response to government spending shocks in Fig. 4, Ma (2019) documents heterogeneity in the consumption responses of poor and rich households.

30 Notice, furthermore, that the path of workers’ consumption is lightly hump-shaped, a pattern that is inherited also by aggregate consumption; and since the latter influences labor demand, also by the labor share. This hump shape is contingent upon the particular fiscal rule we consider rather than being a direct consequence of workers’ iMPCs; the latter peak on impact. To be more precise, the presence of portfolio adjustment costs generates a hump-shaped path for workers’ consumption provided the path for the supply of liquidity in form of government bonds is hump-shaped. Intuitively, the greater the excess of bonds issued by the government to finance its additional spending relative to workers’ target level, the greater is workers’ desire to consume so as to avoid a penalty in the form of adjustment costs. See Appendix B.2 for graphical illustrations.

31 In very recent work, Broer et al. (2020b) follow up on their earlier paper and come to very similar conclusions as we do. Their focus is on the textbook representative-agent model; in that way the papers are complementary.

32 There is, however, an important indirect effect based on implicit income redistribution (cf. Hagedorn et al. (2019b)). For when aggregate demand is higher due to deficit-financing, wages are pushed up whereas profits are compressed – benefiting workers but reducing firm owners’ profit income.

33 One example is the varied impact of shocks across the entire income distribution (e.g., Bayer et al. (2020)).
