# Supplementary material for: Workers, capitalists, and the government: fiscal policy and income (re)distribution
Source: J Monet Econ. 2021 Apr;119:58–74. doi: 10.1016/j.jmoneco.2021.01.004 (PMC8100925; doi:10.1016/j.jmoneco.2021.01.004)
Supplement: Supplementary Data S1 — Supplementary Raw Research Data. This is open data under the CC BY license http://creativecommons.org/licenses/by/4.0/ [file mmc1.pdf]

Online Appendix for:

“Workers, Capitalists, and the Government: Fiscal Policy and Income (Re)Distribution”

Cristiano Cantore[†] Lukas B. Freund[‡]

January 2021

This online appendix contains supplemental material for the article “Workers, Capitalists, and the Government: Fiscal Policy and Income (Re)Distribution.”

Any references to equations, figures, tables or sections that are not preceded by a capital letter refer to the main article.

Contents

Appendices **1**

A Partial equilibrium consumption models **3**

A.1 Proofs of Propositions 1 and 2 3

A.2 Comparison to alternative frameworks 4

A.2.1 Bonds in utility 4

A.2.2 Habits 7

A.3 Interest rate effects and discounting in the Euler equation 8

B TANK models **11**

B.1 Simple TANK models 11

B.1.1 Equilibrium conditions 11

B.2 Sensitivity results 13

[†]Bank of England, CfM & University of Surrey. Email: cristiano.cantore@gmail.com. Web: cristianocantore.com.
[‡]University of Cambridge. Email: lukas.beat.freund@gmail.com. Web: lukasfreund.com. Corresponding author.

B.2.1 Calibration with equal population shares 13

B.2.2 Variations in λ 16

B.2.3 Variations in ψ^W 20

B.2.4 Variations in fiscal rule parameters 23

B.3 Medium-scale models 25

C Determinacy in TANK models **29**

D Empirics **34**

D.1 Baseline specification and results 34

D.2 Results for Australia, Canada, and the UK 37

D.3 Further robustness checks 39

D.4 Data Sources and Transformation 39

E Estimation through IRF matching **42**

Appendix A Partial equilibrium consumption models

A.1 Proofs of Propositions 1 and 2

To solve the partial equilibrium consumption-savings problem with portfolio adjustment costs $\psi^W > 0$, first substitute out consumption from the worker household's Euler equation (3) using the budget constraint (4):

$$\left(1 + \beta^{-1} + \psi^W\right) \tilde{b}_t^W = E_t \left[\tilde{b}_{t+1}\right] + \beta^{-1} \tilde{b}_{t-1}^W + E_t \left[(\hat{x}_t^W - \hat{x}_{t+1}^W) + \hat{r}_t\right]. \tag{A.1}$$

This forward-backward system can be solved using various methods, such as factorization or the method of undetermined coefficients. To apply the former, and denoting the forward operator F, rewrite equation (A.1) as

$$\left(F^2 - (1 + \beta^{-1} + \psi^W)F + \beta^{-1}\right) E_t \tilde{b}_{t-1}^W = -E_t \underbrace{[(\hat{x}_t^W - \hat{x}_{t+1}^W) + \hat{r}_t]}_{\equiv \hat{z}_t^W}. \tag{A.2}$$

We can factorize the quadratic equation as $(F - \mu_1)(F - \mu_2)$, where, by Vieta's rule, the roots of the characteristic polynomial satisfy $\mu_1 + \mu_2 = 1 + \beta^{-1} + \psi^W$ and $\mu_1 + \mu_2 = \beta^{-1}$. Given solutions $\mu = \frac{1}{2}\left(1 + \beta^{-1} + \psi^W \pm \sqrt{(1 + \beta^{-1} + \psi)^2 - \beta^{-1}}\right)$, we focus on the saddle-path stable configuration. For $\psi^W > 0$, we have $\mu_1 = \frac{1}{2}\left(1 + \beta^{-1} + \psi^W - \sqrt{(1 + \beta^{-1} + \psi^W)^2 - \beta^{-1}}\right) < 1$ for the backward-looking part and $\mu_2 = \left(1 + \beta^{-1} + \psi^W\right) - \mu_1 > 1$ for the forward-looking part of the solution. Notice that for $\psi = 0$, the linearization around a steady-state $b^W = 0$ would not be valid, as assets are non-stationary, with $\mu_1 = 1$.

Next, rewrite equation (A.2) as

$$\Big((F-\mu_1)\times(F-\mu_2)\Big)E_t\tilde{b}^W_{t-1} = -E_t\hat{z}^W_t, \tag{A.3}$$

$$\Leftrightarrow \tilde{b}^W_t = \mu_1\tilde{b}^W_{t-1} - (F-\mu_2)^{-1}E_t\hat{z}^W_t, \tag{A.4}$$

$$= \mu_1\tilde{b}^W_{t-1} + \frac{\mu_2^{-1}}{1-F\mu_2^{-1}}E_t\hat{z}^W_t. \tag{A.5}$$

Since $|\mu_2| > 1$, we know that $(1 - F\mu_2^{-1}) = \sum_{l=0}^{\infty}\mu_2^{-l}F^l$. Consequently, the stationary solution is

$$\tilde{b}_t = \mu_1\tilde{b}^W_{t-1} + \sum_{l=0}^{\infty}\mu_2^{-(l+1)}E_t\left[(\hat{x}^W_{t+l} - \hat{x}^W_{t+l+1}) + \hat{r}_{t+l}\right]. \tag{A.6}$$

Consumption can be backed out from

$$\hat{c}^W_t = \hat{x}_t + \beta^{-1}\tilde{b}^W_{t-1} - \tilde{b}^W_t. \tag{A.7}$$

To compute iMPCs, we set $E_t[\hat{r}_{t+l}] = 0$ for all t and l. Equations (6) through (9) then follow by by differentiating $\hat{c}^W_t$ with respect to $\hat{x}_0$ (Proposition 1) and $E_0[\hat{x}_s]$, $s \geq 0$ (Proposition 2), respectively. Since the log-linearization is done around a steady-state with $x^W = 1$, the results correspond to iMPCs computed from a linearized version of the model.[A.1]

A.2 Comparison to alternative frameworks

A.2.1 Bonds in utility

Section 2.2 of the main text showed, using a simple partial equilibrium consumption-savings setup, that the introduction of bond portfolio adjustment costs (PACs) into the budget constraint, as in Schmitt-Grohe and Uribe (2003) and Neumeyer and Perri (2005), can give rise to intertemporal marginal propensities to consume (iMPCs) that are consistent with empirical data and match the

[A.1]Log-linearizing the model, albeit perhaps less common in this context than plain linearization, is convenient as it ensures consistency with the remainder of the paper.

predictions of multi-asset, heterogeneous-agent models. Dropping indices for the household type to ease notation, the relevant Euler equation could be written in the more general form

$$u'(c_t) + u'(c_t)\rho'(b_t) = \beta E_t u'(c_{t+1})(1 + r_t). \tag{A.8}$$

where $\rho'(\cdot)$ is the first derivative of a convex function. Impose that $\rho(b_t) = \frac{\psi}{2x}(b_t - b)^2$ and that $u(c_t) = \log(c_t)$. Then log-linearizing around a steady state with consumption normalized to unity, $1 + r = \beta^{-1}$ and zero net assets (as in the steady state of the general equilibrium version of the model), we arrive at the log-linearized Euler equation (3):

$$\hat{c}_t - \psi \tilde{b}_t = E_t \hat{c}_{t+1} - \hat{r}_t. \tag{A.9}$$

An alternative specification relies on the introduction of bonds into the utility function (BU) and can, under suitable functional form assumptions, be shown to be first-order equivalent, so that once linearized it also implies equation (A.9). In particular, suppose we set $\psi = 0$ but introduce an additional bond term into the household's objective function: $E_0 \sum_{t=0}^{\infty} \beta^t \left[u(c_t) + v(b_t)\right]$. Then the implied Euler equation is

$$u'(c_t) - v'(b_t) = \beta E_t u'(c_{t+1})(1 + r_t). \tag{A.10}$$

Thus, in general, equivalence between the two approaches requires that $v'(b_t) = -u'(c_t)\rho'(b_t)$, where the multiplicative term in the case of PACs arises because the costs are measured in financial rather than utility terms. Even thus, supposing that $v(b_t) = -\frac{\psi}{2x}(b_t - b)^2$ and linearizing around the same steady state, the multiplicative term disappears and, therefore, the PAC and BU approaches deliver *exactly* the same predictions up to first order.

This analysis demonstrates, thus, that there are multiple, equivalent, tractable ways of modeling household behavior consistent with the micro data on intertemporal marginal propensities to consume reviewed in Section 2.2. Intuitively, it is not surprising that the introduction of a convex cost to

savings and a concave benefit, respectively, can give rise to similar behavior. Both specifications imply that the marginal net benefit from saving is decreasing. Consequently, the household wants to consume more out of an income windfall than if $\psi = 0$, and the pattern of iMPCs displays a gradual decay as the household dis-saves. As described in the main text, the particular functional form assumption adopted here can be seen as formalizing the idea of households as exhibiting "target saving" behavior, that is, they aim to return, in the absence of shocks, to some long-run level, and are penalized the farther away from that target they move in an attempt to smooth consumption when hit by transitory shocks. The equivalence result here clarifies that this penalty could take either a financial or a psychological form.

We conclude with two practical observations. First, both the PAC‘and the BU specifications can be introduced into a two-agent framework in different ways. For instance, Auclert *et al.* (2018) propose‘a two-agent model that combines one type of agent with bonds in the utility with a fraction of hand-to-mouth agents, thus modifying the consumption behavior of the unconstrained households. By contrast, in our model we primarily tackled the consumption behavior of the constrained type of household. We adopted the latter approach in an effort to generalize the notion of "limited asset market participation" that is often viewed as underpinning hand-to-mouth consumption as incorporated into the traditional two-agent models of Campbell and Mankiw (1989), Galí *et al.* (2007) and Bilbiie (2008).

Second, it is quite common (e.g., Hagedorn (2018); Michaillat and Saez (2019) to model bonds in utility using a different functional form assumption that generates behavior akin to the precautionary savings motive characteristic of incomplete markets models, where that motive arises from a potentially binding borrowing constraint. Specifically, if there is non-zero demand for bonds in steady state, as would be the case if $v(\cdot)$ is is strictly positive, the equilibrium gross interest rate falls below β^{-1}. By contrast, as is natural in the context of "adjustment costs," we chose a functional form such that these costs are zero in steady state.[A.2] This approach lends itself to the straightforward analysis of dynamics away from the steady state.

[A.2] Of course, allowing the target savings level to be positive would make the two approaches more similar again.

(a) Habits (b) Portfolio Adjustment Costs

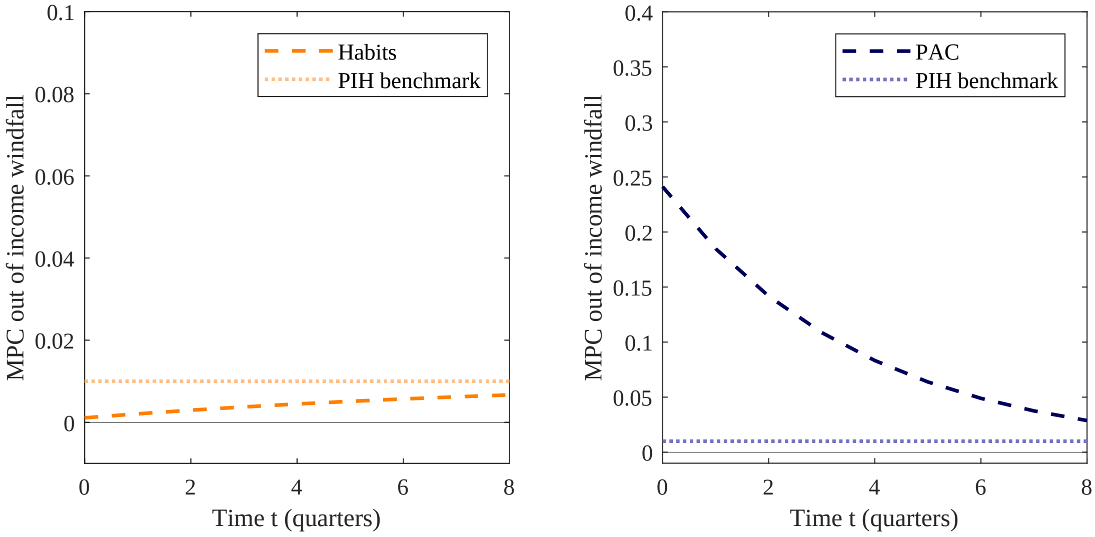

Figure A.1: Theoretical iMPCs: Comparison to Habits Model

Notes: The two panels show the dynamic consumption response to a period $s = 0$ unanticipated income shock under alternative specifications of the consumption/savings problem: the benchmark permanent income hypothesis (PIH), which corresponds to the "unconstrained" household in Figure 2 in the main text; an economy with habit formation (with $\xi = 0.9$); and a household subject to portfolio adjustment costs (PACs), denoted "worker" in the main text. The horizontal axis shows time measured in quarters. The vertical axis displays the marginal propensity to consume $\partial c_t / \partial x_0$.

A.2.2 Habits

The paper proposes limited asset market participation modeled through convex portfolio adjustment costs as a tractable way of describing household consumption behavior in line with both micro and macro data. This approach contrasts with the extensively used assumption of 'habit formation' in the utility function, which we briefly consider here.[A.3]

According to a common model of habit formation, the period utility function of a representative household i becomes $u(c_t^i) = \frac{1}{1-\sigma}\left(c_t(i) - \xi c_t\right)^{1-\sigma}$, where c_t is aggregate consumption, σ is the coefficient of relative risk aversion, and $\xi \in (0, 1)$ is a habit formation coefficient. As is well known, the resulting model can give rise to the kind of persistent and hump-shaped response of aggregate consumption to shocks commonly found in empirical macro studies.

Habit formation implies consumption dynamics that are inconsistent with the *micro* evidence,

[A.3] We consider external habit formation as in Smets and Wouters (2007). Internal habits give rise to similar properties.

however, as discussed in greater detail by Auclert *et al.* (2020a) (also see Carroll *et al.* (2018)). Figure A.1 illustrates. The left panel compares intertemporal marginal propensities to consume (iMPCs) as implied by the permanent income hypothesis (PIH) with those of a household with habits; the right panel reproduces relevant parts of Fig. 2a for convenience. It is evident that, at least for the first few quarters, habits imply *lower* MPCs compared to a no-habit economy ($\xi = 0$), thus exacerbating one of the key empirical challenges that the introduction of such frictions as limited asset market participation into theoretical models is intended to address. Second, habits imply *increasing* rather than decreasing first differences for the dynamic consumption response to an unanticipated income shock, making the model with habits look even more at odds with the micro evidence reviewed in Section 2.1 than the standard PIH.[A.4]

A.3 Interest rate effects and discounting in the Euler equation

In Section 2.2 we remarked that according to the partial equilibrium consumption model with portfolio adjustment costs, the household's response to interest rate changes is muted compared to unconstrained households. Moreover, the effect on current consumption of news about future real interest rates is decreasing in the horizon of the announcement. This contrasts with the prediction of the standard Euler equation, where such news have the same effect on current consumption as an equally large change to the contemporaneous interest rate: solving the Euler equation of an unconstrained household, i.e., when $\psi^W = 0$, we obtain $\hat{c}_t^U = \sum_{l=0}^{\infty} E_t \hat{r}_{t+l}$. Hence, for any s, $|\frac{d\hat{c}_t}{d\hat{r}_{t+s}}| = 1$.

Here we formally validate these claims. Paralleling Propositions 1 and 2, one can derive an analytical solution for the effect of interest rate changes on consumption given $\psi^W > 0$. In the absence of income shocks, the following holds to first order.

Proposition A.1 (Interest rate effects)**.** *The response of consumption when news arrives at $t = 0$ of*

[A.4]Havranek *et al.*'s (2017) survey of the micro literature furthermore finds little evidence for the existence of consumption habits sufficiently strong to explain the persistence or 'excess smoothness' of aggregate consumption.

a one-off change in the real interest rate $s \geq 0$ periods later is

$$\frac{d\hat{c}_0^W}{E_0\left[d\hat{r}_s\right]} = -\mu_2^{-(s+1)} \tag{A.11}$$

The subsequent expected path of consumption, for $t \geq 1$ obeys

$$\frac{E_0\left[d\hat{c}_t^W\right]}{E_0\left[d\hat{r}_s\right]} = \begin{cases} -\mu_2^{t-(s+1)} + (\beta^{-1} - \mu_1)\mu_1^{t-1}\mu_2^{-s}\sum_{l=1}^{t}\left(\frac{\mu_1}{\mu_2}\right)^{1-l}, & \textit{for } t \leq s \\ \mu_1^{t-(s+1)}(\beta^{-1} - \mu_1)\mu_2^{-1}\sum_{l=1}^{s}\left(\frac{\mu_1}{\mu_2}\right)^{l}, & \textit{for } t > s. \end{cases} \tag{A.12}$$

Figure A.2 illustrates the two properties. As the left panel indicates, whereas the standard model (with log utility) predicts consumption to respond one-for-one after a one-off interest rate cut, the effect is smaller when adjustment costs are positive ($\psi^W > 0$). In the following period, consumption mildly declines, in fact, as the household replenishes her savings. Furthermore, a worker household discounts news about future interest rate changes, that is, consumption responds less for greater values of s. This is illustrated in the right-hand panel A.2d.

(a) Effect on consumption of an interest rate cut in the current period

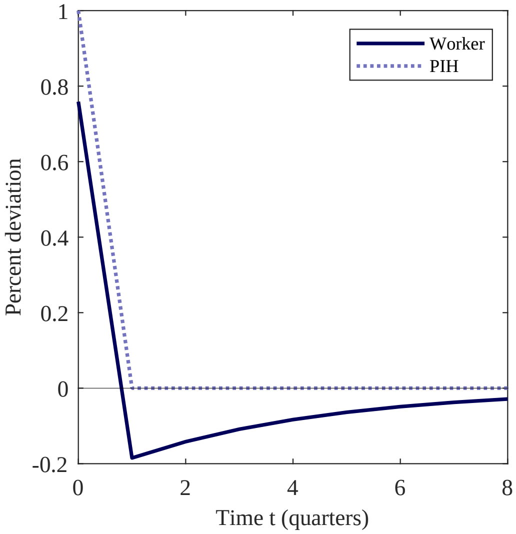

(b) Interest rate elasticity of consumption for different values of ψ^W

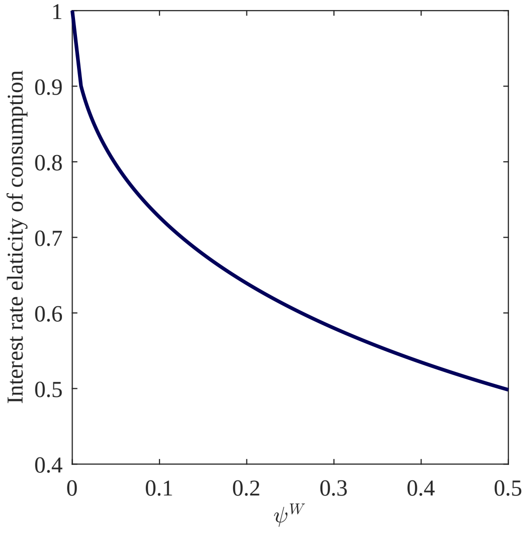

(c) Effect on consumption of news about an interest rate cut three quarters ahead

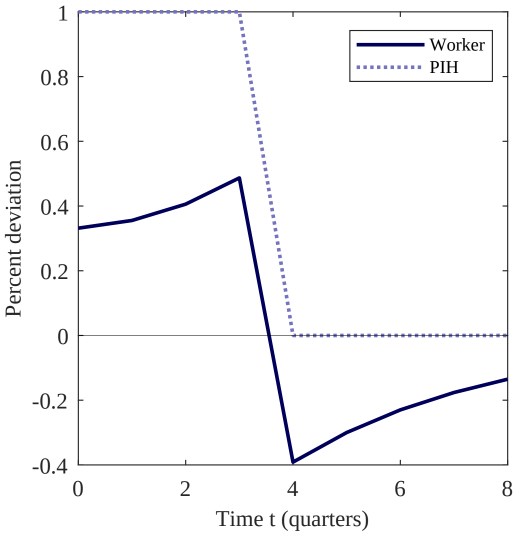

(d) Effect on consumption of news about an interest rate cut at different shock horizons

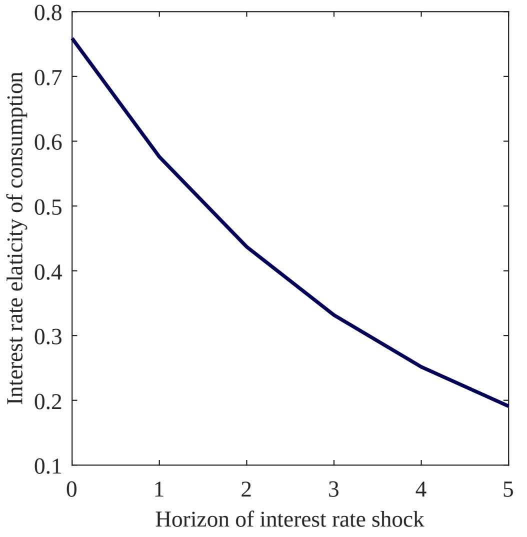

Figure A.2: Interest rate effects on consumption in the model with portfolio adjustment costs

Notes: The two left panels describes the dynamic effect on consumption of a one percent decline in the real interest rate, with $s = 0$ and $s = 3$, respectively. Adjustment costs are set to $\psi^W = 0.07$, as in the main text. The top right model plots $|\frac{d\hat{c}_t^W}{d\hat{r}_t}|$ for different values of ψ^W. The bottom right panel plots $\frac{d\hat{c}_t^W}{d\hat{r}_{t+s}}$ for different horizons s.

Appendix B TANK models

In this section we summarize the equilibrium as well as steady-state conditions for the simple TANK models discussed in Section 2. Notation is as in the main text. We also show the sensitivity of each model to the two key parameters, bond adjustment costs ψ^W and population share λ.

B.1 Simple TANK models

B.1.1 Equilibrium conditions

B.1.1.1 TANK-UH Table B.1 summarizes the non-linear equilibrium conditions of a simple two-agent New Keynesian model with unconstrained and hand-to-mouth households (TANK-UH).

Description	Equation
Euler equation U	$E_t c_{t+1}^U = \beta r_t c_t^U$
Budget constraint U	$c_t^U + b_t^U = n_t\, w_t - t_t + \frac{d_t}{1-\lambda} + r_{t-1}\, b_{t-1}^U$
Budget constraint H	$c_t^H = n_t\, w_t - t_t$
Aggregate consumption	$c_t = \lambda c_t^H + (1-\lambda) c_t^U$
Labor supply	$n_t^{\varphi} \nu = \frac{w_t}{c_t}$
Dividends	$d_t = y_t - n_t w_t$
Production	$y_t = n_t$
Marginal costs	$mc_t = w_t$
Phillips curve	$\left(1+\tau^S\right)(1-\eta) + \eta\, mc_t - \Pi_t\, \xi\, (\Pi_t - 1) +$
	$+\beta E_t\, \xi\, \frac{c_{t+1}^U}{c_t^U}\, \Pi_{t+1}\, (\Pi_{t+1}-1)\, \frac{y_{t+1}}{y_t} = 0$
Fisher equation	$r_t = E_t R_t / \Pi_{t+1}$
Government budget constraint	$b_t = g_t + r_{t-1}\, b_{t-1} - t_t$
Government spending	$g_t = \rho^g g_{t-1} + \epsilon_t^g$
Fiscal rule	$\frac{t_t - t}{y} = \phi^{\tau t} \frac{t_{t-1}-t}{y} + \phi^{\tau B} \frac{b_t - b}{y} + \phi^{\tau G} \frac{g_t - g}{y}$
Taylor rule	$\frac{R_t}{R} = \left(\frac{\Pi_t}{\Pi}\right)^{\phi^{\pi}}$
Bond holdings	$b_t = (1-\lambda) b_t^C$

Table B.1: Non-linear equilibrium conditions for the TANK-UH model

Notes: This table summarizes the non-linear equilibrium conditions of a simple two-agent New Keynesian model with unconstrained (U) and hand-to-mouth (H) households.

In Section 2.1.1 we consider a log-linearized version of the model. The equilibrium conditions are approximated around the zero-inflation steady state ($\Pi = 1$), in which hours worked are normalized to unity ($n = y = 1$). Then from the Euler condition of unconstrained households we have that $R = \frac{1}{\beta}$. An optimal production subsidy $\tau^S = (\eta - 1)^{-1}$ ensures zero profits in equilibrium, so that $w = mc = (1 + \tau^S)\frac{\eta-1}{\eta} = 1$ and $d = 0$. For simplicity, here we assume zero government spending and debt in steady state ($b = b^U = g = t = 0$). Given equal hours, we have an equal-consumption result; $c = c^U = c^H = 1$. Finally $\nu = \frac{w}{cn^{\varphi}} = 1$.

B.1.1.2 TANK-UW The equilibrium conditions of what in our nomenclature is called the TANK-UW model are identical to those summarized in Table B.1 with the exception of an additional variable describing the worker household's bond holdings, an associated Euler equation, and a modified aggregation of bond holdings:

$$E_t c^W_{t+1}\left(1 + \psi^W/c^W\left(b^W_t - b^W\right)\right) = \beta\, r_t\, c^W_t,$$

$$c^W_t + b^W_t = n_t\, w_t - t_t + r_{t-1}\, b^W_{t-1},$$

$$b_t = \lambda b^W_t + (1 - \lambda) b^U_t.$$

The steady-state conditions are as in the UH model, with the additional stipulation that $b^W = 0$.

B.1.1.3 TANK-CW The non-linear equilibrium conditions of a simple two-agent New Keynesian model with capitalists and workers households (TANK-CW) are equivalent to the conditions summarizing the UW model with three exceptions: the budget constraint of capitalists includes no labor income; aggregate labor supply is replaced by a condition referring just to workers; and budget constraints include a lump-sum tax/transfer tr^W to preserve zero consumption inequality in steady state. Table B.2 summarizes.

As regards the steady state, workers' labor is now pinned down by $n^w = \frac{n}{\lambda} = \frac{1}{\lambda}$. To ensure equal consumption in steady state, we introduce a lump-sum transfer from workers to capitalists

Description	Equation
Euler equation C	$E_t c^C_{t+1} = \beta r_t c^C_t$
Budget constraint C	$c^C_t + b^C_t = \frac{d_t}{1-\lambda} - t_t + r_{t-1}\, b^C_{t-1} + \frac{tr^W}{1-\lambda}$
Euler equation W	$E_t c^W_{t+1}\, \left(1 + \psi^W / c^W\, \left(b^W_t - b^W\right)\right) = \beta\, r_t\, c^W_t$
Budget constraint W	$c^W_t + b^W_t = w_t\, n^W_t - t_t + r_{t-1}\, b^W_{t-1} - \frac{tr^W}{\lambda}$
Labor supply W	$n^{W\varphi}_t \nu = \frac{w_t}{c^W_t}$
Aggregate consumption	$c_t = \lambda c^W_t + (1-\lambda) c^C_t$
Aggregate hours	$n_t = \lambda n^W_t$
Dividends	$d_t = y_t - n_t w_t$
Production	$y_t = n_t$
Marginal costs	$mc_t = w_t$
Phillips curve	$\left(1+\tau^S\right)\, (1-\eta) + \eta\, mc_t - \Pi_t\, \xi\, (\Pi_t - 1) +$
	$+\beta E_t\, \xi\, \frac{c^C_{t+1}}{c^C_t}\, \Pi_{t+1}\, (\Pi_{t+1} - 1)\, \frac{y_{t+1}}{y_t} = 0$
Fisher equation	$r_t = E_t R_t / \Pi_{t+1}$
Government budget constraint	$b_t = g_t + r_{t-1}\, b_{t-1} - t_t$
Government spending	$g_t = \rho^g g_{t-1} + \epsilon^g_t$
Fiscal rule	$\frac{t_t - t}{y} = \phi^{\tau t} \frac{t_{t-1} - t}{y} + \phi^{\tau B} \frac{b_t - b}{y} + \phi^{\tau G} \frac{g_t - g}{y}$
Taylor rule	$\frac{R_t}{R} = \left(\frac{\Pi_t}{\Pi}\right)^{\phi^\pi}$
Bond holdings	$b_t = \lambda b^W_t + (1-\lambda) b^C_t$

Table B.2: Non-linear equilibrium conditions for the TANK-CW model

Notes: This table summarizes the non-linear equilibrium conditions of a simple two-agent New Keynesian model with capitalists (C) and workers (W).

$tr^W = ((wn^W) - c^W - t)\lambda = 1 - \lambda$. The labor dis-utility weight is now $\nu = \frac{w}{c^w n^{W\varphi}} = \lambda^\varphi$. Then the remaining conditions are unchanged.

B.2 Sensitivity results

B.2.1 Calibration with equal population shares

We start this section by presenting an alternative calibration of λ and ψ^W. While in 3.1 they were set to target both quarterly and annual impact iMPC evidence, here we compare models where each agents represent half of the population in the economy, that is, $\lambda = 0.5$, and then set PACs to

match the quarterly impact MPC ($\psi^W = 0.2265$). This calibration generates more heterogeneous average iMPCs across models and helps highlighting the impact of our modeling choices on the aggregate labor supply and fiscal multiplier. Figure B.1 shows that, keeping population proportions constant, the introduction of workers reduces by half the output multiplier for a deficit-financed government spending shock. This result occurs due to the lower consumption propensity of workers compared to hand-to-mouth households. Introducing, on top of that, capitalists instead of the usual unconstrained households drives the impulse responses of hours and, hence, output to almost zero in the resulting TANK-CW model. This is because the labor supply curve of households suffering a loss in profit income (due to markups moving countercyclically) does not shift outwards. Hence this calibration implies virtually no aggregate expansionary effects of fiscal stimulus, while preserving the same redistributive movements in real wages and profits as observed in the TANK-UW model with unconstrained households and workers.

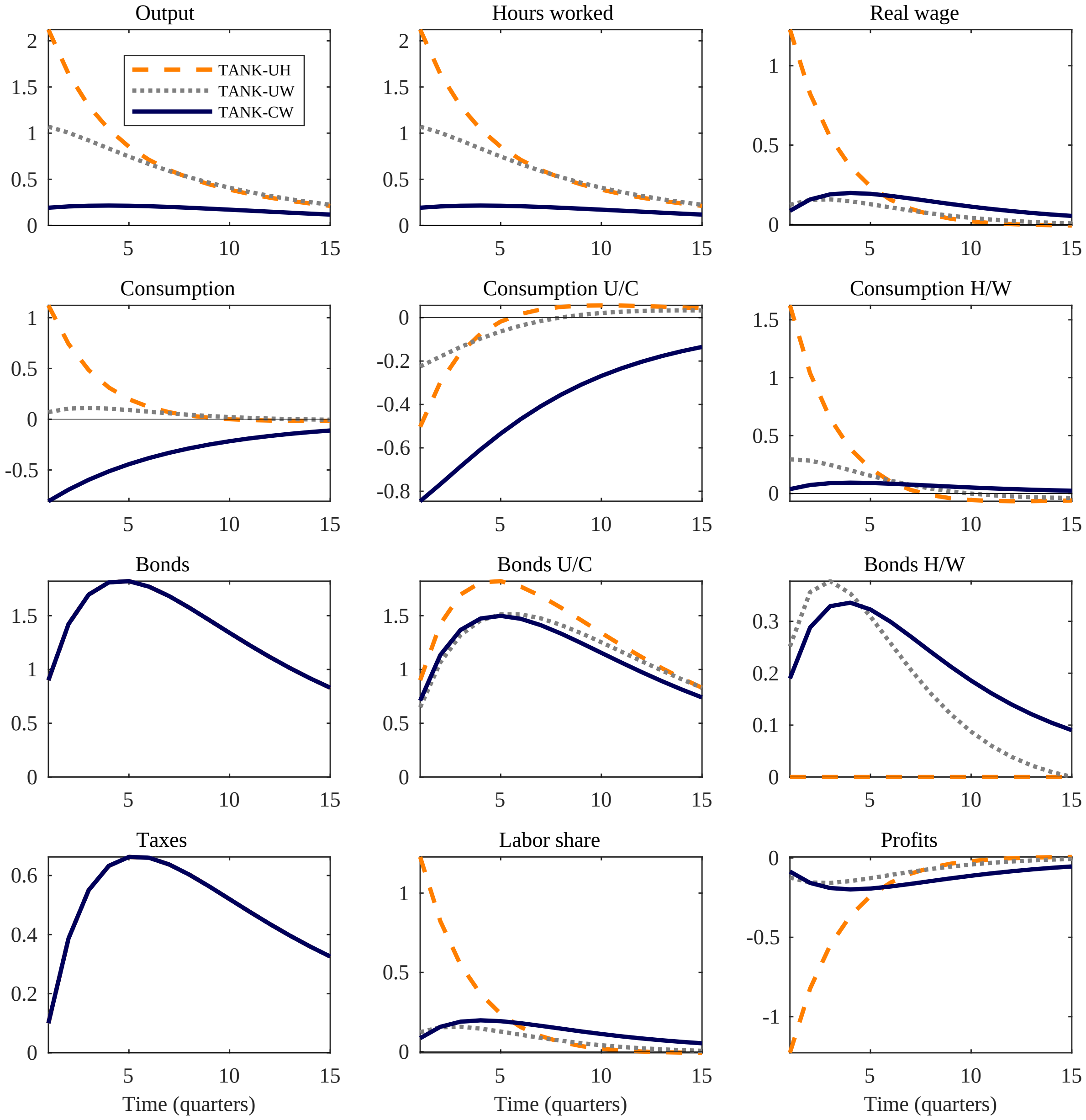

Figure B.1: Government spending shocks in the simple TANK models keeping $\lambda = 0.5$

Notes: The figure shows the impulse responses of selected variables to a government spending shock according to different, simple TANK models. All series are in proportional deviations from their steady state (in %) except for the fiscal variables (government spending, bonds and taxes) and profits, which are measured in percentage of steady-state output. Consumption components are weighted by population shares. Explanations for the acronyms are as follows: UH – unconstrained and hand-to-mouth households; UW – unconstrained and worker households; CW – capitalist and worker households.

B.2.2 Variations in λ

Next, consider variations in the population share parameter λ, holding all other parameter values constant. Figures B.2-B.4 reveal that a higher value of λ generates a more positive consumption and output response in all three model variants. This result follows directly from household heterogeneity and the fact that both hand-to-mouth and workers' (impact) MPCs (and for workers also MPCs in subsequent periods) are higher than those of permanent-income consumers. Hence, raising the value of λ raises the higher average marginal propensity to consume.[B.1]

[B.1] Notice that we cannot simulate the TANK-UH model for a high value of λ such as $\lambda = 0.9$ due to indeterminacy issues, as discussed in Section C.

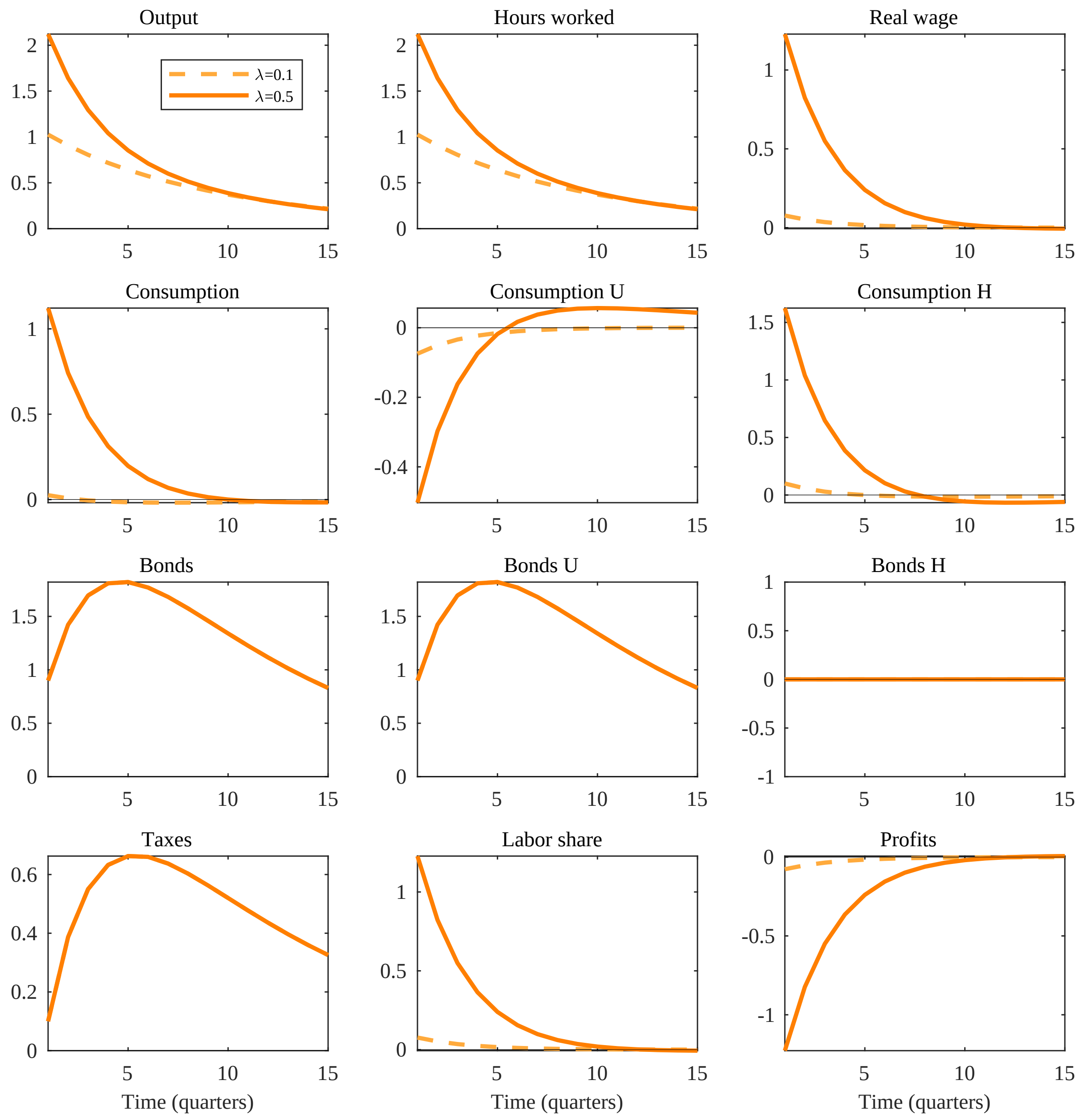

Figure B.2: Sensitivity to λ in TANK-UH

Notes: This figure shows the impulse responses of selected variables to a government spending shock for different values of λ in TANK-UH. All series are in proportional deviations from their steady state (in %) except for the fiscal variables (government spending, bonds and taxes) and profits, which are measured in percentage of steady-state output. Consumption components are weighted by population shares.

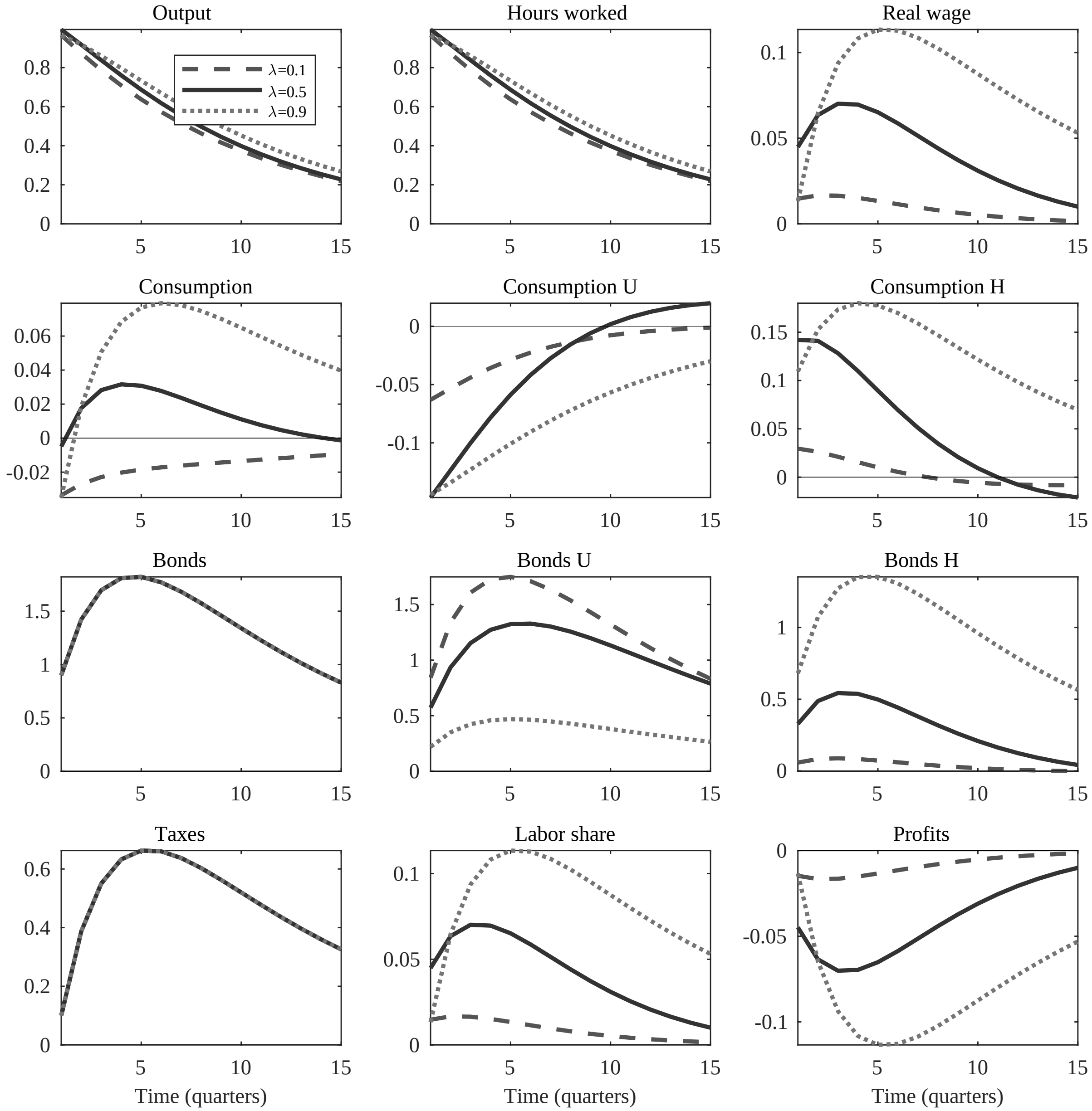

Figure B.3: Sensitivity to λ in TANK-UW

Notes: This figure shows the impulse responses of selected variables to a government spending shock for different values of λ in TANK-UW. All series are in proportional deviations from their steady state (in %) except for the fiscal variables (government spending, bonds and taxes) and profits, which are measured in percentage of steady-state output. Consumption components are weighted by population shares.

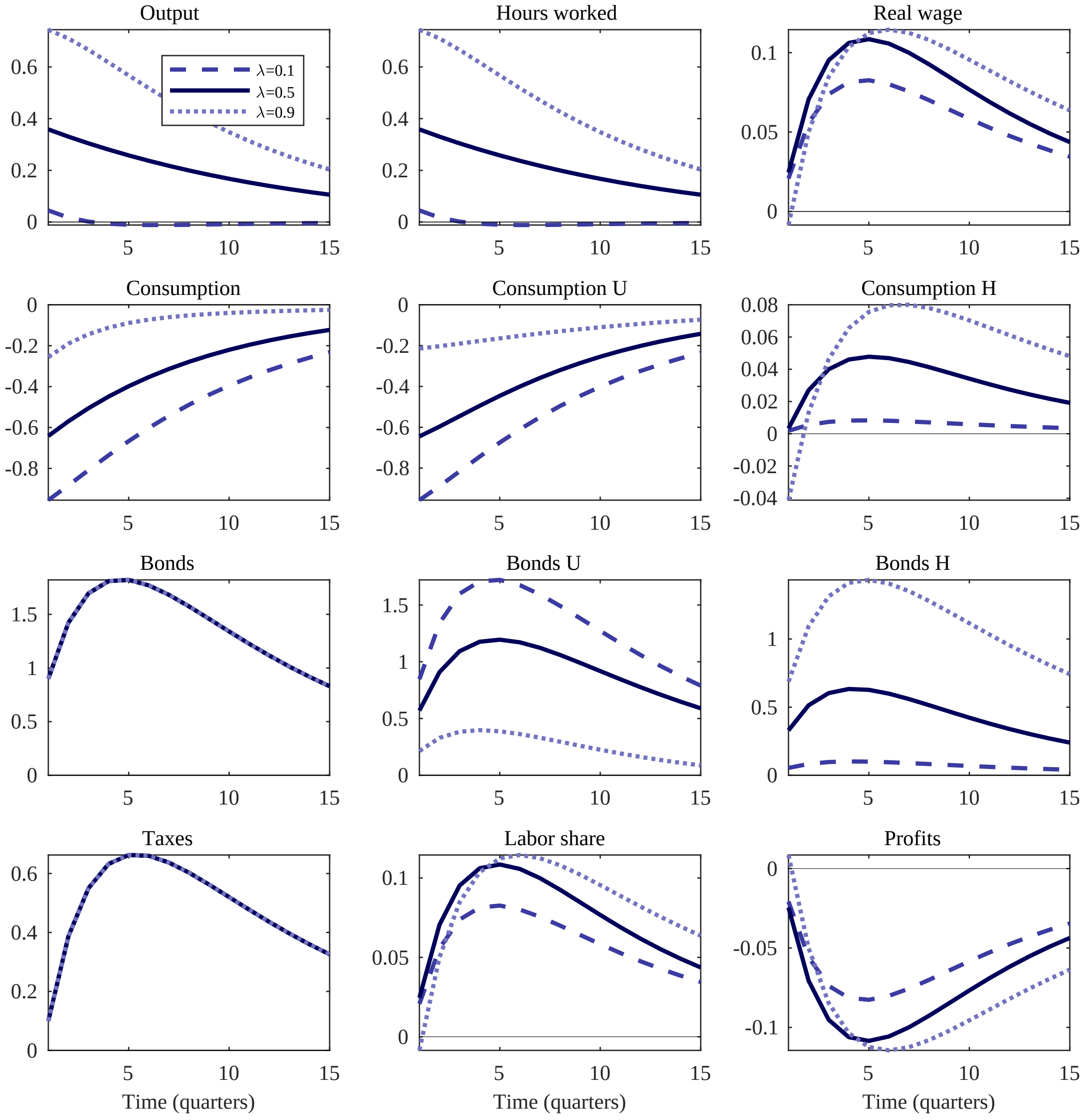

Figure B.4: Sensitivity to λ in TANK-CW

Notes: This figure shows the impulse responses of selected variables to a government spending shock for different values of λ in TANK-CW. All series are in proportional deviations from their steady state (in %) except for the fiscal variables (government spending, bonds and taxes) and profits, which are measured in percentage of steady-state output. Consumption components are weighted by population shares.

B.2.3 Variations in ψ^W

Turning to variations in the strength of portfolio adjustment costs, as indexed by ψ^W, we now show what happens in the TANK-UW and TANK-CW models when we raise ψ^W above the baseline level of $\psi^W = 0.0742$. This exercise serves to illustrate that the two differences between benchmark TANK model and the proposed alternative TANK-CW model interact. For recall that only in the UW model households with unconstrained access to financial markets (elastically) supply labor. In particular, Figure B.5 demonstrates that whereas in the UW model increasing the value of ψ^W reinforces both the aggregate and redistributive effect of fiscal policy, this result does not carry over to the CW model (Figure B.6). Instead, increasing the strength of PACs reinforces the *redistributive* effect of fiscal policy but reduces its impact on *aggregate* output. Intuitively, the reason is a higher value of ψ^W pushes up workers' consumption on impact, reducing their desire to work, ceteris paribus. Other things are not equal, however, because the expansion in demand also pushes up (down) wages (profits). Unlike in the UW model, however, this fails to trigger an increase in labor supply through a profit income effect. As a result, there is no compensating rise in total hours worked and, hence capitalists' income and aggregate output.

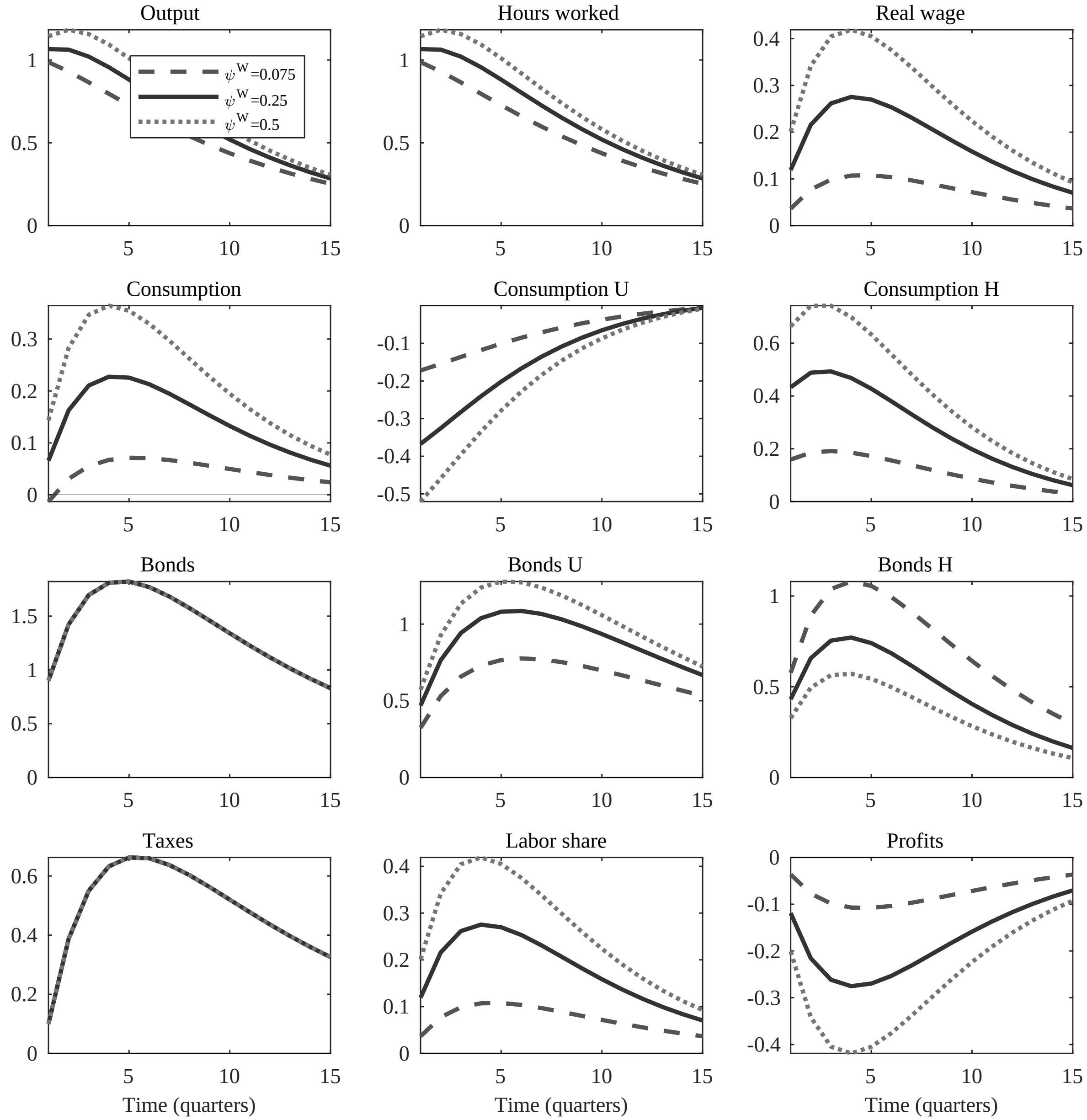

Figure B.5: Sensitivity to ψ^W in TANK-UW

Notes: This figure shows the impulse responses of selected variables to a government spending shock for different values of ψ^W in TANK-UW. All series are in proportional deviations from their steady state (in %) except for the fiscal variables (government spending, bonds and taxes) and profits, which are measured in percentage of steady-state output. Consumption components are weighted by population shares.

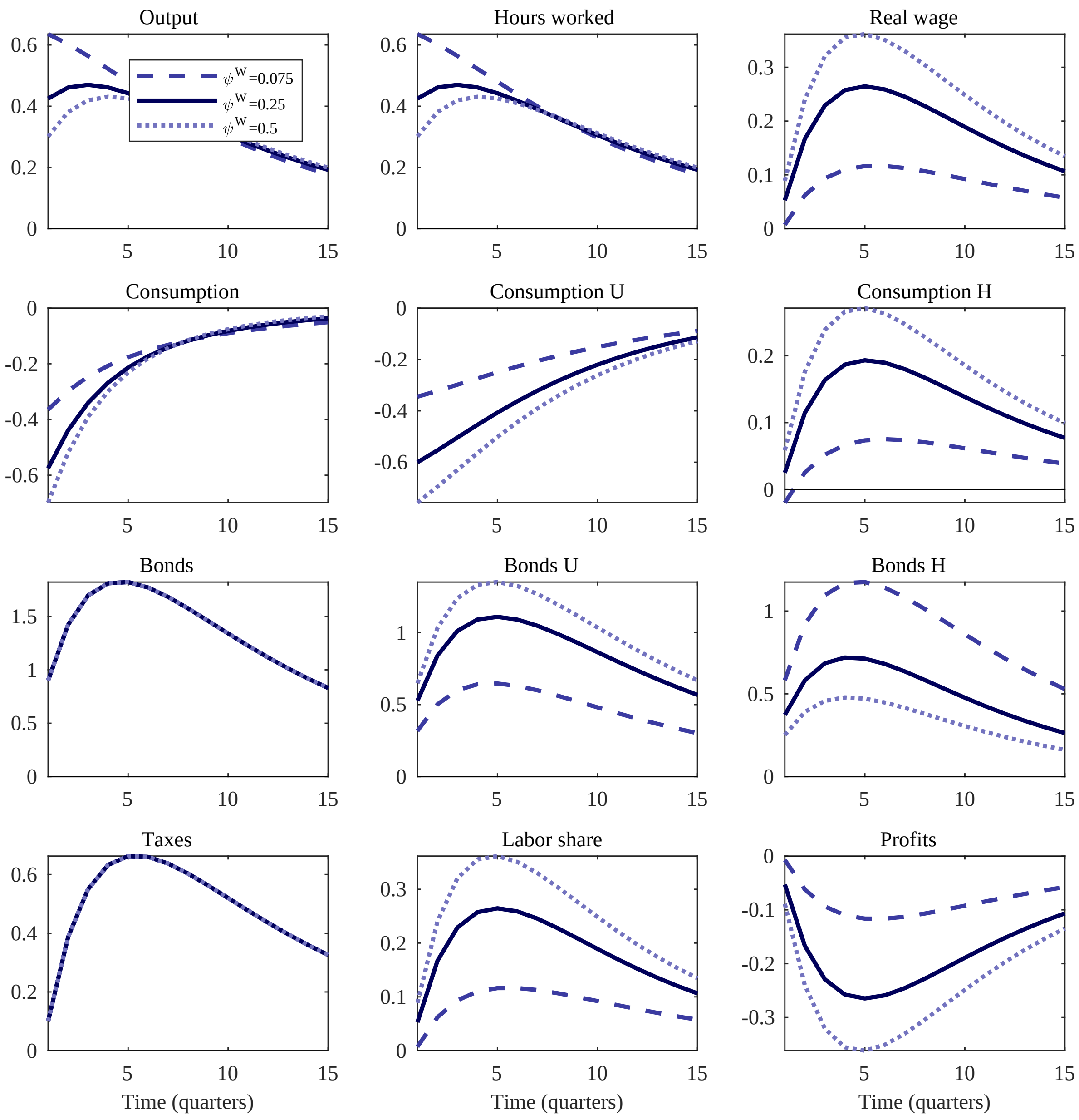

Figure B.6: Sensitivity to ψ^W in TANK-CW

Notes: This figure shows the impulse responses of selected variables to a government spending shock for different values of ψ^W in TANK-CW. All series are in proportional deviations from their steady state (in %) except for the fiscal variables (government spending, bonds and taxes), which are measured in percentage of steady-state output. Consumption components are weighted by population shares.

B.2.4 Variations in fiscal rule parameters

In Footnote 30 we remarked upon the fact that in Figure 5 the path of workers' consumption is lightly hump-shaped, a pattern that is inherited also by aggregate consumption. Since private consumption alongside public spending pins down labor demand in this model, the same shape is observed also for the labor share. This result is, however, contingent upon the particular fiscal rule we consider rather than being a direct consequence of workers' iMPCs; after all, the latter peak on impact. Figure B.7 illustrates this point by considering an altered parameterization of the fiscal rule, whereby the coefficient on debt is one and that to government spending is zero. Consequently, bonds peak on impact. It can be seen that the response of consumption and the labor share is no longer hump-shaped. Thus, the presence of portfolio adjustment costs generates a hump-shaped path for workers' consumption provided the path for the supply of liquidity in form of government bonds is hump-shaped as well. Intuitively, the greater the excess of bonds issued by the government to finance its additional spending relative to workers' target level, the greater is workers' desire to consume so as to avoid a penalty in the form of adjustment costs.

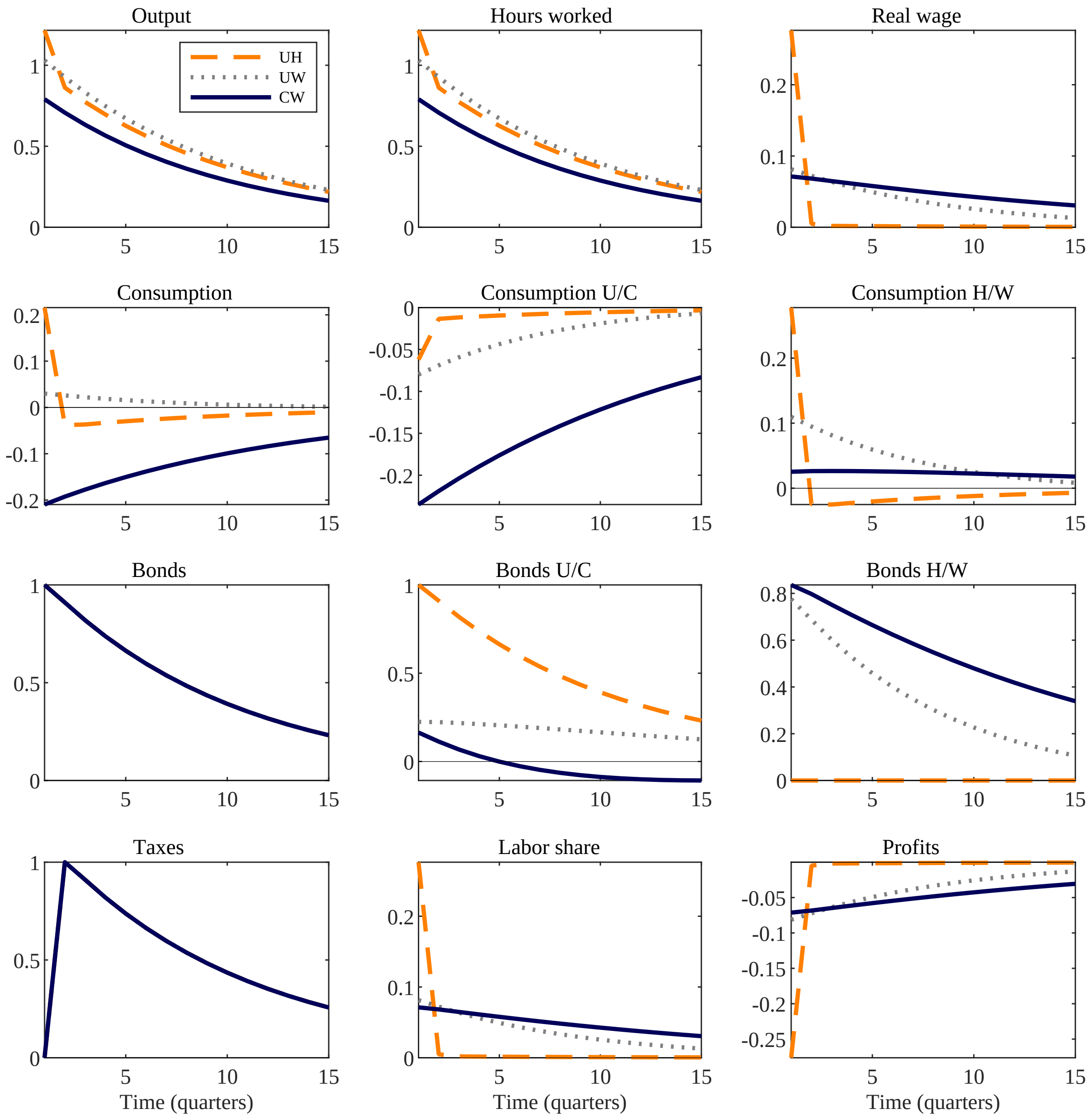

Figure B.7: Government spending shocks in the simple TANK models – amended fiscal rule

Notes: The figure shows the impulse responses of selected variables to a government spending shock equal to one percent of steady-state output in different, simple TANK models when in the tax rule the coefficient on debt is one and that to government spending is zero. All series are in percent deviations from their steady state except for the fiscal variables (government spending, bonds and taxes) and profits, which are measured in percentage of steady-state output. Consumption components are weighted by population shares. Explanations for the acronyms: UH – unconstrained and hand-to-mouth households; UW – unconstrained and worker households; CW – capitalist and worker households.

B.3 Medium-scale models

We concluded our analysis in the main text, Section 3.3, by considering medium-scale variants of the different TANK models that allow for physical investment, nominal wage stickiness and other frictions commonly incorporated into medium-scale DSGE. Here we provide more details on these models. Importantly, capitalists receive income not only from holding firm equity – as in our simple variants, following Bilbiie (2008) – but they also invest in physical capital – as in Galí *et al.* (2007). Intermediate firms rent capital and use it alongside labor according to a Cobb-Douglas production function. Rigidity in nominal wages is modeled as for prices using Rotemberg adjustment costs. As such, hours worked are largely demand-determined, worker households are not always on their labor supply curve, and the average marginal propensity to earn is low (on the importance of which for consistency with empirical data, see Auclert *et al.* (2020b)). In addition, we allow for fixed costs in production, investment adjustment costs, variable capital utilization, a more general Taylor rule featuring interest rate smoothing and, finally, positive government spending as well as debt in steady state.

Table B.3 summarizes the non-linear equilbrium conditions of the medium-scale TANK-CW model.[B.2] Relative to the simple model described in Appendix Section B, new endogenous variables are as follows: y^m gross output (includes fix costs of production); capital, k; investment, i; the real rental rate r^K; investment adjustment costs s, which imply that Tobin's q, denoted q, is no longer necessarily equal to unity; utilization of capital, u, and the associated cost Ψ; workers' marginal rate of substitution, mrs. Given nominal wage stickiness, in the form of Rotemberg adjustment costs, we also have gross wage inflation, Π^w. Notice, furthermore, that now variables in the fiscal rule are expressed in deviations from their own (positive) steady-state. It would be straightforward to add various other common frictions such as habits or firm-specific capital.

As far as additional parameter values are concerned, notice that α denotes the capital share, η^W

[B.2] The UW variant can be obtained by replacing capitalist-type with unconstrained-type households and adding back the aggregate labor supply equation. The benchmark UH model is instead obtained by replacing worker-type with hand-to-mouth households who do not participate in financial markets at all.

the elasticity of substitution between differentiated labor, ξ^w Rotemberg wage adjustment costs, $\upsilon = \frac{\gamma^1}{\gamma^2}$ is the utilization elasticity, ϕ^r is interest rate inertia while ϕ^y is the interest rate response to output. Steady-state relationships involve calibrating the steady state government spending and debt to output ratios (respectively $\frac{g}{y}$ and $\frac{b}{y}$ and the parameter ν scaling the dis-utility of working: $\nu = \frac{w\frac{\eta^w-1}{\eta^w}}{n^{W\varphi}}$. Additionally, we impose fixed costs in production to ensure zero monopoly profits in steady-state, $F = n\left(\left(\frac{k}{n}\right)^{\alpha} - \left(w + r^K\frac{k}{n}\right)\right)$. As before to ensure equal consumption in steady-state, we introduce a lump-sum transfer from workers to capitalists $tr^W = ((wn^W) - c^W - t)\lambda = 1 - \lambda$. Table B.4 summarize the parameterization, which is conventional.

In Section 3.3 we highlighted two main results from the consideration. First, cumulative output multipliers are more similar across the different medium-scale TANK variants. And second, once important features such as elastic investment in physical capital are allowed for, the TANK-CW model generates impulse responses for consumption and investment that are consistent with evidence from macro time series, as considered, for instance, in Section D. In particular, aggregate consumption exhibits a positive but relatively short-lived response to an expansionary, deficit-financed increase in public spending. In addition to elastic investment, which makes capitalists' consumption response less negative, allowing for (empirically plausible) real interest rate smoothing also plays an important role. In its absence, consumption by workers, in particular, is more negative. This result, too, can be directly tied to the partial equilibrium model analyzed in Section 2.2. There we noted that, unlike for hand-to-mouth households, workers' consumption is somewhat responsive to real interest rate movements, especially at a near horizon. Consequently, if the central bank raises real interest rates more slowly following an expansion in public spending that pushes up inflation, workers will consume more.

Description	Equation
Euler equation C	$E_t c^C_{t+1} = \beta r_t c^C_t$
Euler equation W	$E_t c^W_{t+1} \left(1 + \psi^W / c^W \left(b^W_t - b^W\right)\right) = \beta\, r_t\, c^W_t$
Budget constraint W	$c^W_t + b^W_t = n^W_t\, w_t - t_t + r_{t-1}\, b^W_{t-1} - \frac{tr^W}{\lambda}$
Labor supply W	$mrs_t = \nu^W\, n^{W\varphi}_t c^W_t$
Aggregate consumption	$c_t = \lambda c^W_t + (1-\lambda) c^C_t$
Aggregate hours	$n_t = \lambda n^W_t$
Aggregate capital	$k_t = (1-\lambda) k^C_t$
Aggregate investment	$i_t = (1-\lambda) i^C_t$
Resource constraint	$y_t = i_t + c_t + g_t + k_{t-1}\, \Psi_t$
Dividends	$d_t = y_t - n_t w_t - r^k_t u_t k_{t-1}$
Production	$y^m_t = n^{1-\alpha}_t\, (u_t\, k_{t-1})^{\alpha}$
Net output	$y_t = y^m_t - F$
Real wages	$w_t = mc_t\, (1-\alpha)\, (n_t/(u_t\, k_{t-1}))^{(-\alpha)}$
Rental rate of capital	$r^k_t = mc_t \alpha\, ((u_t\, k_{t-1})/n_t)^{\alpha-1}$
Capital utilization 1	$\Psi_t = \gamma^1\, (u_t - 1) + \frac{\gamma^2}{2}\, (u_t - 1)^2$
Capital utilization 2	$r^k_t = \gamma^1 + (u_t - 1)\, \gamma^2$
Capital accumulation	$k^C_t = (1 - s_t)\, i^C_t + (1-\delta)\, k^C_{t-1}$
Investment adjustment costs	$s_t = \iota \left(i^C_t / i^C_{t-1} - 1\right)^2$
Tobin's q	$q_t = \frac{1}{r_t} E_t \left(u_{t+1}\, r^k_{t+1} - \Psi_{t+1} + (1-\delta)\, q_{t+1}\right)$
Investment	$q_t \left(1 - s_t - i^C_t / i^C_{t-1}\, s'_t\right) + E_t q_{t+1}\, c^C_t \beta / c^C_{t+1}\, s'_{t+1}\, (i^C_{t+1}/i^C_t)^2 = 1$
Phillips curve	$1 - \eta + \eta\, mc_t - \Pi_t\, \xi\, (\Pi_t - 1) + \beta E_t\, \xi\, \frac{c^C_{t+1}}{c^C_t}\, \Pi_{t+1}\, (\Pi_{t+1} - 1)\, \frac{y_{t+1}}{y_t} = 0$
Wage Phillips curve	$1 - \eta^w + \frac{mrs_t\, \eta^w}{w_t} - \Pi^w_t\, \xi^w\, \left(\Pi^w_t - 1\right) +$
	$+\beta\, E_t \xi^w\, \frac{c^W{}_t}{c^W{}_{t+1}}\, \Pi^w_{t+1} \left(\Pi^w_{t+1} - 1\right)\, \frac{w_{t+1}\, n_{t+1}}{w_t\, n_t} = 0$
Wage inflation	$\Pi^w_t = w_t / w_{t-1}$
Fisher equation	$r_t = E_t R_t / \Pi_{t+1}$
Government budget constraint	$b_t = g_t + r_{t-1}\, b_{t-1} - t_t$
Government spending	$log\left(\frac{g_t}{g}\right) = \rho^g log\left(\frac{g_{t-1}}{g}\right) + \epsilon^g_t$
Fiscal rule	$\frac{t_t}{t} = \phi^{\tau t}\, \frac{t_{t-1}}{t} + \phi^{\tau B}\, \frac{b_t}{b} + \phi^{\tau G}\, \frac{g_t}{g}$
Taylor rule	$\frac{R_t}{R} = \phi^r\, \frac{R_{t-1}}{R} (1-\phi^r) \left(\left(\frac{\Pi_t}{\Pi}\right)^{\phi^\pi} + \left(\frac{Y_t}{Y}\right)^{\phi^y}\right)$
Bond holdings	$b_t = \lambda b^W_t + (1-\lambda) b^C_t$

Table B.3: Non-linear equilibrium conditions for the TANK-CW model

Notes: This table summarizes the non-linear equilibrium conditions of the medium scale two-agent New Keynesian model with capitalists and workers. The capitalist household's budget constraint may be omitted given Walras' Law.

Parameter	Interpretation	Value (H | W)	Source
β	Discount factor	0.99	Annual real interest rate of 4%
ρ^G	AR1 Government spending shock	0.9	Benchmark
ψ^W	Portfolio adjustment cost	∞ | 0.07	Definition | iMPC evidence
λ	% of H/W	0.19 | 0.8	iMPC evidence
b^W	Workers' steady-state bond holdings	0	Comparability of models
ξ	Rotemberg price stickiness	42.68	Average price duration 3.5q
ϕ^π	Interest rate response to inflation	1.5	Galí *et al.* (2007)
$\phi^{\tau,t}$	Tax smoothing	0	Galí *et al.* (2007)
$\phi^{\tau,g}$	Tax response to government spending	0.1	Galí *et al.* (2007)
$\phi^{\tau,b}$	Tax response to debt	0.33	Galí *et al.* (2007)
Π	Steady-state inflation rate	1	Benchmark
φ	Inverse Frisch elasticity	1	Benchmark
g/y	Steady-state government spending output ratio	0.2	Average across sample
b/y	Steady-state debt output ratio	4×0.57	Average across sample
$1-\alpha$	Steady-state labor share	0.67	Average
n	Steady-state labor supply	1/3	Benchmark
υ	Capital utilization	0.495	Altig *et al.* (2011)
η	Intermediate goods elasticity of substitution	6	SS price mark-up of 20%
η^w	Differentiated labor elasticity of substitution	6	SS wage mark-up of 20%
ξ^w	Rotemberg wage adjustment costs	42.68	Average wage duration 3.5q
ι	Investment adjustment costs	2	Benchmark
ϕ^r	Interest rate smoothing	0.7	Benchmark
ϕ^y	Interest rate response to output	0	Benchmark

Table B.4: Parameter values for the medium-scale models

Notes: This table lists the parameter values of the medium-scale TANK models. One period in the model corresponds to one quarter. Explanations for the acronyms: H – hand-to-mouth; W – worker; UH – unconstrained and hand-to-mouth households.

Appendix C Determinacy in TANK models

In the main text, we focused on consumption dynamics and the determinants of labor supply in different TANK models. Here we draw attention to a third dimension along which the traditional characterization of household heterogeneity in a two-agent setting and our proposed amendment differ. That is, we show that a model with intermediate portfolio adjustment costs has more plausible determinacy properties than the benchmark TANK model, in that it does not require an implausible value of the Frisch elasticity for determinacy, and remains determinate under an active Taylor rule for all plausible values of the population share of constrained households.

Our starting point is that for the benchmark TANK model to have a unique saddle-path stable solution under the standard Taylor principle, it is necessary to either assume a relatively low share of hand-to-mouth households; or to impose a Frisch elasticity of labor supply (marginal disutility of labor φ) that is very high (low) relative to the values typically found in the empirical literature.[C.1] Figure C.1 concisely summarizes this point. It plots in the parameter space $(\lambda, \varphi, \phi^{\pi})$ the regions that are associated with the presence of uniqueness and multiplicity of the rational expectations equilibrium in a neighborhood of the steady-state, respectively. For a sufficiently strong degree of non-participation in asset markets and/or a labor supply curve that is relatively inelastic, the solution is indeterminate if $\phi^{\pi} > 1$.[C.2]

To add intuition, Bilbiie (2008) shows that it is the interplay between labor markets and asset markets described in Section 2.3 above that underpins these determinacy properties of the TANK-UH model. This point is most easily seen in a special case of the model that imposes budget balance and zero persistence government spending, and assumes that the central bank responds to expected

[C.1] For surveys of labor supply elasticity estimates, see, e.g., Chetty *et al.* (2013) and Attanasio *et al.* (2018).

[C.2] On this point, also see Gali *et al.* (2004), Galí *et al.* (2007) and Bilbiie (2008), but also note Maliar and Naubert (2019).

next-period inflation. Then the model can be reduced to a two-equation system:

$$\hat{c}_t = E_t \hat{c}_{t+1} - \zeta \left(E_t \hat{\Pi}_{t+1}(\phi^{\pi} - 1) + \epsilon_t^m \right) - \chi^{-1} \frac{\lambda \varphi}{\lambda - 1} \left(\epsilon_t^g - \epsilon_{t+1}^g \right) \tag{C.1}$$

$$\hat{\Pi}_t = \beta E_t \hat{\Pi}_{t+1} + \frac{(1-\theta)(1-\beta\theta)}{\theta} \left(\kappa \hat{c}_t + \frac{\varphi + 1}{\varphi^{-1} + 1} \epsilon_t^g \right), \tag{C.2}$$

where $\zeta = \frac{1-\lambda}{1-\lambda\chi}$ and $\kappa = \frac{\chi+\varphi^{-1}}{\varphi^{-1}+1}$, with $\chi = \varphi + 2$ denoting the elasticity of hand-to-mouth households' consumption to aggregate income.[C.3] The crucial parameters pinning down the stability properties of the system are those appearing in ζ, which is interpretable as the elasticity of aggregate demand with respect to the real interest rate. These parameters are the share of hand-to-mouth households λ and the inverse Frisch elasticity φ. When ζ is strictly positive, the standard Taylor principle applies. Aggregate demand becomes a *negative* function of the real interest rate, however, when $\lambda > \frac{1}{2+\varphi}$. Under this "inverted aggregate demand logic," the central bank needs to obey an inverted Taylor principle ($\phi_\pi < 1$) for stability to obtain. If asset market participation is sufficiently limited (λ is high) and/or labor supply inelastic enough (φ is high), a fall in the real interest rate can become contractionary due to the negative demand effect arising from the strong fall in profit income – $(1-\lambda)^{-1} > 1$ units per type-U household for any unit drop in total profits d_t – that occurs due to hand-to-mouth households' high marginal propensity to consume, which amplifies the rise and wages (fall in profits) relative to the case where all households smooth consumption by participating in financial markets. Under the standard Taylor principle, a non-fundamental increase in inflation expectations can then be self-fulling: it triggers a rise in the real interest rate which pushes up demand, output, and inflation, thus validating expectations.

Introducing even moderate portfolio adjustment costs gives rise to more plausible determinacy properties. Paralleling the above analysis, but now for the model with workers rather than hand-to-mouth households, Figure C.2 plots the stability properties of the rational expectations equilibrium (in a neighborhood of the steady-state) as a function of the population share parameter λ, the inverse

[C.3] Differences from the value of the same elasticity reported in Bilbiie (2019) are due to different assumptions on the determination of labor supply – other things equal, the value of χ is higher in the present setup – and the fact that we abstracted from fiscal redistribution of profits.

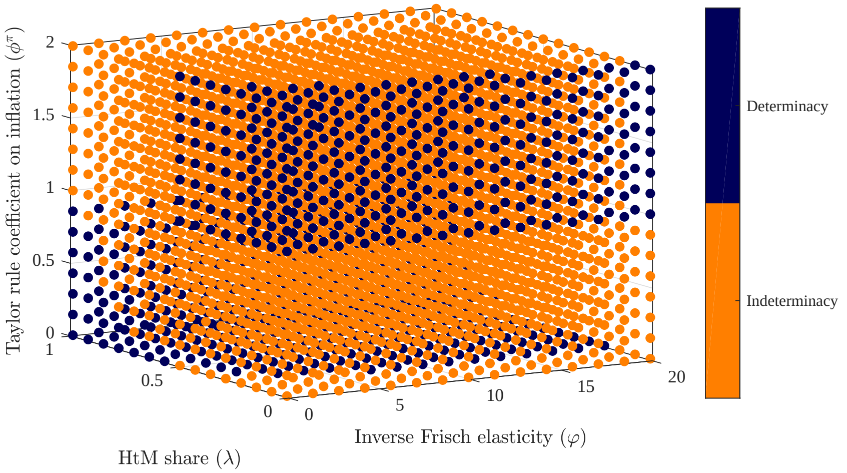

Figure C.1: Stability regions in the benchmark TANK-UH model

Notes: This figure shows regions in parameter space that are associated with the presence of uniqueness and multiplicity of the rational expectations equilibrium in a neighborhood of the steady-state, respectively. For details on the values of other parameters, which the plots are conditional on, see Table 2.

Frisch elasticity φ, as well as the strength of PACs as indexed by ψ^W (Panel C.2a) or the Taylor rule coefficient on inflation ϕ^{π} (Panel C.2b). For higher values of ψ^W the behavior of workers approximates that of hand-to-mouth households and, consequently, the indeterminacy problems for parameter combinations other than low λ and low φ reappear under a conventional value of $\phi_{\pi} = 1.5$. By contrast, for lower values the standard Taylor principle is restored for *any* combination of λ and φ. The reason is that the introduction of a *partially* constrained worker household type implies a less stringent interdependence of labor and financial markets. In particular, for values of the adjustment cost parameter ψ^W below infinity, workers' consumption propensity lies below that of hand-to-mouth households. The elasticity of real wages and, hence, profits, to output is consequently lower for smaller values of ψ^W (also see Figure B.5). Hence, the "inverted aggregate demand logic" region shrinks as ψ^W declines (Panel C.2a). In particular, when λ and ψ^W are calibrated to match micro consumption data as in our baseline calibration, the standard Taylor principle is restored (Panel C.2b).

Lastly, we observe that in the TANK framework introducing capitalists is feasible without compromising on the stability properties only in the presence of intermediate portfolio adjustment

(a) Stability in $(\varphi, \lambda, \psi^W)$ space (b) Stability in $(\varphi, \lambda, \phi^\pi)$ space

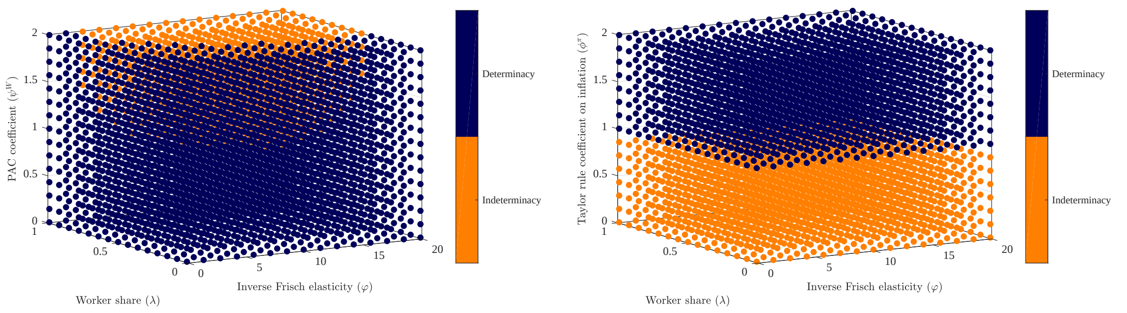

Figure C.2: Stability regions in the model with portfolio adjustment costs

Notes: This figure shows regions in parameter space that are associated with the presence of uniqueness and multiplicity of the rational expectations equilibrium in a neighborhood of the steady-state, respectively. For details on the values of other parameters, which the plots are conditional on, see Table 2. Notably, panel C.2a assumes $\phi^\pi > 1$.

costs. Suppose, for the sake of argument, that we are back in the setting with hand-to-mouth households (that is, PACs are infinitely large). Then after replacing aggregate labor supply with an equation for hand-to-mouth households only, a few substitutions show that $\hat{n}_t = \frac{\tilde{g}_t}{1+\varphi}$. Thus, hours worked are independent of profits. Unfortunately, however, solving the problem of profit income effects in this way exacerbates the determinacy problem that already afflicted the benchmark TANK-UH specification: irrespective of the value of λ a unique equilibrium is now unattainable given $\phi_\pi > 1$.[C.4] Panel C.3a indicates that there are no combinations of λ and φ for which the rational expectations equilibrium is locally unique given an active Taylor principle $\phi_\pi > 1$. under an active Taylor principle.

Panel C.3b instead shows that the introduction of an intermediate level of portfolio adjustment costs, with $\psi^W = 0.0742$, restores this possibility. With the exception of the corner case $\lambda \to 0$ that is irrelevant for all practical purposes, there is a unique saddle-path stable solution irrespective of the value of λ and φ.[C.5]

[C.4] More formally, if we write the system as $E_t z_{t+1} = \Gamma_0 z_t + \Gamma_1 \nu_t$, where $z_t(c_t, \Pi_t)'$ and $\nu_t = (\epsilon_t^m, \epsilon_t^g)'$, then for any $\phi^\pi > 1$ it is never the case that both eigenvalues of Γ_0 are outside the unit circle, as required for determinacy (since both consumption and inflation are forward-looking variables).

[C.5] It can be shown that the model with capitalists and hand-to-mouth households can be represented by the same system (C.1)-(C.2), except that the slope of the demand curve now is $\zeta^{CH} = \frac{1-\lambda\chi^{CH}}{1-\lambda}$ and the coefficient on consumption in (C.2) curve becomes $\kappa^{CH} = \frac{\chi^{CH}(\lambda\varphi+1)}{(1+\varphi)}$. While $\zeta^{CH} < 0$ once the profit income effect on labor supply is removed, we now have that $\kappa^{CH} < 0$. Consider then once more a non-fundamental increase in inflation expectations. Under an active Taylor principle the real interest rises, pushing down consumption. But in the CH model this triggers a rise in

(a) Without PACs (b) With PACs

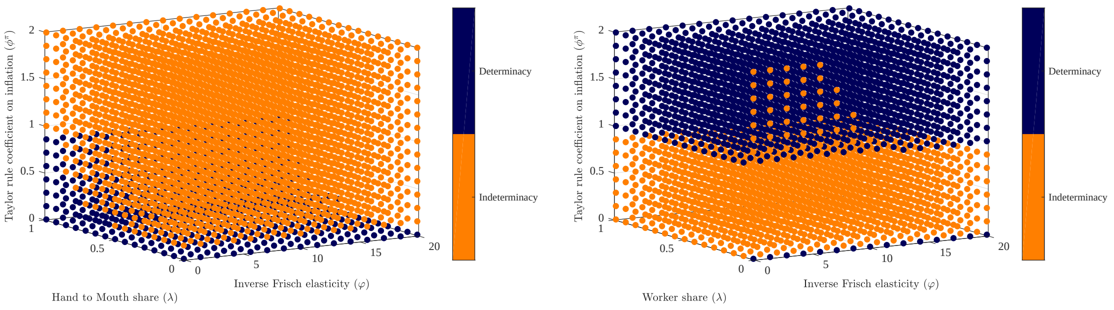

Figure C.3: Stability regions when adding capitalists: with and without portfolio adjustment costs

Notes: This figure shows regions in parameter space that are associated with the presence of uniqueness and multiplicity of the rational expectations equilibrium in a neighborhood of the steady-state, respectively. For details on the values of other parameters, which the plots are conditional on, see Table 2.

inflation that makes the initial sunspot shock self-fulfilling.

Appendix D Empirics

D.1 Baseline specification and results

Our baseline econometric tool to document the empirical effects of an unanticipated increase in government purchases is the structural vector autoregression (SVAR) approach devised by Forni and Gambetti (2016). It combines the recursive identification strategy of Blanchard and Perotti (2002) with a news variable constructed based on data from the Survey of Professional Forecasters (SPF). Jointly, these two components allow extracting "surprise" government spending shocks from the data. We find this approach appealing for two reasons. First, it allows purifying recursively identified shocks of any anticipated component. Second, unlike identification methods based on the use of defense spending, this methodology allows analyzing the response of the labor share to government spending shocks in general and not only the narrower subset of military spending shocks.[D.1]

The inclusion of the SPF variable is motivated by concerns over the implications of fiscal foresight. Intuitively, agents receive signals about fiscal changes prior to their implementation because of the existence of lags in the legislative and implementation process (for evidence of fiscal foresight see, among others, Ramey (2011); Leeper *et al.* (2013); Forni and Gambetti (2016)). Such fiscal foresight means that recursive identification, by itself, may not be sufficient to clearly distinguish between unanticipated and anticipated shocks, because some changes in fiscal expenditures are anticipated by agents even though they are unpredictable based on the variables in the econometrician's information set. Including the SPF news variable serves to enrich this information set and thus helps identify spending shocks "purified" of the anticipated component. Specifically, define the implied cumulated forecasts for government spending growth between $t = s$ and $t = h$, $s < h$ as $F_t(s,h) = \sum_{j=s}^{h} E_t^P g_{t+j}$,

[D.1]The empirical literature on fiscal policy offers a range of other approaches to identifying government spending shocks but is too extensive to summarize comprehensively. Relevant contributions include Caldara and Kamps (2008); Mountford and Uhlig (2009); Ramey (2011); Ben Zeev and Pappa (2015); Caldara and Kamps (2017); Ramey and Zubairy (2018). Several contributions have highlighted the challenge arising from fiscal foresight, including Yang (2005); Ramey (2011); Leeper *et al.* (2013); Forni and Gambetti (2016). Our identification approach is also related to that of Ricco (2015).

where E_t^P denotes the median expectation in the SPF in period t and g_{t+j} denotes the realized growth rate of government spending at $t + j$. In practice, we follow Forni and Gambetti (2016) and place $F_t(1, 4)$ as the second variable in the SVAR after government spending.

In terms of practical implementation, the benchmark specification is a ten-variable VAR estimated for the U.S. relying on quarterly data spanning from 1981:Q3 to 2007:Q4 and using standard Bayesian methods. The data comprise: log real government spending (consumption plus gross investment); the cumulated forecast of government spending growth over the next four quarters, $F_t(1, 4)$;[D.2] log real net taxes; log real GDP; log real consumption (durables and non-durables); log real investment; log labor share; log real corporate profits; the GDP deflator; and the 10-year real interest rate. The labor share deserves particular attention. Theoretically, it is defined as the share of total compensation of the labor force in the aggregate output of the economy. The empirical counterpart to this theoretical construct is ambiguous, however. As our baseline measure we use the labor share in the domestic corporate non-financial business sector, constructed in line with the methodology of Gomme and Rupert (2004).[D.3] All data sources are described in further detail below.

The starting date is dictated by the availability of SPF data for fiscal variables and coincides approximately with the beginning of the Great Moderation. The end date is prior to the start of the Great Recession to avoid potential structural breaks, but below we also report results obtained ending the sample in 2016, and using rolling windows. The lag length is chosen based on information criteria, which suggest the use of two lags for the baseline SVAR. The equations are estimated in

[D.2]Including the one-step-ahead forecast ($h = 1$) as the second variable in the SVAR, instead, and identifying the "purified" surprise spending shock as the first Cholesky shock would essentially be equivalent to the strategy followed by Auerbach and Gorodnichenko (2012) as well as Born *et al.* (2013). However, if the number of periods of anticipation exceeds one, then this variable will *not* include the news shock. By contrast, using $F_t(1, 4)$ as the news variable in the VAR increases the chances of capturing all relevant anticipation effects. We have also experimented with a news variable capturing expectations revisions ($N_t(1, 3)$ in the notation of Forni and Gambetti (2016) and the results are very similar to the $F_t(1, 4)$ approach.

[D.3]This approach is consistent with recent studies underscoring the importance of adjusting labor share measures for income from self-employment and housing in the context of lower-frequency movements in the labor share, an example being Gutierrez and Piton (2019). Also seem among others, Rognlie (2018). In the present context it is, furthermore, relevant that our benchmark measure excludes the public sector, alleviating any concerns that increased government spending on employment might mechanically increase the labor share of the economy as a whole.

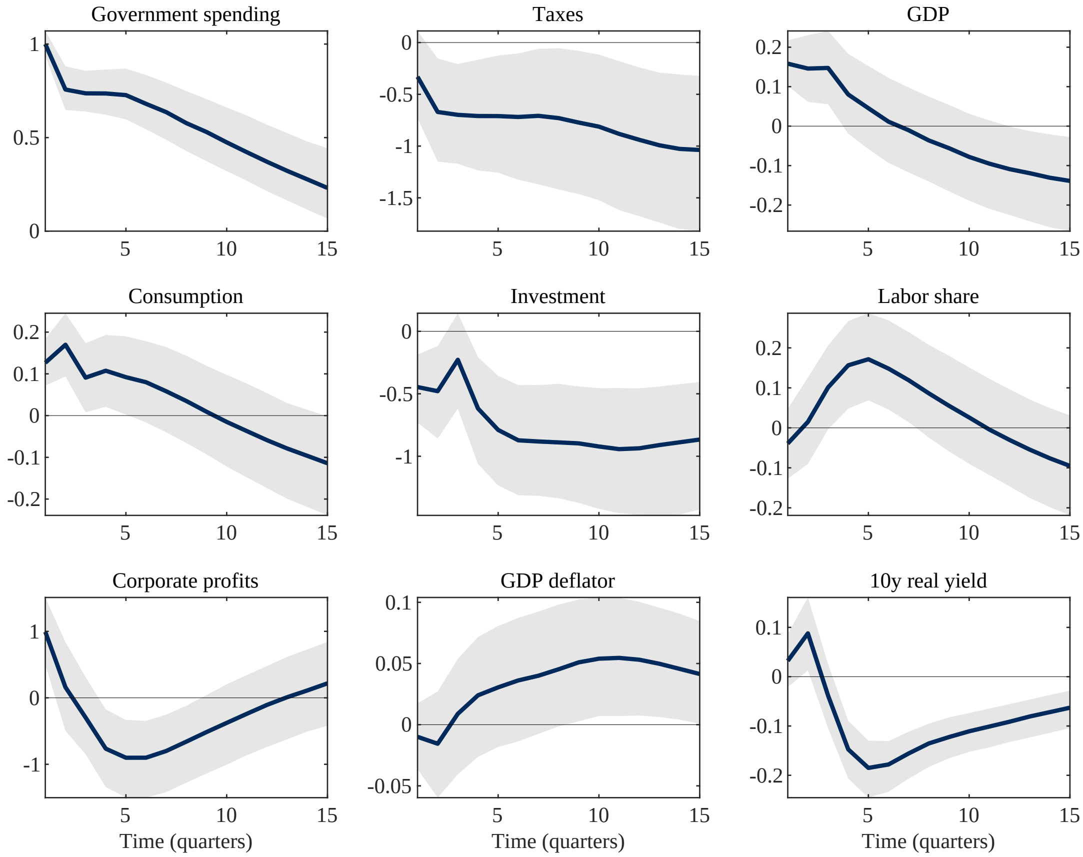

Figure D.1: VAR evidence on the effects of an unanticipated shock to government spending (U.S.)

Notes: This figure shows empirical impulse responses for an unanticipated government spending shock. Impulse responses are scaled such that the log change of government spending is unity at its peak. Solid lines indicate the median posterior density of impulse responses, while the shaded area represents the 16th to 84th percentiles. All series except interest rate and inflation rate shown in proportional deviations from baseline.

levels to preserve potential cointegrating relationships among the variables. We include a quadratic time trend as in Ramey (2016) to capture features such as the productivity slowdown or the effect of the baby boom. Results are robust to the inclusion of a linear trend (or a constant) only. In line with standard Bayesian practice, the (reduced-form) VAR is estimated using Markov Chain Monte Carlo Methods employing a normal-diffuse ("Jeffrey's") prior for the coefficient matrix and the covariance matrix of the reduced-form innovations, respectively. Impulse responses and posterior credible sets are generated based on 10,000 draws.

Figure D.1 reports the full set of impulse responses for a one percent increase in government spending. Notice that in the main text we considered a five times larger shock (approximately one

percent of GDP) in order to facilitate a more direct comparison with theoretical results coming from models with zero and positive government spending in steady-state, respectively. Three results stand out. Firstly, the response of real output is positive, but for only about one and a half years. The impact multiplier is 0.8, but reflecting the short-lived nature of the expansion, the present-value cumulative multiplier (Mountford and Uhlig (2009)) after two years is only 0.48; it falls to 0.16 after three years.[D.4] Secondly, and considering the components of national income, aggregate consumption is crowded *in* following an expansionary unanticipated government spending shock, whereas investment falls sharply (cf. Ramey (2016)).

Finally, and focusing on the novel aspect of our empirical exercise, the labor share exhibits a positive, persistent and hump-shaped response. The peak effect in percentage deviations from baseline is comparable to that of GDP, but the rise is gradual. In addition, the response is statistically significant for several quarters around its peak. This results suggests that the expansionary government spending shock induces a redistribution of national income away from recipients of capital income towards workers.[D.5]

D.2 Results for Australia, Canada, and the UK

The scope of the new stylized fact about the response of the labor share to government spending shocks is not restricted to the US, as this section demonstrates by examining the cases of Canada, Australia and the UK. In all three cases, we limit ourselves to recursive identification given limited data availability in terms of proxies for news shocks, and we study the sample 1970:I-2007:IV for which high-quality data is available for all three countries. We use two lags for Canada and Australia and three for the UK. Figure D.2 shows that in all three countries, in response to a surprise government spending shock, the labor share initially increases in a statistically significant manner

[D.4] The magnitude and (relatively low) persistence of the output response is consistent with other studies that noted the decline of the output effect after 1980 in U.S. data (see, e.g., Perotti (2005) and Caldara and Kamps (2008)).

[D.5] These cyclical shifts in the functional income distribution – with government spending shocks inducing redistribution from firm owners to workers – may help further motivate the use of a two-agent model that distinguishes, in a stylized fashion, between households primarily relying on labor income (workers) and those owning the economy's dividends-distributing firms (capitalists).

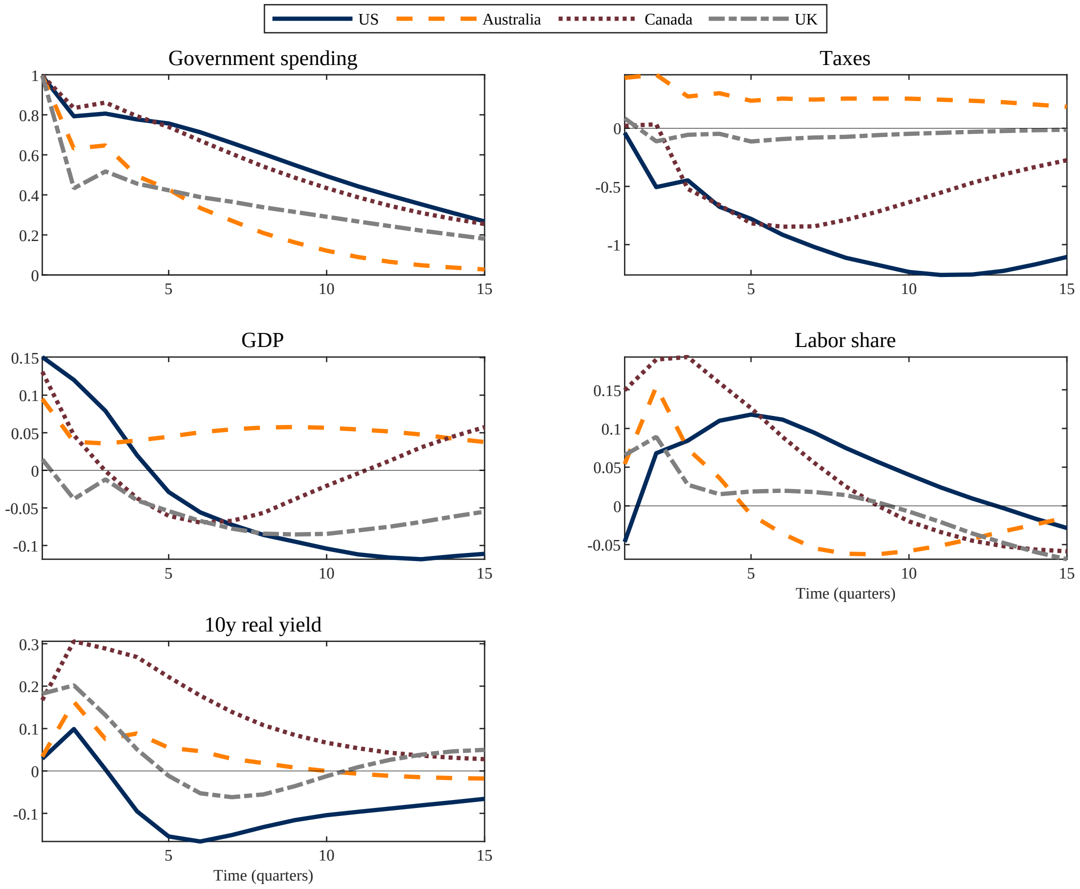

Figure D.2: VAR evidence on the effects of an unanticipated shock to government spending (Australia, Canada, UK)

Notes: This figure shows impulse responses for a recursively identified government spending shock, estimated separately for Australia, Canada, and the UK (1970:I-2007:IV). The U.S. case is shown for comparison purposes. It is identified and estimated using the same method over the same sample. The median posterior density of impulse responses is displayed in form of a solid line while the 16th and the 84th percentiles are shown as dotted lines. Impulse responses are scaled such the log change of government spending is unity at its peak. All series except interest rate shown in %.

before reverting back to the mean, potentially with a degree of undershooting after several years. Qualitatively, these dynamics are remarkably close to those reported earlier for the US.[D.6]

D.3 Further robustness checks

In the working paper version of this article (link), we report extensive robustness checks, e.g., Jordà's (2005) local projection method and varying sub-samples.

[D.6] The magnitude of the labor share increase for the Canada and Australia is notably larger than observed for the US, but it is significantly smaller for the UK where, in addition, the multiplier is negative (consistent with Afonso and Sousa (2012)).

D.4 Data Sources and Transformation

USA The components of national income, government receipts and the GDP deflator are taken from the NIPA tables of the Bureau of Economic Analysis. Further series are retrieved from the FRED database of the Federal Reserve Bank of St. Louis. All national income series are seasonally adjusted by the source and, unless otherwise stated, are deflated using the GDP deflator. Where necessary we take the arithmetic average of monthly figures to obtain quarterly series. Data from the Survey of Professional Forecasters is available on the website of the Federal Reserve Bank of Philadelphia.[D.7] Table D.1 lists the data sources used.

Our measure of the labor share considers the domestic non-financial corporate (NFC) sector. As is frequently done especially in sectoral studies, gross value added (GVA) is used. The formula is

$$LS = 1 - \frac{CP^{gva} + NI^{gva} - Tax^{gva}}{NVA}.$$

Canada, Australia and United Kingdom For Australia, Canada and the United Kingdom data are retrieved from the Australian Bureau of Statistics, Canada Statistics and the UK Office for National Statistics, respectively.[D.10] Table D.2 summarizes.

[D.7]The SPF provides separate forecasts for state, local and federal government spending, whereas our variable of interest is total government spending. We aggregate the individual components to obtain a forecast for the latter, and constructed news variables on this basis. This procedure may introduce bias in our estimates, because in 1996, the U.S. Bureau of Economic Analysis (BEA) switched its method for aggregating the headline components of real GDP and the associated price indexes from the fixed-weight aggregation method to the chain-weight aggregation method. Under the latter ("Fisher ideal"), additivity of real levels does not hold (Whelan 2002). We have verified that the results obtained are robust to using news variables based on federal spending only.

[D.8]Before 1953:II, interpolated annual data available on Robert Shiller's database at http://www.econ.yale.edu/ shiller/-data.htm

[D.9]http://econweb.ucsd.edu/ vramey/research.html

[D.10]Seasonally adjusted fiscal data for the UK going back to 1963 were were kindly provided by the ONS.

Mnemonic	Description	Source
GOV	Gov. consumption expenditures + gross investment	NIPA 1.1.5.
GOVCON	Gov. consumption expenditures	NIPA 3.9.5
GOVINV	Gov. gross investment	NIPA 3.9.5
TAX	Current receipts - current transfer payments - current interest payments	NIPA Table 3.1
GDP	Gross Domestic Product	NIPA 1.1.5
RINT	10Y Tsy constant maturity rate (quarterly avg.), adjusted by GDP deflator	FRED: GS10[D.8]
HOURS	Total hours worked, including military	V. Ramey's database[D.9]
WAGES	Real Hourly Compensation, Business Sector	BLS: PRS84006153
PGDP	GDP deflator	NIPA 1.1.4
LS1	LS in the non-farm business sector	BLS
LS2	LS in the non-financial business sector	BLS
CE	Compensation of employees	NIPA 1.12
CE_{gov}	Wages and salaries: government	NIPA 1.12
RI	Rental income (with CCAdj)	NIPA 1.12
CP	Corporate profits (with IVA and CCAdj)	NIPA 1.12
NI	Net interest income	NIPA 1.12
δ	Consumption of fixed capital	NIPA 1.7.5
PI	Proprietors' Income	NIPA 1.12
TAX^P	Taxes on production - subsidies on production	NIPA 1.12
BCTP	Business current transfer payments	NIPA 1.12
Sdis	Statistical discrepancy	NIPA 1.12
GE	Current surplus of government enterprises	NIPA 1.12
GNP	Gross national product	NIPA 1.7.5
CP^{gva}	Corporate profits, GVA (NFC)	NIPA 1.14
NI^{gva}	Net interest and miscellaneous payments (NFC)	NIPA 1.14
TAX^{gva}	Taxes less subsidies on production and imports (NFC)	NIPA 1.14
NVA	Net value added (NFC)	NIPA 1.14

Table D.1: Data Sources – US

Mnemonic	Australia	Canada	UK
GOV	General government final consumption expenditure + general government gross fixed capital formation	General government final consumption expenditure + general governments gross fixed capital formation	General government total current expenditure + total net investment
TAX	General government total gross income - general government total income payable - subsidies	General government revenue - current transfers to households - interest on debt	General government total current receipts - net social benefits
GDP	GDP adjusted using the GDP deflator	GDP adjusted using the GDP deflator	GDP adjusted using the GDP deflator
LS	Naive measure calculated as total wages and salaries (including social security contributions) over GDP	Naive measure calculated as compensation of employees over total factor income, computed as (GDP-taxes less subsidies on products and imports)	Naive measure calculated as compensation of employees over gross value added at factor cost
RINT	10 year government bond yield (FRED: IRLTLT01AUQ156N) deflated using the GDP deflator	10 year government bond yield (FRED: IRLTLT01CAM156N) deflated using the GDP deflator	10 year government bond yield (FRED: IRLTLT01GBM156N) deflated using the GDP deflator

Table D.2: Data Sources – Australia, Canada, and UK

Appendix E Estimation through IRF matching

This section reports the results of estimating the different medium-scale TANK models using IRF matching techniques. Our primary interest is to examine whether the medium scale TANK-CW model presented in section B.3 is able to match the empirical impulse responses to a government spending shock; and, if so, for what parameter alues.

Methodology. We follow Christiano *et al.* (2010) and estimate the three models using Bayesian impulse response matching (also see, among others, Christiano *et al.* (2016) and Lewis and Winkler (2017)). This technique consists in estimating a selected number of parameters in the model by minimizing the distance between the SVAR- and the theoretical IRFs of interest.[E.1] From the SVAR in figure D.1 we match 6 of the 9 IRFs. We leave out the three variables for which we do not have a clear counterpart in the DSGE models. These are the SPF variable, total tax revenues and corporate profits. The reason why we cannot match the first one is obvious given the assumption of rational expectations in the models. Total tax revenues include distortionary taxes and transfers while we assume only lump sum taxes and transfers in the model. Regarding profits the argument is twofold. First is not obvious that data on real corporate profits are consistent with economic profits in the models and second, in the models but not in the data, profits are the mirror image of the labor share.

For the estimation we partition the model's parameters into two groups. The first group comprises parameters for which there exist conventional values in the literature (see Table B.4). The values of the discount factor ($\beta = 0.99$) and of the capital depreciation rate ($\delta = 0.025$) are standard for models calibrated at a quarterly frequency. We set the Frisch elasticity of labor supply equal to unity ($\varphi = 1$). Intertemporal elasticities of substitution in the goods and labor market ($\eta = \eta^w = 6$) are set in order to match average mark-ups in the product and labor markets equal to 1.2. In line with historical U.S. data, at the steady state, we set a government spending share of output of 20% ($g/y = 0.20$). The gross inflation rate ($\Pi = 1$) implies a zero-inflation steady state, while the steady-state labor supply is set equal to 1/3 of the available time ($n = 0.33$). Government debt is set to 57% of annual

[E.1] We refer the interested reader to Christiano *et al.* (2010) for a detailed technical discussion of the minimum distance estimator used here.

output ($\frac{b}{y} = 4 \times 0.57$), which corresponds to the average value of the U.S. government debt to GDP ratio during the great moderation. Workers' steady-state bond holdings and benchmark level for the portfolio adjustments costs (b^W) are set equal to zero, as in the main text. Finally, in line with the bulk of the TANK literature, steady-state lump sum transfers/taxes are set such that there is no steady-state consumption inequality, since in this paper we are only interested in deviations from steady state.

The second group of parameters is estimated such as to minimize the distance between the SVAR responses and the model's responses of six key variables: government spending, GDP, the labor share, private consumption, investment and inflation. Table E.1 shows the choice of prior distributions. We use a Gamma distribution for the standard deviation of the government spending shock and a Beta distribution for the autoregressive parameter. For the percentage of hand-to-mouth/workers in the economy we use a Normal distribution centered around 0.5. The prior distribution for ψ^W is a normal centered around 0.2265, a value chosen following the same rationale applied in calibrating the simple model and that matches both the impact quarterly and annual iMPCs.[E.2] Furthermore, to estimate the Rotemberg price and wage rigidity parameters we use use the following approach. We estimate directly the slope of the price and wage Phillips curves, respectively $\kappa = \frac{\eta-1}{\xi}$ and $\kappa^w = \frac{\eta^w-1}{\xi^w}$, and then use the calibration of η and η^w to back out the price/wage adjustment costs. For κ and κ^w we use a Beta centered around 0.1. A Gamma distribution centered around usual values found in the literature is also used for investment adjustment costs, and the response to inflation and output in the Taylor rule. Lastly, we use a Beta distribution for interest rate smoothing.

Results. Figure E.1 and Table E.1 document the results. Three observations stand out. First, all three model variants can fit the aggregate data reasonably well in sample, with the exception of sluggish inflation dynamics and, for the TANK-UH model perhaps the persistence of the negative response of investment. Second, while the posterior means for common parameters between the three models are estimated to be very close, the results for the population share λ are notable. In

[E.2]Note however that this matching to micro evidence is only valid at the prior mean and that both parameters will be estimated separately.

particular, to fit the macro data the TANK-UH model requires 60% of the population not to have any access to financial markets and behave in a hand-to-mouth way. As such, while the model can, in principle, replicate empirical impulse responses, it does so at the expenses of implying a fraction of hand to mouth agents in the economy that is very far from empirical estimates (see Kaplan and Violante (2014)).

Finally, and concentrating on the models with workers, consider the estimated values of λ and ψ^W. Our baseline calibration approach targeted evidence on intertemporal marginal propensities to consume from *micro, cross-sectional* data. Matching median moments for the fraction consumed out of an unanticipated income windfall after one quarter and one year, we set λ equal to 0.8 and ψ^W equal to 0.074. In the estimation on *macro, time-series* data, even when imposing a prior on λ that is centered around 0.5, the share of workers in the UW and CW models is likewise found to be 0.8 and 0.75, respectively. At 0.21 (in UW) and 0.15 (in CW), the portfolio adjustment cost parameter, ψ^W is estimated to be slightly higher than implied by our partial equilibrium matching exercise on micro data, but given a prior centered on 0.0227 the posterior mean is likewise pushed in the 'right' direction and the 95% HPD interval for TANK-CW include the value 0.074. As such, estimation results from both macro and micro data point towards similar values of the key parameters λ and ψ^W in two-agent models with workers.

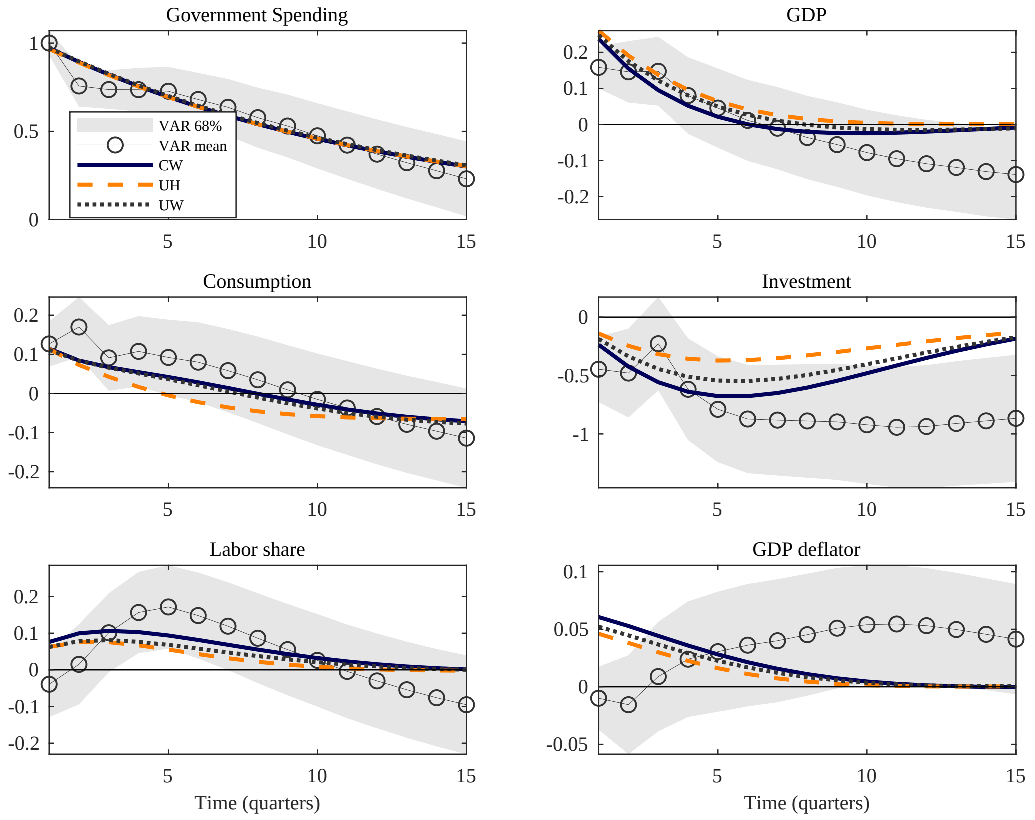

Figure E.1: Bayesian impulse response matching: model fit

Notes: This figure shows the fit of the estimated TANK models relative to the empirical, VAR-based impulse response for an unanticipated one percent increase in government spending. The dotted line indicates the median posterior density of empirical impulse responses, with shaded area representing the 16th to 84th percentiles. All series except the GDP deflator shown in %.

Description	Parameter	Prior	Posterior mean (95% HDP interval)		
			UH	UW	CW
G shock	ε^G	$\Gamma(1, 0.05)$	0.97 (0.91, 1.03)	0.97 (0.91, 1.03)	0.97 (0.91, 1.03)
AR1 government spending shock	ρ^G	$B(0.7, 0.15)$	0.92 (0.90, 0.94)	0.92 (0.90, 0.94)	0.92 (0.90, 0.94)
Inv. adj. costs	ι	$\Gamma(4, 2)$	2.61 (0.89, 4.24)	2.30 (0.86, 3.69)	1.96 (0.79, 3.14)
Slope of Phillips curve	κ	$B(0.1, 0.02)$	0.08 (0.05, 0.10)	0.08 (0.05, 0.10)	0.07 (0.05, 0.10)
Slope of wage Phillips curve	κ^w	$B(0.1, 0.02)$	0.10 (0.06, 0.13)	0.10 (0.06, 0.13)	0.09 (0.06, 0.12)
% of H/W	λ	$N(0.5, 0.15)$	0.61 (0.53, 0.69)	0.80 (0.68, 0.93)	0.75 (0.59, 0.90)
Portfolio adj. costs	ψ^W	$N(0.227, 0.1)$	∞	0.21 (0.08, 0.33)	0.15 (0.05, 0.25)
Interest rate smoothing	ϕ^r	$B(0.7, 0.2)$	0.55 (0.31, 0.79)	0.65 (0.44, 0.86)	0.64 (0.44, 0.85)
Interest rate response to inflation Π	ϕ^π	$\Gamma(1.7, 0.15)$	1.71 (1.46, 1.95)	1.71 (1.46, 1.94)	1.72 (1.48, 1.95)
Interest rate response to output	ϕ^y	$\Gamma(0.1, 0.05)$	0.10 (0.03, 0.17)	0.10 (0.03, 0.17)	0.10 (0.03, 0.18)

Table E.1: Bayesian impulse response matching: parameter estimates

Notes: This table summarizes prior and posterior distributions for the set of parameter values estimated according to IRF matching. Distributions are abbreviated as follows: Γ - Gamma; B - Beta; N - Normal.

References for online appendix

Afonso, A. and Sousa, R. M. (2012). The Macroeconomic Effects of Fiscal Policy. *Applied Economics*, **44**(34), 4439–4454.

Altig, D., Christiano, L., Eichenbaum, M., and Lindé, J. (2011). Firm-Specific Capital, Nominal Rigidities and the Business Cycle. *Review of Economic Dynamics*, **14**(2), 225–247.

Attanasio, O., Levell, P., Low, H., and Sánchez-Marcos, V. (2018). Aggregating Elasticities: Intensive and Extensive Margins of Women's Labor Supply. *Econometrica*, **86**(6), 2049–2082.

Auclert, A., Rognlie, M., and Straub, L. (2018). The Intertemporal Keynesian Cross. Working Paper 25020, National Bureau of Economic Research.

Auclert, A., Rognlie, M., and Straub, L. (2020a). Micro Jumps, Macro Humps: Monetary Policy and Business Cycles in an Estimated HANK Model. *In Preparation*.

Auclert, A., Bardóczy, B., and Rognlie, M. (2020b). MPCs, MPEs, and Multipliers: A Trilemma for New Keynesian Models. *mimeo*.

Auerbach, A. J. and Gorodnichenko, Y. (2012). Measuring the Output Responses to Fiscal Policy. *American Economic Journal: Economic Policy*, **4**(2), 1–27.

Ben Zeev, N. and Pappa, E. (2015). Multipliers of Unexpected Increases in Defense Spending: An Empirical Investigation. *Journal of Economic Dynamics and Control*, **57**, 205–226.

Bilbiie, F. (2008). Limited Asset Markets Participation, Monetary Policy and (Inverted) Aggregate Demand Logic. *Journal of Economic Theory*, **140**(1), 162–196.

Bilbiie, F. O. (2019). The New Keynesian Cross. *Journal of Monetary Economics*.

Blanchard, O. and Perotti, R. (2002). An Empirical Characterization of the Dynamic Effects of Changes in Government Spending and Taxes on Output. *The Quarterly Journal of Economics*, **117**(4), 1329–1368.

Born, B., Juessen, F., and Müller, G. J. (2013). Exchange Rate Regimes and Fiscal Multipliers. *Journal of Economic Dynamics and Control*, **37**(2), 446–465.

Caldara, D. and Kamps, C. (2008). What are the Effects of Fiscal Policy Shocks? A VAR-based Comparative Analysis.

Caldara, D. and Kamps, C. (2017). The Analytics of SVARs: A Unified Framework to Measure Fiscal Multipliers. *The Review of Economic Studies*.

Campbell, J. Y. and Mankiw, N. G. (1989). Consumption, Income and Interest Rates: Reinterpreting the Time Series Evidence. *NBER Macroeconomics Annual 1989, Volume 4*, pages 185–246.

Carroll, C. D., Crawley, E., Slacalek, J., Tokuoka, K., and White, M. N. (2018). Sticky Expectations and Consumption Dynamics. Working Paper 24377, National Bureau of Economic Research.

Chetty, R., Guren, A., Manoli, D., and Weber, A. (2013). Does Indivisible Labor Explain the Difference between Micro and Macro Elasticities? A Meta-analysis of Extensive Margin Elasticities. *NBER Macroeconomics Annual*, **27**(1), 1–56.

Christiano, L. J., Trabandt, M., and Walentin, K. (2010). DSGE Models for Monetary Policy Analysis. In B. M. Friedman and M. Woodford, editors, *Handbook of Monetary Economics*, volume 3 of *Handbook of Monetary Economics*, chapter 7, pages 285–367. Elsevier.

Christiano, L. J., Eichenbaum, M. S., and Trabandt, M. (2016). Unemployment and Business Cycles. *Econometrica*, **84**(4), 1523–1569.

Forni, M. and Gambetti, L. (2016). Government Spending Shocks in Open Economy VARs. *Journal of International Economics*, **99**(C), 68–84.

Gali, J., Lopez-Salido, J. D., and Valles, J. (2004). Rule-of-Thumb Consumers and the Design of Interest Rate Rules. *Journal of Money, Credit, and Banking*, **36**(4), 739–763.

Galí, J., López-Salido, J. D., and Vallés, J. (2007). Understanding the Effects of Government Spending on Consumption. *Journal of the European Economic Association*, **5**(1), 227–270.

Gomme, P. and Rupert, P. (2004). Measuring Labor's Share of Income. SSRN Scholarly Paper, Social Science Research Network, Rochester, NY.

Gutierrez, G. and Piton, S. (2019). Revisiting the Global Decline of the (Non-Housing) Labor Share. Technical Report 1913, Centre for Macroeconomics (CFM).

Hagedorn, M. (2018). A Demand Theory of the Price Level. *mimeo*.

Havranek, T., Rusnák, M., and Sokolova, A. (2017). Habit Formation in Consumption: A Meta-analysis. *European Economic Review*, **95**(C), 142–167.

Jordà, O. (2005). Estimation and Inference of Impulse Responses by Local Projections. *The American Economic Review*, **95**(1), 161–182.

Kaplan, G. and Violante, G. L. (2014). A Model of the Consumption Response to Fiscal Stimulus Payments. *Econometrica*, **82**(4), 1199–1239.

Leeper, E. M., Walker, T. B., and Yang, S.-C. S. (2013). Fiscal Foresight and Information Flows. *Econometrica*, **81**(3), 1115–1145.

Lewis, V. and Winkler, R. (2017). Government Spending, Entry, and the Consumption Crowding-In Puzzle. *International Economic Review*, **58**(3), 943–972.

Maliar, L. and Naubert, C. (2019). Tank Models with Amplification and No Puzzles: The Magic of Output Stabilization and Capital. SSRN Scholarly Paper ID 3496649, Social Science Research Network, Rochester, NY.

Michaillat, P. and Saez, E. (2019). Resolving New Keynesian Anomalies with Wealth in the Utility Function. *The Review of Economics and Statistics*, pages 1–46.

Mountford, A. and Uhlig, H. (2009). What are the effects of fiscal policy shocks? *Journal of Applied Econometrics*, **24**(6), 960–992.

Neumeyer, P. A. and Perri, F. (2005). Business Cycles in Emerging Economies: The Role of Interest Rates. *Journal of Monetary Economics*, **52**(2), 345–380.

Perotti, R. (2005). Estimating the Effects of Fiscal Policy in OECD Countries. SSRN Scholarly Paper ID 717561, Social Science Research Network, Rochester, NY.

Ramey, V. and Zubairy, S. (2018). Government Spending Multipliers in Good Times and in Bad: Evidence from U.S. Historical Data. *Journal of Political Economy*, **126**(2).

Ramey, V. A. (2011). Identifying Government Spending Shocks: It's all in the Timing. *The Quarterly Journal of Economics*, **126**(1), 1–50.

Ramey, V. A. (2016). Macroeconomic Shocks and their Propagation. *Handbook of Macroeconomics*, **2**, 71–162.

Ricco, G. (2015). A New Identification of Fiscal Shocks Based on the Information Flow. SSRN Scholarly Paper ID 2621656, Social Science Research Network, Rochester, NY.

Rognlie, M. (2018). Comment on "Accounting for Factorless Income". In *NBER Chapters*, pages 235–248. National Bureau of Economic Research, Inc.

Schmitt-Grohe, S. and Uribe, M. (2003). Closing Small Open Economy Models. *Journal of International Economics*, **61**(1), 163–185.

Smets, F. and Wouters, R. (2007). Shocks and Frictions in US Business Cycles: A Bayesian DSGE Approach. *American Economic Review*, **97**(3), 586–606.

Whelan, K. (2002). A Guide To U.S. Chain Aggregated Nipa Data. *Review of Income and Wealth*, **48**(2), 217–233.

Yang, S. S.-C. (2005). Quantifying Tax Effects under Policy Foresight. *Journal of Monetary Economics*, **52**(8), 1557–1568.
