# Supplementary material for: Workers, capitalists, and the government: fiscal policy and income (re)distribution
Source: J Monet Econ. 2021 Apr;119:58–74. doi: 10.1016/j.jmoneco.2021.01.004 (PMC8100925; doi:10.1016/j.jmoneco.2021.01.004)
Supplement: Supplementary Data S2 — Supplementary Raw Research Data. This is open data under the CC BY license http://creativecommons.org/licenses/by/4.0/ [file mmc2.zip › TANK-CW_Replication-main/CF_JME_Workers, Capitalists, Government_Documentation for Replication.pdf]

Replication files for:

"Workers, Capitalists, and the Government: Fiscal Policy and Income (Re)Distribution"

Cristiano Cantore[†] Lukas B. Freund[‡]

January 2021

This document describes how to use the replication codes accompanying the article "Workers, Capitalists, and the Government: Fiscal Policy and Income (Re)Distribution." Corresponding to sections 2 and 3 of the paper, there are two main folders that respectively consider the partial equilibrium consumption-savings problem and the proposed, new two-agent New Keynesian (TANK) model with capitalists and workers.

Should you have any questions or comments, please feel free to contact us under the indicated email addresses.

1 Partial equilibrium iMPCs

1.1 Description of main files

The files contained in this folder are as follows.

`main_PE_MPCs_Analytical_Plotting.m`

This file computes and plots iMPCs for a one-off income shock in a partial equilibrium consumption-savings model with portfolio adjustment costs, using the analytical solution (accurate to first-order)

[†]Bank of England, CfM & University of Surrey. Email: cristiano.cantore@gmail.com. Web: cristianocantore.com.
[‡]University of Cambridge. Email: lukas.beat.freund@gmail.com. Web: lukasfreund.com.

provided in the main text. The user can choose the anticipation horizon s. The structural parameters are calibrated to match the empirical evidence, as described in the main text.

`main_PE_MPCs_Analytical_Matching.m`

This file implements computes the values of population share λ and portfolio adjustment cost parameter ψ to match targets from micro consumption data.

`main_PE_MPCs_LTI.m`

Complementing the analytical solution, this file solves the partial equilibrium model numerically. We use the linear time iteration approach of Rendahl (2017). We also used this approach to compute the iMPCs in the model with habits (see parameter 'sPar.hab').

`main_PE_Rates_Analytical_Plotting.m`

This file is similar to `main_PE_MPCs_Analytical_Plotting.m`, the only difference being that it looks at interest rate shocks.

`main_PE_Rates_Analytical_Extra.m`

This file plots some additional figures, such as the interest rate elasticity of consumption as a function of ψ.

`sSettings.mat`

This structure object stores generic, design-related settings to ensure consistency across figures.

1.2 Exact replication of figures in the paper

Figure 2a. Run `main_PE_MPCs_Analytical_Plotting.m` with `s=0` and `optionModel` set to `H`.

Figure 2b. Run `main_PE_MPCs_Analytical_Plotting.m` with `s=0` and `optionModel` set to `W`.

Figure 3a. Run `main_PE_MPCs_Analytical_Plotting.m` with `s=3` and `optionModel` set to `H`.

Figure 3b. Run `main_PE_MPCs_Analytical_Plotting.m` with `s=3` and `optionModel` set to `W`.

2 TANK models

2.1 Description of main files

The sub-folder `Dynaremastercodes` contains Dynare implementations (Adjemian *et al.*, 2018) of the different TANK models. The remaining sub-folders contain files to reproduce the exact figures shown in the main text. Regarding the former, the files contained in this sub-folder are as follows.

Baseline models.

`tank_uh.mod`

Dynare code to replicate the baseline TANK-UH model linearized around 0 steady-state government spending/debt.

`tank_uw.mod`

Dynare code to replicate the baseline TANK-UW model linearized around 0 steady-state government spending/debt.

`tank_cw.mod`

Dynare code to replicate the baseline TANK-CW model linearized around 0 steady-state government spending/debt.

Medium-scale models.

`tank_cw_ms.mod`

Dynare code to replicate the medium scale version of the TANK models used in the paper. The file includes a pre-processor macro variable at the top that can be set to choose between different version of the model, i.e., RANK, TANK-UH, TANK-UW, TANK-CH and TANK-CW.

2.2 Exact replication of figures in the paper

Figure 5. Run `~TANKmodels/fig5/run_fig5.m`.

Figure 6a. Run `~TANKmodels/fig6/figure6a/run_fig6a.m`.

Figure 6b. Run ~TANKmodels/fig6/figure6a/run_fig6a.m.

Figure 7. Run ~TANKmodels/fig7/run_fig7.m.

Table 3. Run ~TANKmodels/table3/simplemodels/run_table3.m for the first four columns and ~TANKmodels/table3/mediumscale/run_table3MS.m for the last four columns.

3 Additional materials

3.1 Empirical evidence

Figure 1. Figure 1a relies on data from Fagereng *et al.* (2018), analyzed in and kindly shared by Auclert *et al.* (2018). Figure 1b replicates Figure 1 in Jappelli and Pistaferri (2014) using data distributed through openICPSR. We therefore do not include these files in this replication kit.

Figure 4. Data and code for this figure are in the sub-folder VAR. To reproduce the figure, run VAR_Plotting.m. That same file also permits reproducing the VAR-based figures shown in the online appendix.[1]

3.2 Online appendix

This folder contains various sub-folders with codes that replicate the figures in the online appendix.

References for documentation

Adjemian, S., Bastani, H., Juillard, M., Karamé, F., Maih, J., Mihoubi, F., Perendia, G., Pfeifer, J., Ratto, M., and Villemot, S. (2018). Dynare: Reference Manual Version 4. Technical Report 1, CEPREMAP.

[1]If you downloaded the replication files from the JME's website, some intermediate data (i.e., .mat files storing impulse responses) may not be included in the distribution due to size constraints. You can download VAR_IRFs_F14L2C3S6LS7_Surprise_Large_10Variables.mat, VarIrf_AusCanUK.mat and VarIrf_USComparison.mat either under this link (~Empiricalevidence\VAR\Data) or compute them by first running VAR_Baseline.m and VAR_OtherCountries.m.

Auclert, A., Rognlie, M., and Straub, L. (2018). The Intertemporal Keynesian Cross. Working Paper 25020, National Bureau of Economic Research.

Fagereng, A., Holm, M. B., and Natvik, G. J. J. (2018). MPC Heterogeneity and Household Balance Sheets. SSRN Scholarly Paper ID 2861053, Social Science Research Network, Rochester, NY.

Jappelli, T. and Pistaferri, L. (2014). Fiscal Policy and MPC Heterogeneity. *American Economic Journal: Macroeconomics*, **6**(4), 107–136.

Rendahl, P. (2017). Linear Time Iteration. *mimeo*.
