# Supplementary material for: Workers, capitalists, and the government: fiscal policy and income (re)distribution
Source: J Monet Econ. 2021 Apr;119:58–74. doi: 10.1016/j.jmoneco.2021.01.004 (PMC8100925; doi:10.1016/j.jmoneco.2021.01.004)
Supplement: Supplementary Data S2 — Supplementary Raw Research Data. This is open data under the CC BY license http://creativecommons.org/licenses/by/4.0/ [file mmc2.zip › TANK-CW_Replication-main/Online appendix/Appendix B/B2-4/Output/fig_sensi_UW_lambda_linear.pdf]

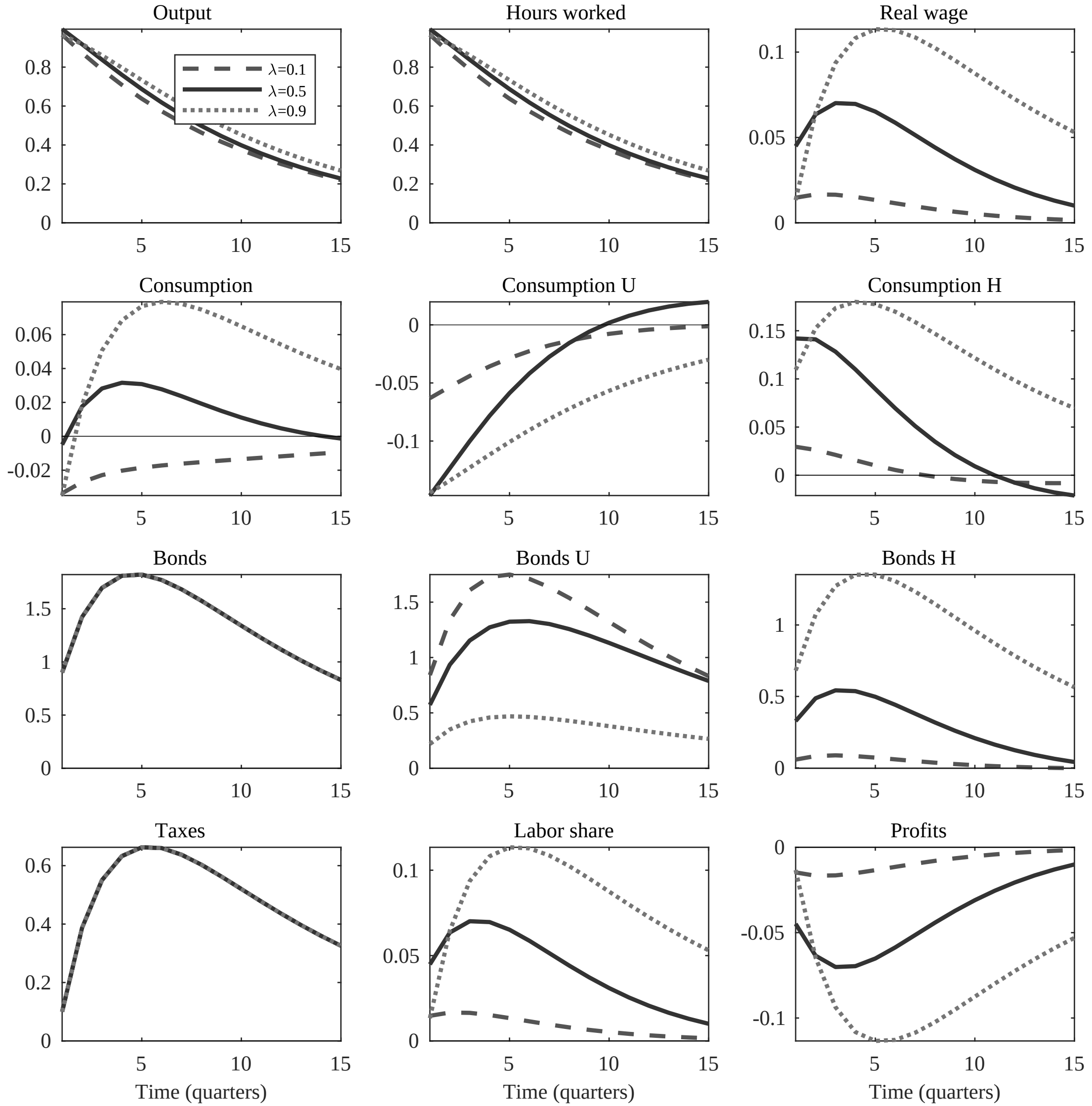
Output
Hours worked
Real wage
λ=0.1
λ=0.5
λ=0.9
Consumption
Consumption U
Consumption H
Bonds
Bonds U
Bonds H
Taxes
Labor share
Profits
Time (quarters)
Time (quarters)
Time (quarters)
