# Supplementary figures and images for: Workers, capitalists, and the government: fiscal policy and income (re)distribution
Source: J Monet Econ. 2021 Apr;119:58–74. doi: 10.1016/j.jmoneco.2021.01.004 (PMC8100925; doi:10.1016/j.jmoneco.2021.01.004)

GDP

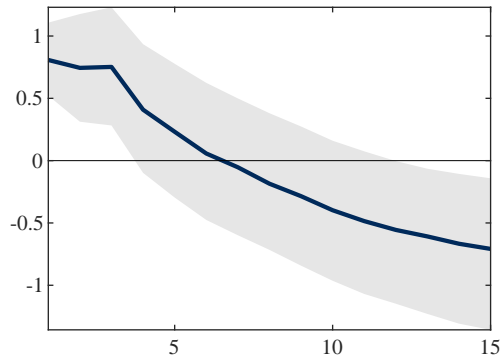

Consumption

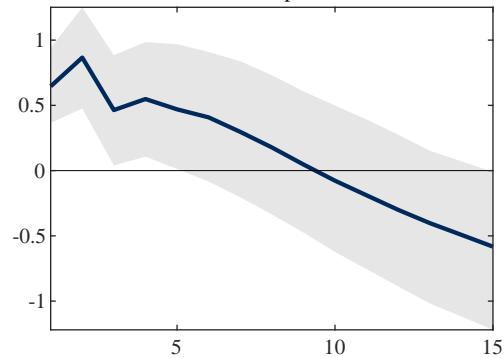

Investment

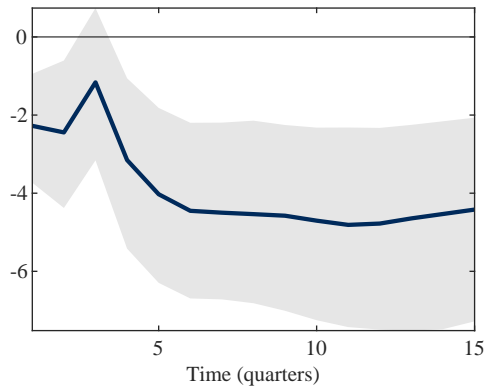

Labor share

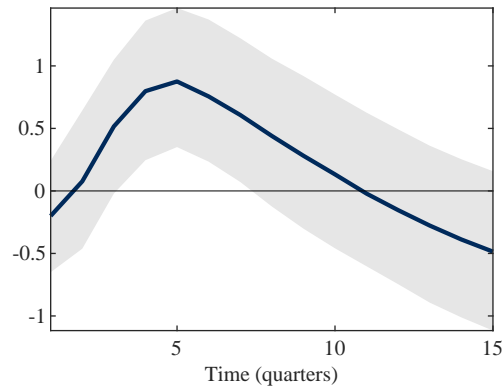

Supplement: Supplementary Data S2 — Supplementary Raw Research Data. This is open data under the CC BY license http://creativecommons.org/licenses/by/4.0/ [file mmc2.zip › TANK-CW_Replication-main/Empirical evidence/VAR/Output/fig_VAR_Baseline.pdf]

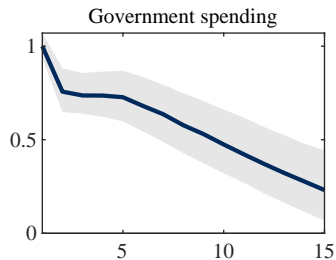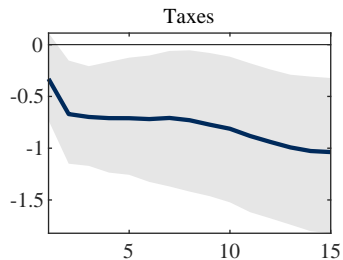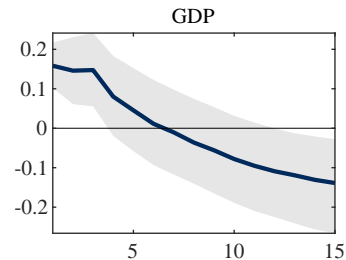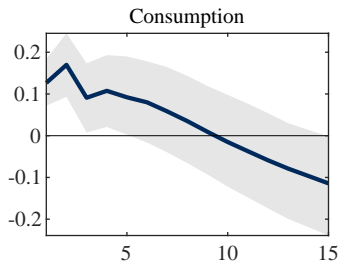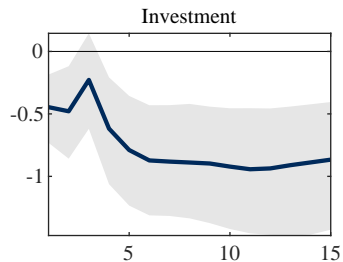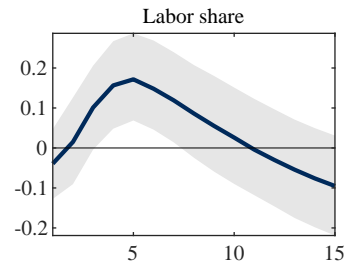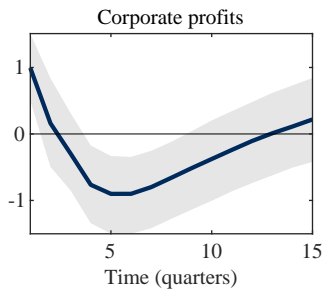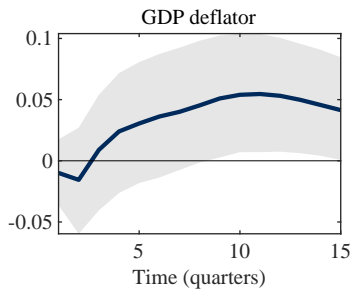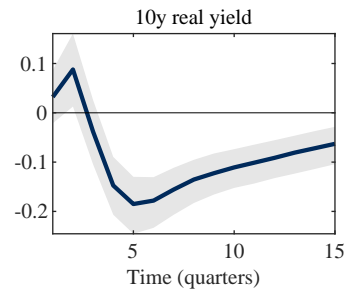

Supplement: Supplementary Data S2 — Supplementary Raw Research Data. This is open data under the CC BY license http://creativecommons.org/licenses/by/4.0/ [file mmc2.zip › TANK-CW_Replication-main/Empirical evidence/VAR/Output/fig_VAR_Benchmark_10V.pdf]

— US — Australia ··· Canada — UK

Government spending

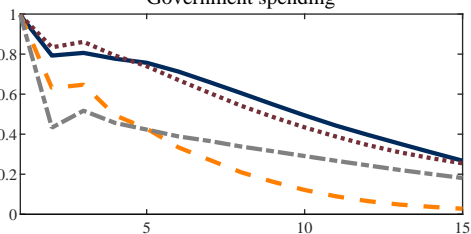

Taxes

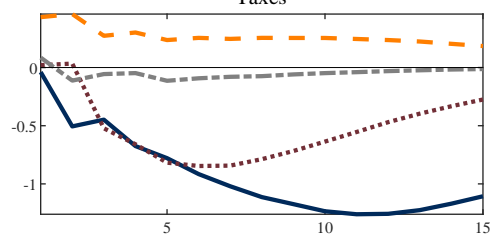

GDP

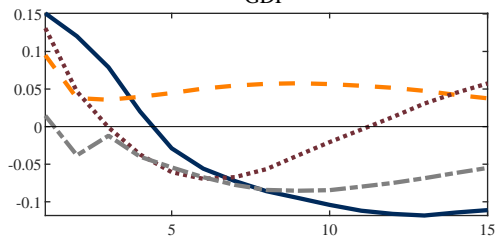

Labor share

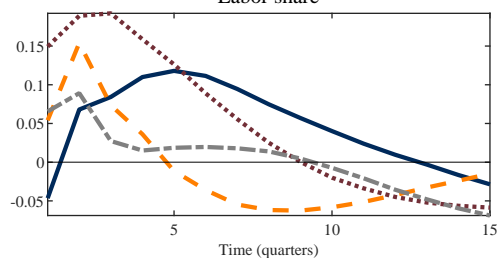

10y real yield

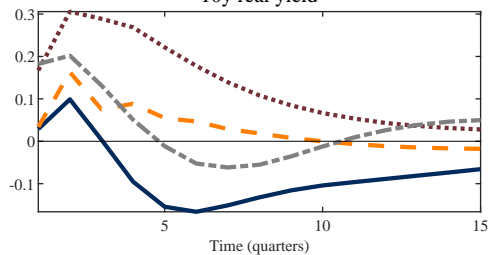

Supplement: Supplementary Data S2 — Supplementary Raw Research Data. This is open data under the CC BY license http://creativecommons.org/licenses/by/4.0/ [file mmc2.zip › TANK-CW_Replication-main/Empirical evidence/VAR/Output/fig_VAR_Comparison_Countries.pdf]

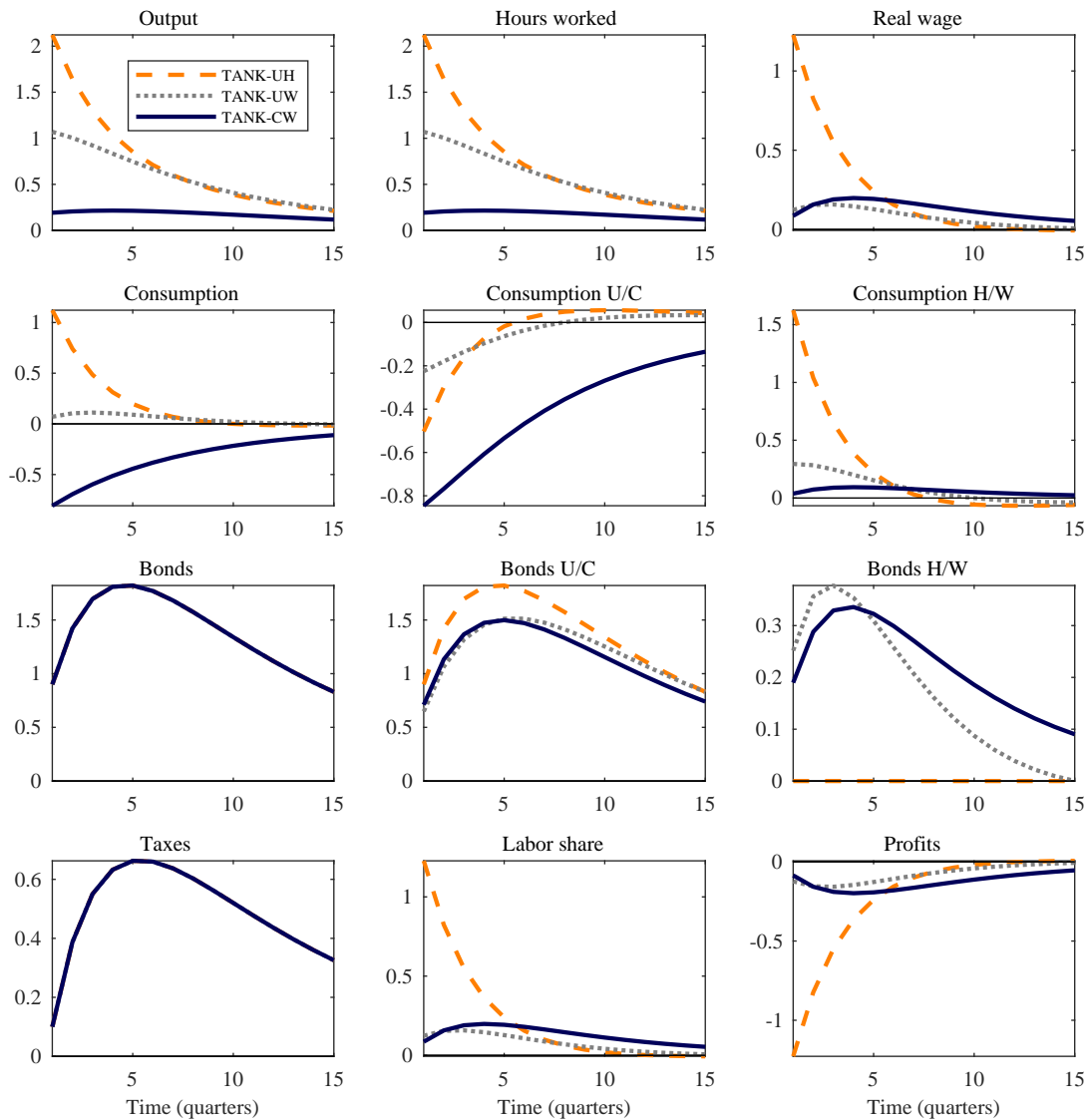

Supplement: Supplementary Data S2 — Supplementary Raw Research Data. This is open data under the CC BY license http://creativecommons.org/licenses/by/4.0/ [file mmc2.zip › TANK-CW_Replication-main/Online appendix/Appendix B/B1/Output/figB1.pdf]

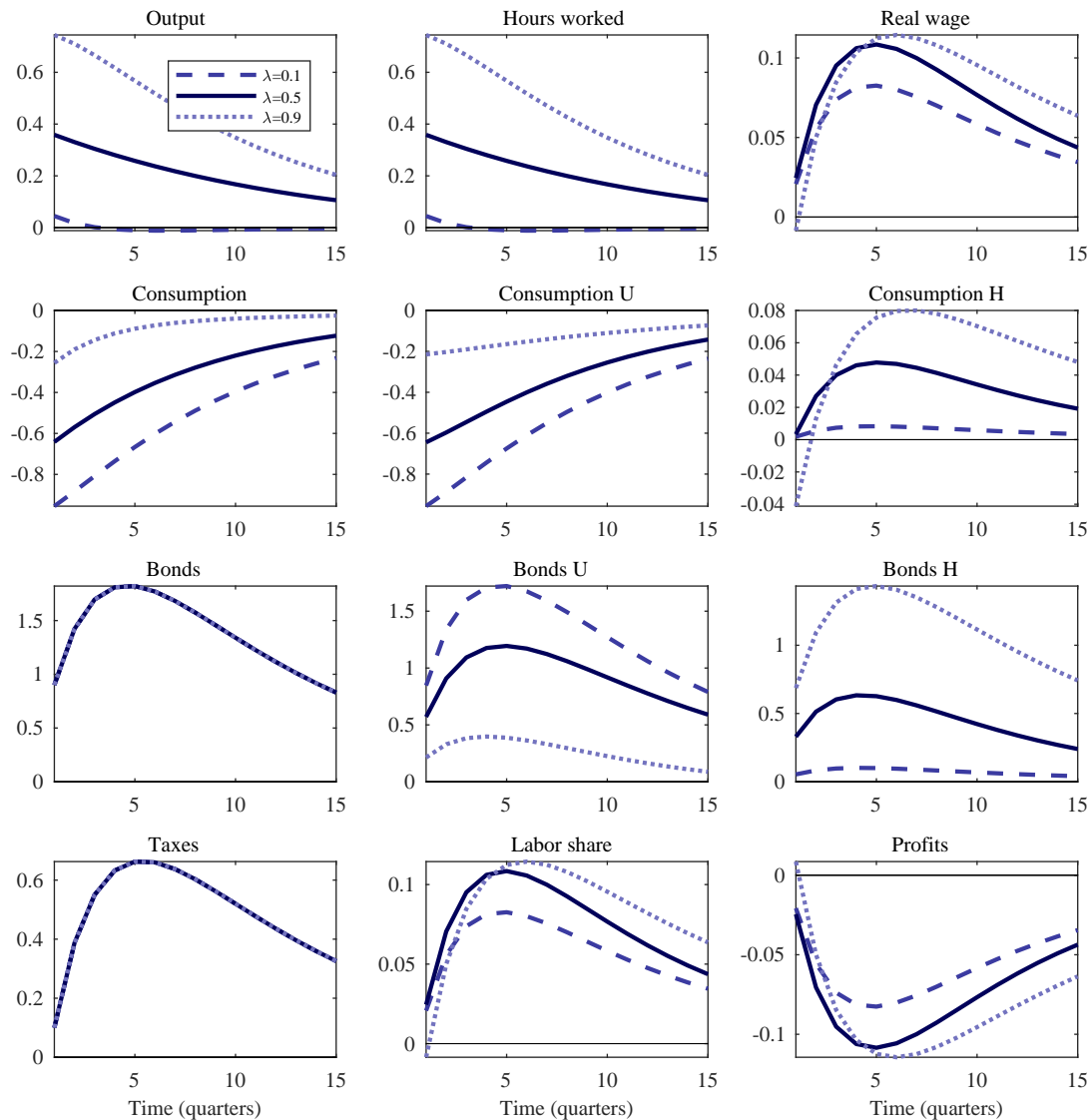

Supplement: Supplementary Data S2 — Supplementary Raw Research Data. This is open data under the CC BY license http://creativecommons.org/licenses/by/4.0/ [file mmc2.zip › TANK-CW_Replication-main/Online appendix/Appendix B/B2-4/Output/fig_sensi_CW_lambda_linear.pdf]

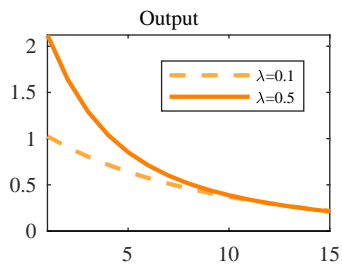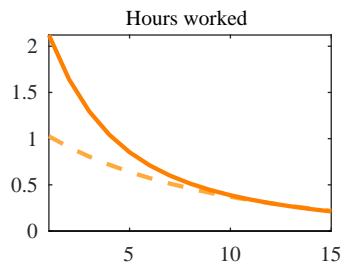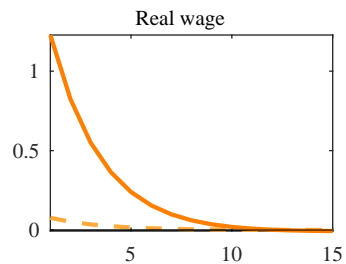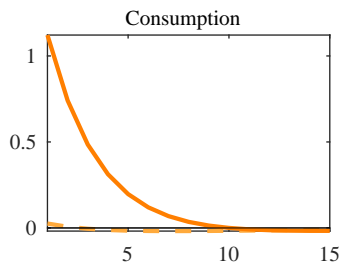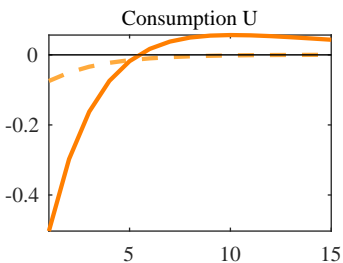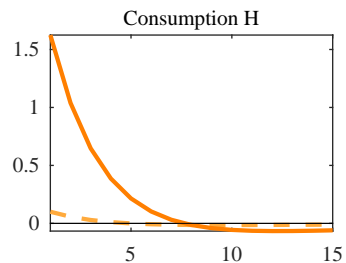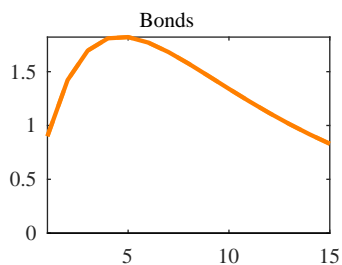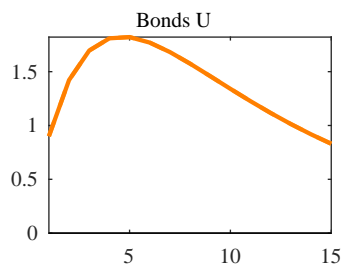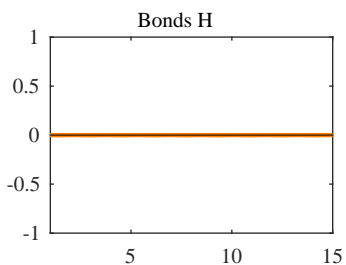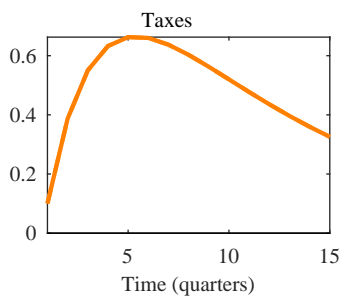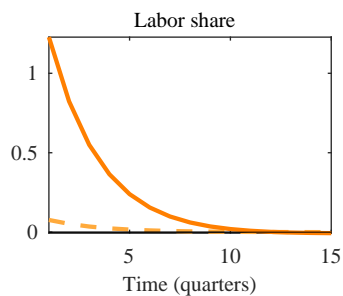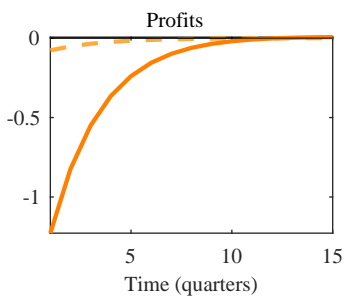

Supplement: Supplementary Data S2 — Supplementary Raw Research Data. This is open data under the CC BY license http://creativecommons.org/licenses/by/4.0/ [file mmc2.zip › TANK-CW_Replication-main/Online appendix/Appendix B/B2-4/Output/fig_sensi_UH_lambda_linear.pdf]

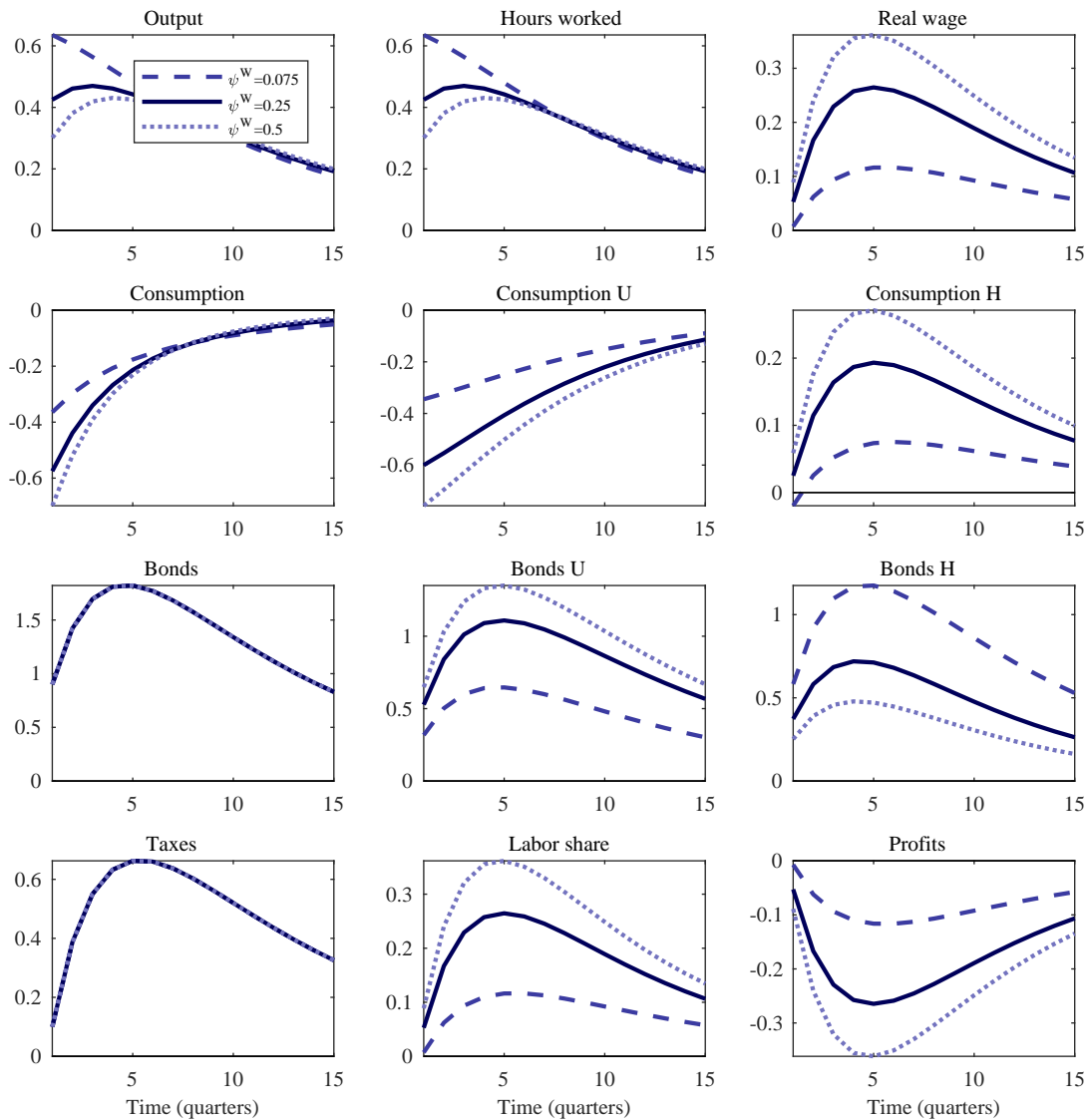

Supplement: Supplementary Data S2 — Supplementary Raw Research Data. This is open data under the CC BY license http://creativecommons.org/licenses/by/4.0/ [file mmc2.zip › TANK-CW_Replication-main/Online appendix/Appendix B/B5-6/Output/fig_sensi_CW_psiw_linear.pdf]

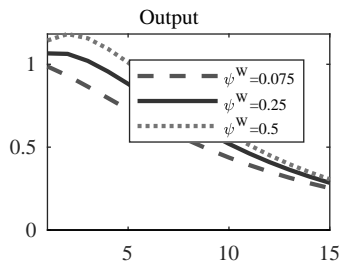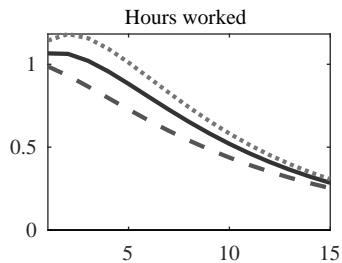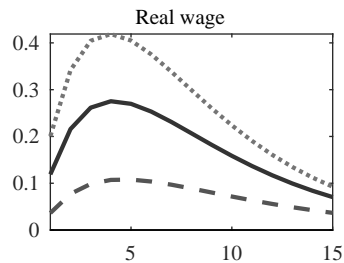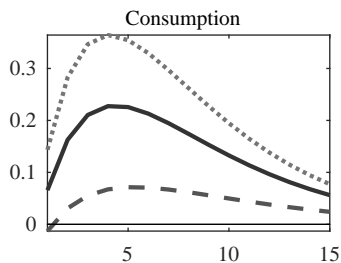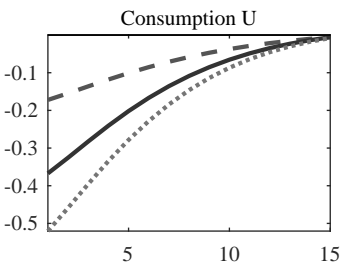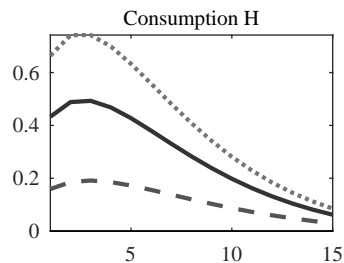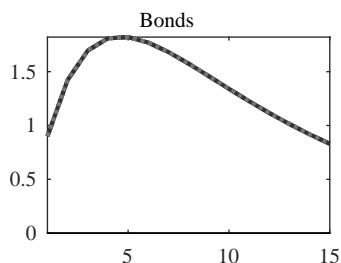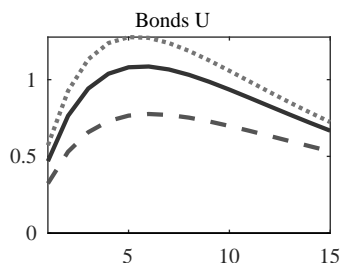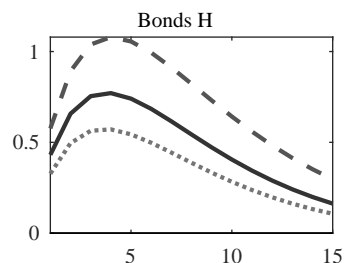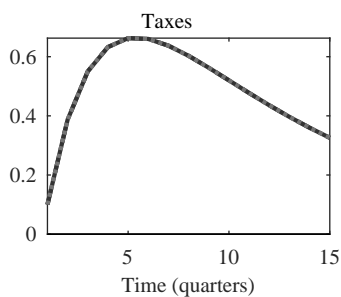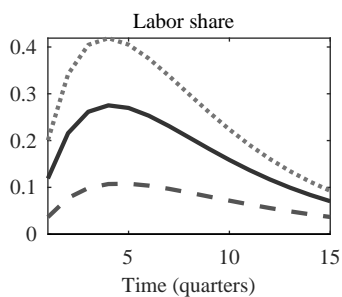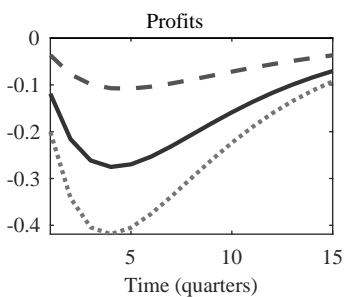

Supplement: Supplementary Data S2 — Supplementary Raw Research Data. This is open data under the CC BY license http://creativecommons.org/licenses/by/4.0/ [file mmc2.zip › TANK-CW_Replication-main/Online appendix/Appendix B/B5-6/Output/fig_sensi_UW_psiw_linear.pdf]

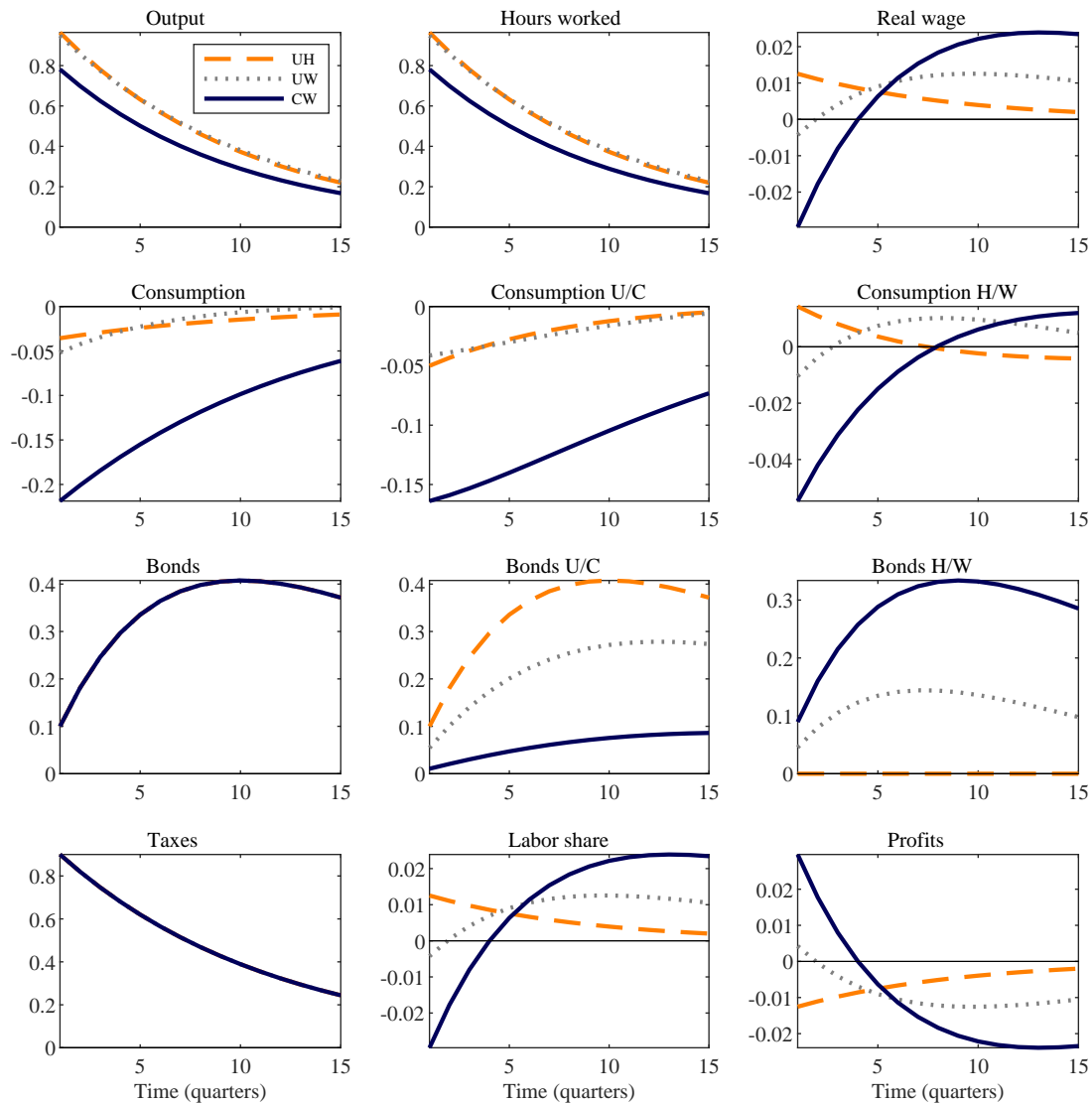

Supplement: Supplementary Data S2 — Supplementary Raw Research Data. This is open data under the CC BY license http://creativecommons.org/licenses/by/4.0/ [file mmc2.zip › TANK-CW_Replication-main/Online appendix/Appendix B/B7/Output/fig5_phib01_phig09.pdf]

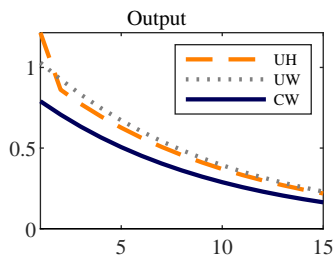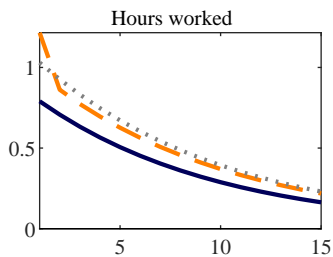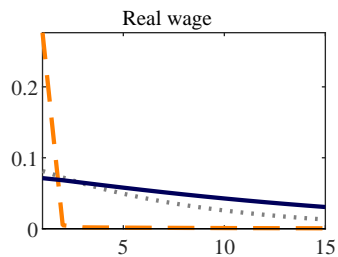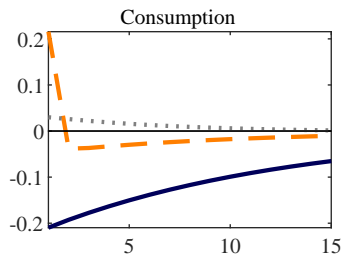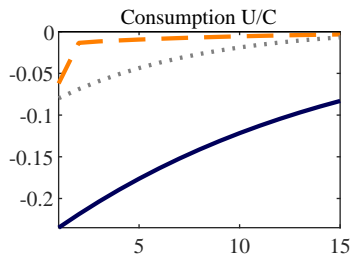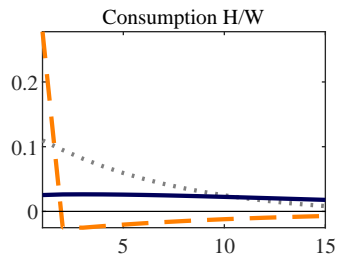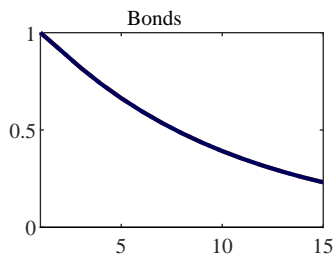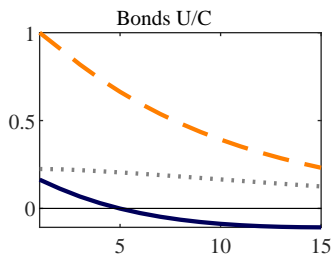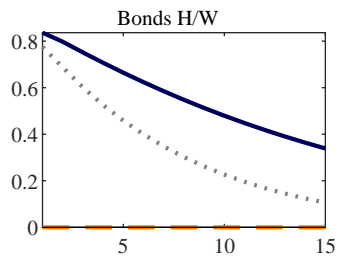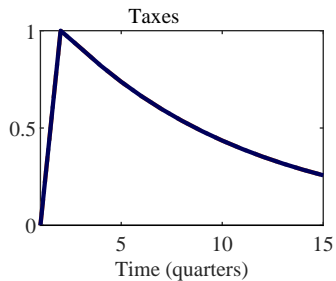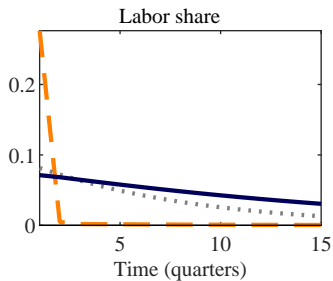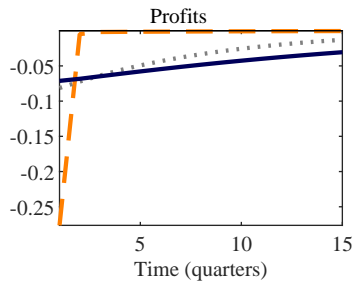

Supplement: Supplementary Data S2 — Supplementary Raw Research Data. This is open data under the CC BY license http://creativecommons.org/licenses/by/4.0/ [file mmc2.zip › TANK-CW_Replication-main/Online appendix/Appendix B/B7/Output/fig5_phib1_phig0.pdf]

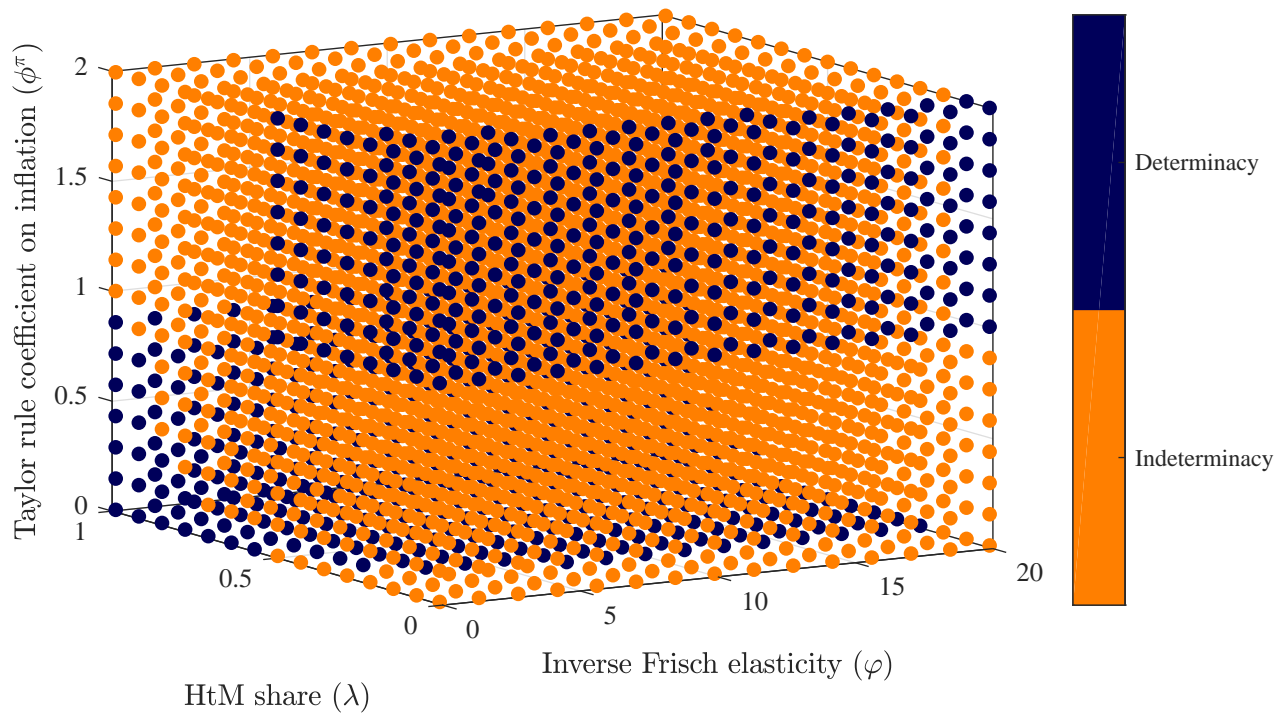

Supplement: Supplementary Data S2 — Supplementary Raw Research Data. This is open data under the CC BY license http://creativecommons.org/licenses/by/4.0/ [file mmc2.zip › TANK-CW_Replication-main/Online appendix/Appendix C/figure C1/Output/fig_C1.pdf]

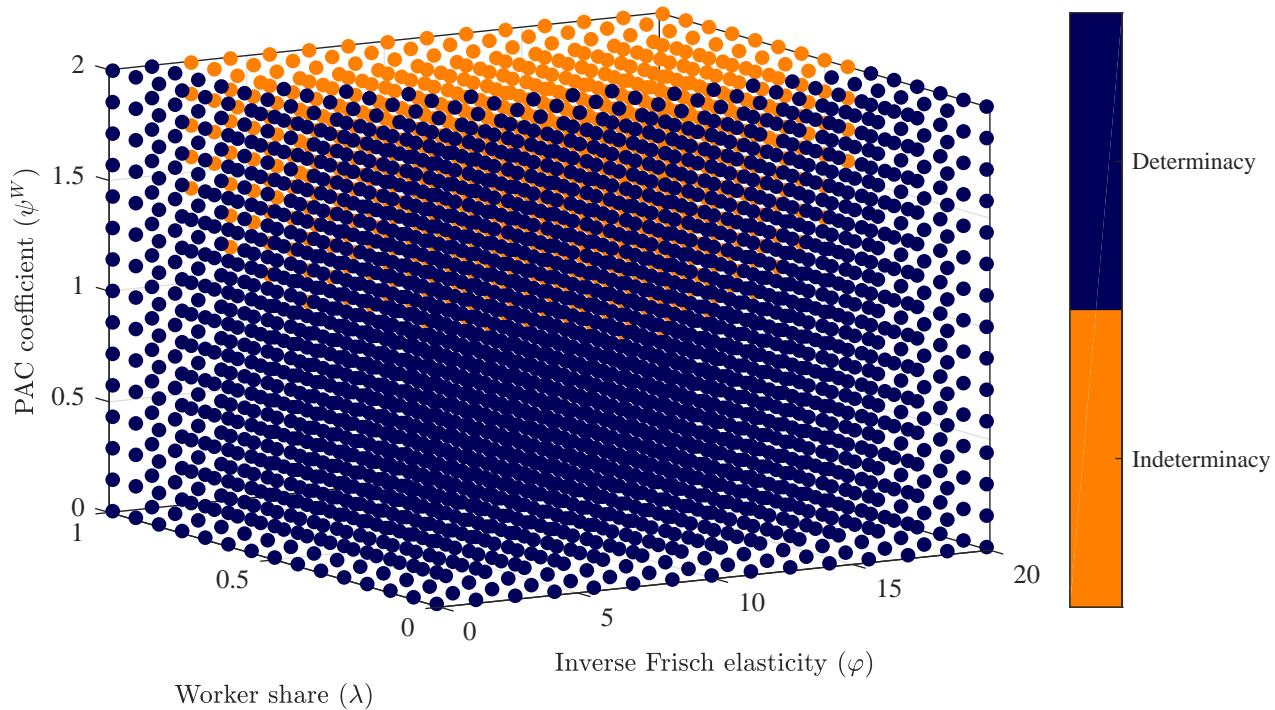

Supplement: Supplementary Data S2 — Supplementary Raw Research Data. This is open data under the CC BY license http://creativecommons.org/licenses/by/4.0/ [file mmc2.zip › TANK-CW_Replication-main/Online appendix/Appendix C/figure C2/Output/Fig_C2a.pdf]

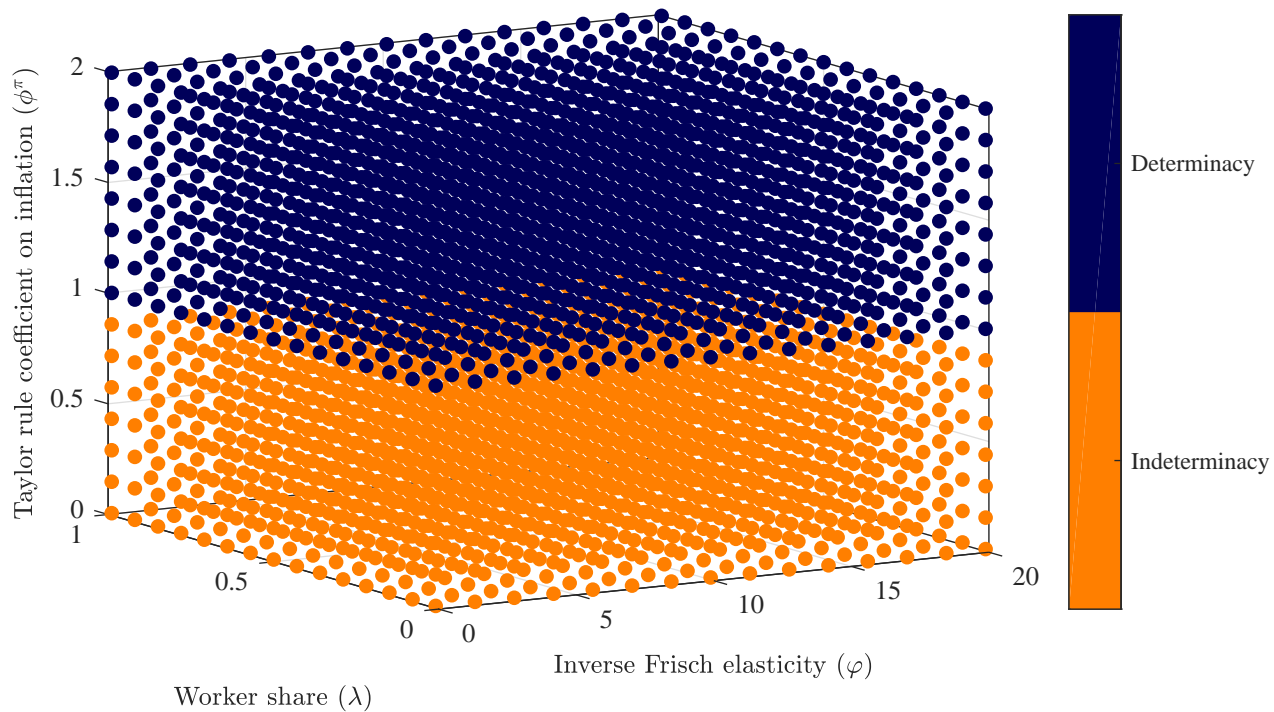

Supplement: Supplementary Data S2 — Supplementary Raw Research Data. This is open data under the CC BY license http://creativecommons.org/licenses/by/4.0/ [file mmc2.zip › TANK-CW_Replication-main/Online appendix/Appendix C/figure C2/Output/Fig_C2b.pdf]

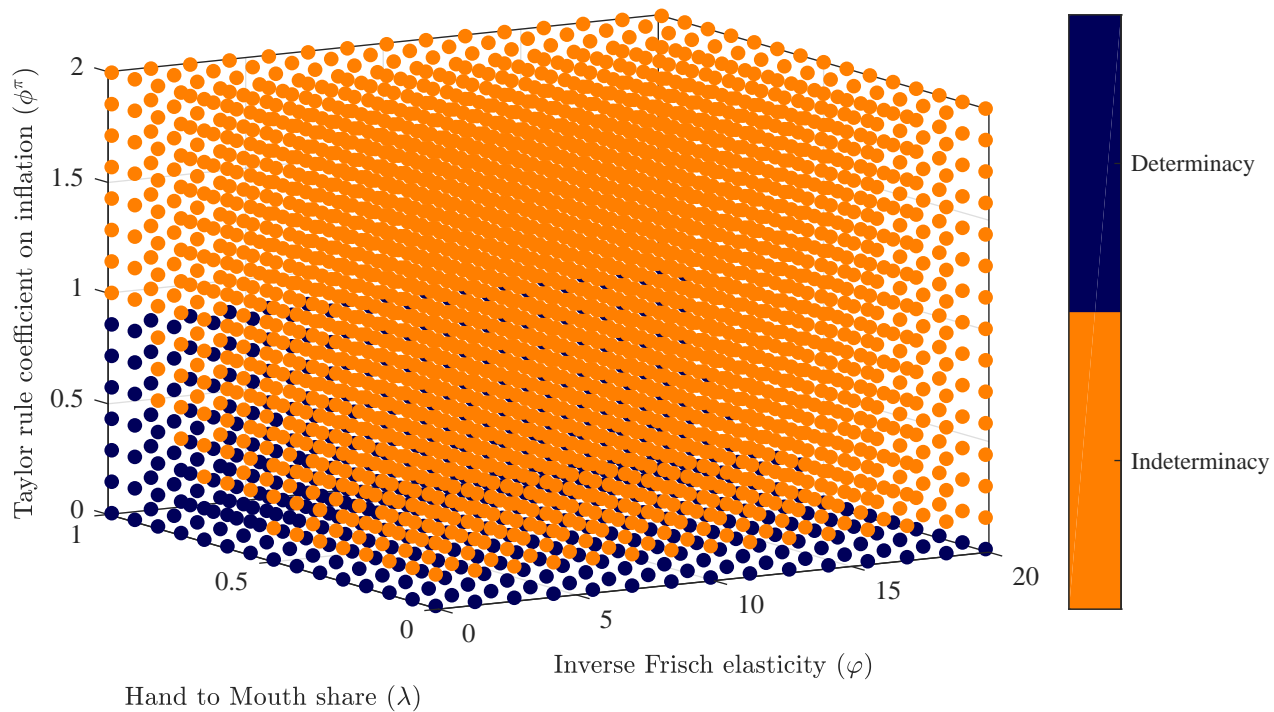

Supplement: Supplementary Data S2 — Supplementary Raw Research Data. This is open data under the CC BY license http://creativecommons.org/licenses/by/4.0/ [file mmc2.zip › TANK-CW_Replication-main/Online appendix/Appendix C/figure C3/Output/Fig_C3a.pdf]

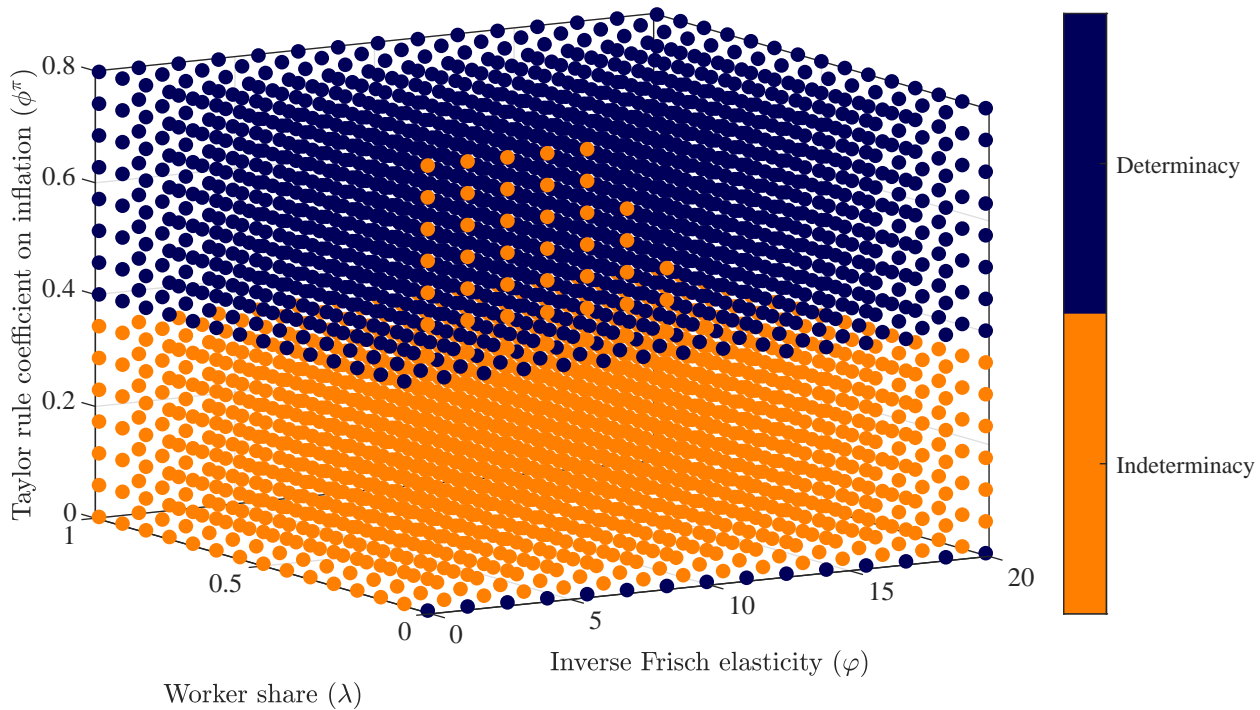

Supplement: Supplementary Data S2 — Supplementary Raw Research Data. This is open data under the CC BY license http://creativecommons.org/licenses/by/4.0/ [file mmc2.zip › TANK-CW_Replication-main/Online appendix/Appendix C/figure C3/Output/Fig_C3b.pdf]

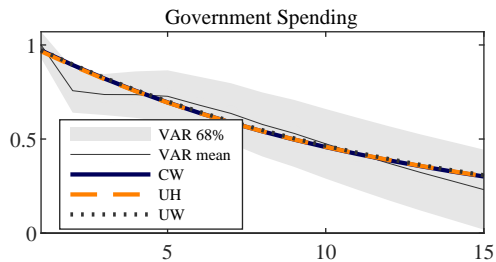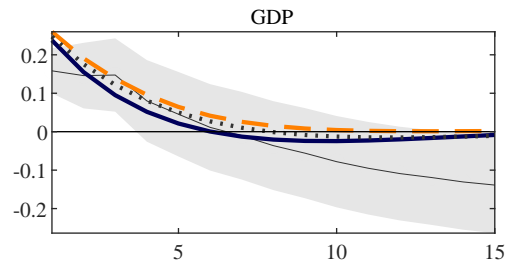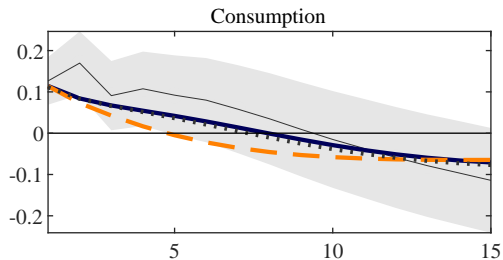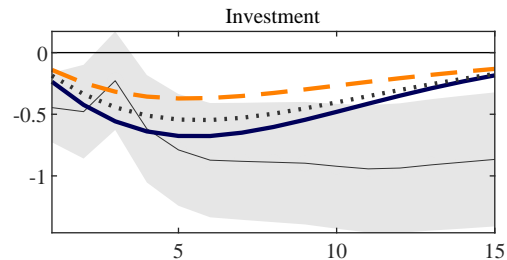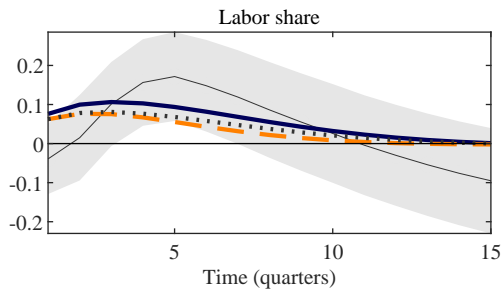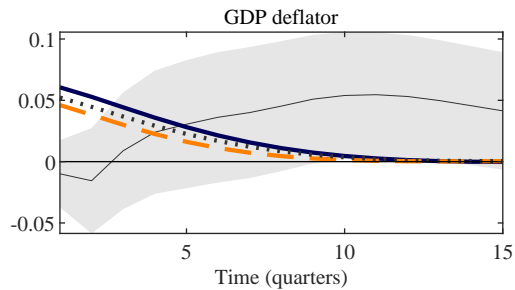

Supplement: Supplementary Data S2 — Supplementary Raw Research Data. This is open data under the CC BY license http://creativecommons.org/licenses/by/4.0/ [file mmc2.zip › TANK-CW_Replication-main/Online appendix/Appendix E/Output/figBIRF.pdf]

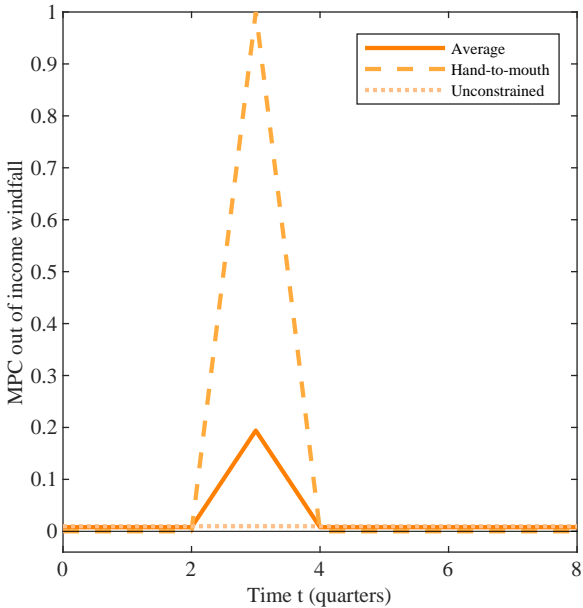

Supplement: Supplementary Data S2 — Supplementary Raw Research Data. This is open data under the CC BY license http://creativecommons.org/licenses/by/4.0/ [file mmc2.zip › TANK-CW_Replication-main/Partial equilibrium iMPCs/Output/fig_iMPCs_Anticipated_H.pdf]

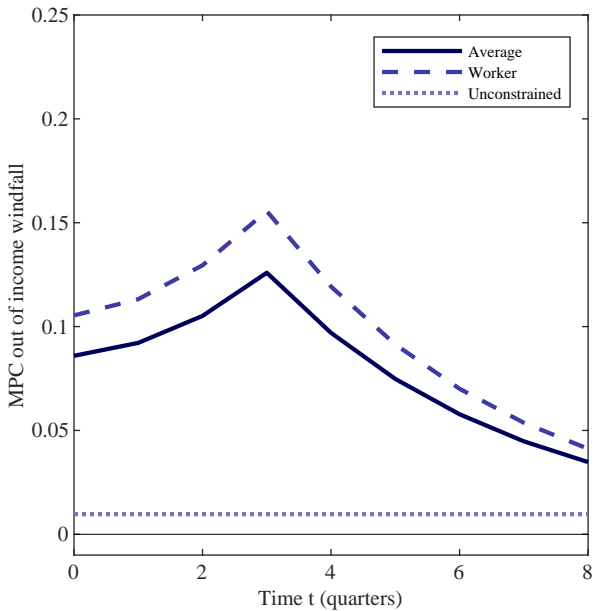

Supplement: Supplementary Data S2 — Supplementary Raw Research Data. This is open data under the CC BY license http://creativecommons.org/licenses/by/4.0/ [file mmc2.zip › TANK-CW_Replication-main/Partial equilibrium iMPCs/Output/fig_iMPCs_Anticipated_W.pdf]

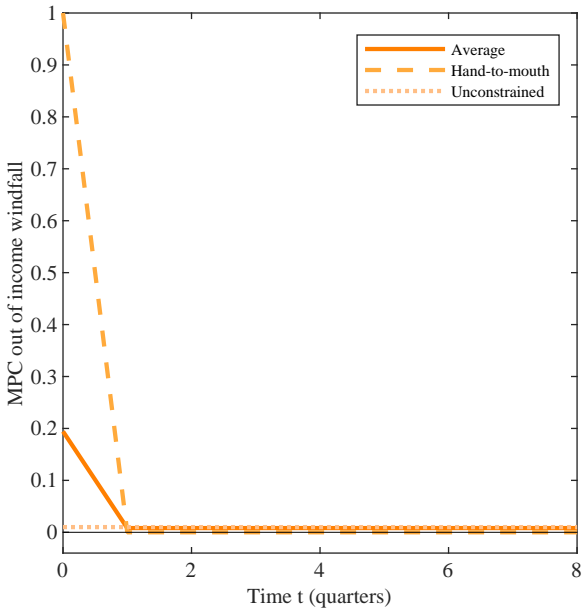

Supplement: Supplementary Data S2 — Supplementary Raw Research Data. This is open data under the CC BY license http://creativecommons.org/licenses/by/4.0/ [file mmc2.zip › TANK-CW_Replication-main/Partial equilibrium iMPCs/Output/fig_iMPCs_Unanticipated_H.pdf]

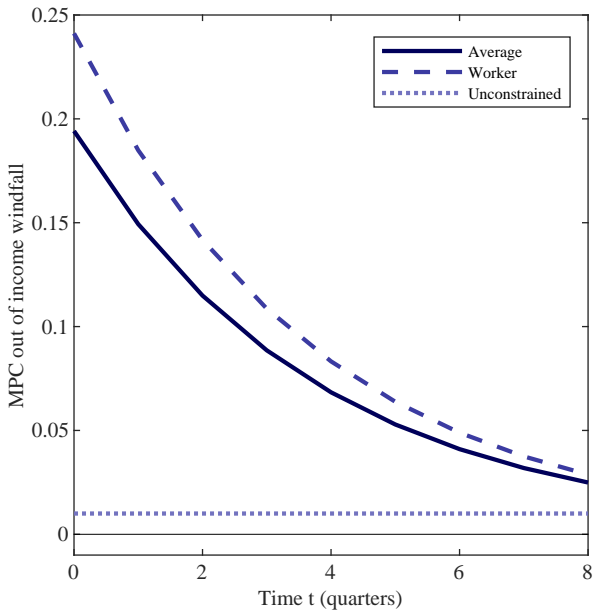

Supplement: Supplementary Data S2 — Supplementary Raw Research Data. This is open data under the CC BY license http://creativecommons.org/licenses/by/4.0/ [file mmc2.zip › TANK-CW_Replication-main/Partial equilibrium iMPCs/Output/fig_iMPCs_Unanticipated_W.pdf]

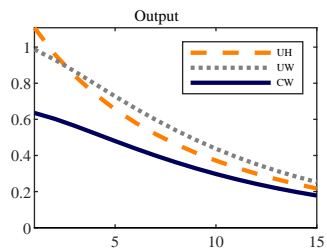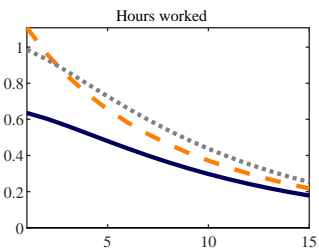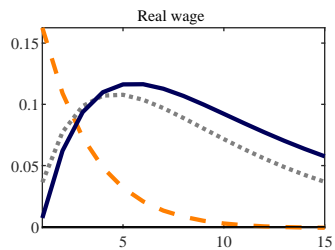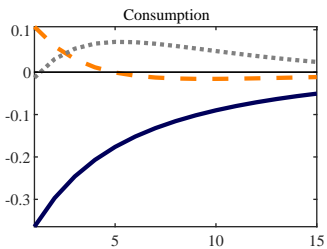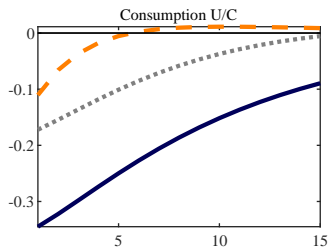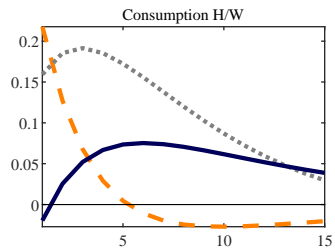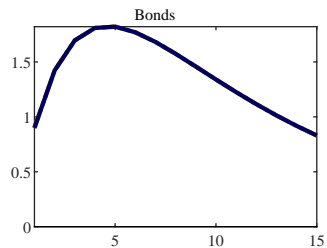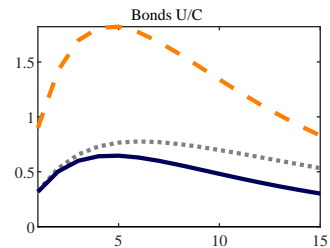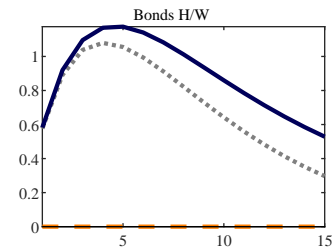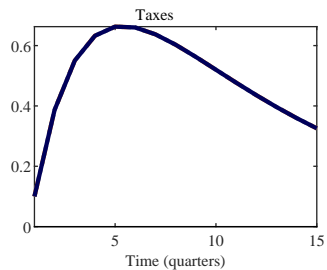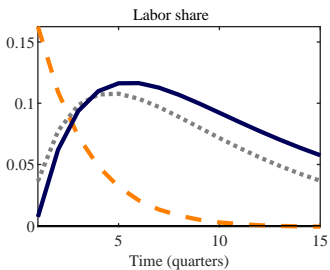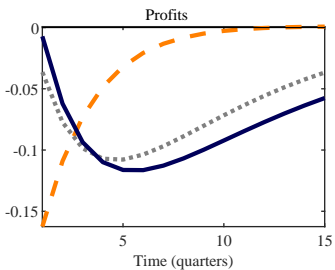

Supplement: Supplementary Data S2 — Supplementary Raw Research Data. This is open data under the CC BY license http://creativecommons.org/licenses/by/4.0/ [file mmc2.zip › TANK-CW_Replication-main/TANK models/fig 5/Output/fig5.pdf]

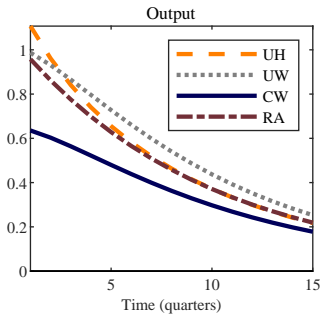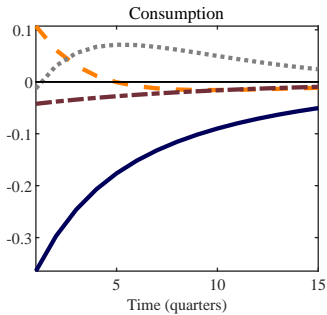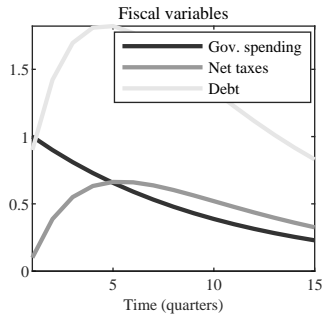

Supplement: Supplementary Data S2 — Supplementary Raw Research Data. This is open data under the CC BY license http://creativecommons.org/licenses/by/4.0/ [file mmc2.zip › TANK-CW_Replication-main/TANK models/fig 6/figure 6a/Output/fig6a.pdf]

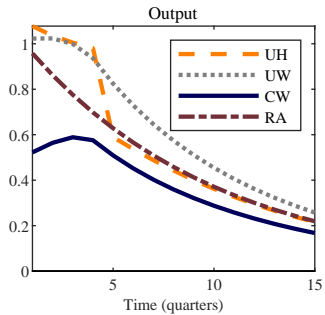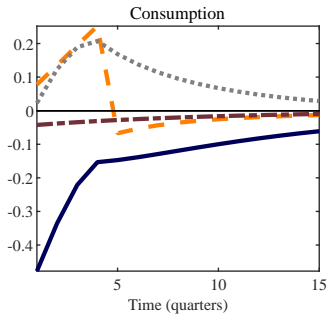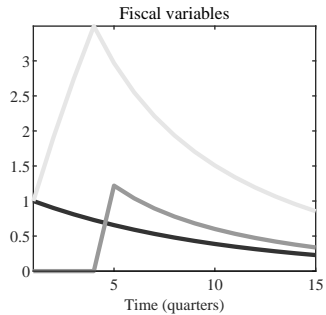

Supplement: Supplementary Data S2 — Supplementary Raw Research Data. This is open data under the CC BY license http://creativecommons.org/licenses/by/4.0/ [file mmc2.zip › TANK-CW_Replication-main/TANK models/fig 6/figure 6b/Output/fig6b.pdf]

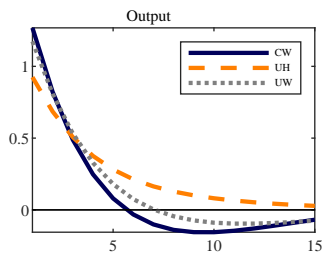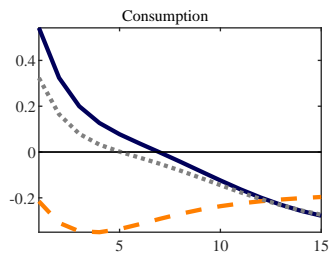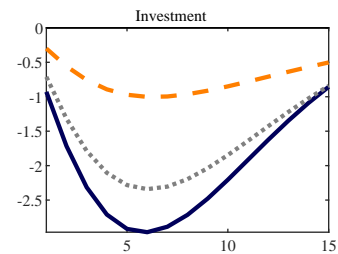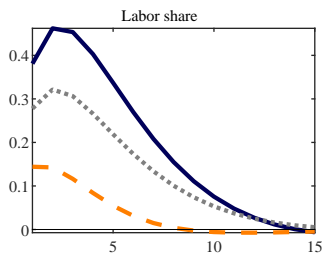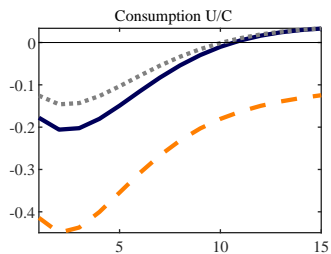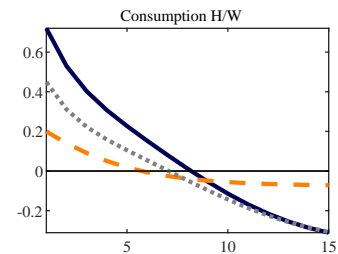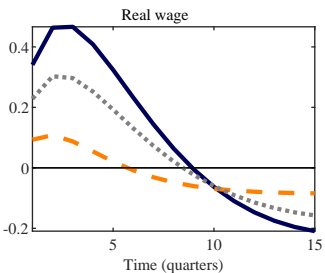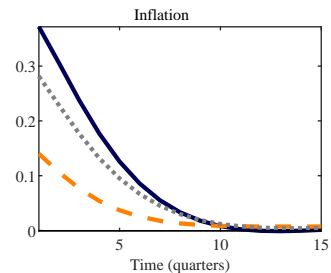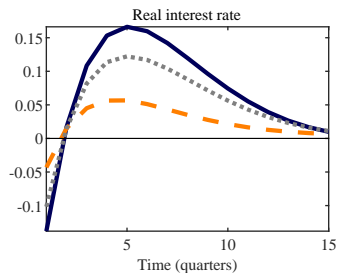

Supplement: Supplementary Data S2 — Supplementary Raw Research Data. This is open data under the CC BY license http://creativecommons.org/licenses/by/4.0/ [file mmc2.zip › TANK-CW_Replication-main/TANK models/fig 7/Output/fig7.pdf]
